# EEG spectral biomarkers of postoperative delirium in spinal surgery: A high-resolution analysis

**DOI:** 10.1371/journal.pone.0352607

**Published:** 2026-07-30

**Authors:** Gengtao Lin, Takahito Uchida, Kota Watanabe, Satoshi Suzuki, Masaya Nakamura, Yasue Mitsukura, Hiroyoshi Takeuchi

**Affiliations:** 1 Graduate School of Science and Technology, Keio University, Yokohama, Kanagawa, Japan; 2 Department of Neuropsychiatry, School of Medicine, Keio University, Tokyo, Japan; 3 Uchida Clinic, Tokyo, Japan; 4 Department of Orthopedic Surgery, School of Medicine, Keio University, Tokyo, Japan; 5 Department of System Design Engineering, Faculty of Science and Technology, Keio University, Yokohama, Kanagawa, Japan; Niigata University of Health and Welfare: Niigata Iryo Fukushi Daigaku, JAPAN

## Abstract

**Background:**

Postoperative delirium is common in older adults following spinal surgery, yet current EEG-based detection methods rely on broad conventional frequency bands that may obscure clinically meaningful oscillatory changes. More granular spectral approaches may reveal candidate frequency-specific features that could improve diagnostic performance.

**Methods:**

We recorded single-electrode frontal EEG (Fp1) from 47 patients at four perioperative timepoints and compared traditional band-level power with high-resolution 1-Hz spectral analysis (1–45 Hz). Group differences were evaluated using non-parametric statistics with FDR correction, and diagnostic accuracy was assessed using AUROC and threshold-optimized metrics. All diagnostics thresholds were derived and evaluated in the same dataset.

**Results:**

Seven patients (14.9%) developed delirium. Conventional band analysis detected only reduced theta power during active delirium (|r| = 0.31; AUROC = 0.53). In contrast, 1-Hz analysis revealed a richer pattern: elevated 1-Hz power (|r| = 0.27), reduced 3–7 Hz power (|r| = 0.25–0.27), and increased 14–15 Hz power (|r| = 0.22–0.26). The 23-Hz narrow-band feature achieved the best discrimination (AUROC = 0.74), despite showing no significant effect within the traditional beta band (12–30 Hz). Several frequencies remained altered even after clinical resolution of delirium.

**Conclusions:**

In this exploratory analysis, high-resolution spectral analysis identified candidate frequency-specific EEG features that distinguished delirium cases more accurately than conventional frequency bands in this cohort. Broadband averaging may not detect frequency-specific oscillatory patterns; adopting finer spectral resolution could enhance the utility of single-channel EEG for routine clinical monitoring.

## Introduction

Postoperative delirium is a frequent and serious complication following spinal surgery, with reported incidence ranging from 8.4% to 40.5% in older adults [1,2]. Yet more than half of cases remain undetected in routine practice. This neuropsychiatric syndrome is characterized by its unpredictable fluctuations. Patients may alternate between agitation and withdrawal, making bedside diagnosis difficult [3]. Undetected delirium contributes to worse outcomes: increased mortality, prolonged hospitalization, long-term cognitive impairment, and a four-fold increase in institutionalization after discharge [4,5]. These consequences also impose substantial economic burdens, with an estimated additional annual cost of $44,291 per case [6].

This problem is poised to intensify. As global populations age, the number of elderly patients undergoing surgery will rise, and with it, the burden of postoperative delirium. Japan offers a clear example of this future. According to the annual report from the Cabinet Office of Japan, it is estimated that people aged 65 and older will make up 34.8% of the population by 2040 [7]. Although studies report delirium in 15–53% of elderly orthopedic patients, national registry data show a diagnosis rate of only 0.2% among surgical cases. Most diagnosed patients (88.2%) require antipsychotic medication, indicating that detection tends to occur only after severe clinical deterioration [8,9]. This gap does not reflect poor clinician awareness but rather fundamental limitations in current delirium assessment methods.

### Current detection paradigms fail to capture delirium’s complexity

The Confusion Assessment Method for Intensive Care Units (CAM-ICU) is a widely used bedside screening tool for delirium diagnosis. Research studies report acceptable performance with pooled sensitivity of 0.78 (95% CI: 0.72–0.83) and specificity of 0.95 (95% CI: 0.92–0.97) [10]; however, its real-world application shows concerning gaps: bedside sensitivity drops to 38% in cardiac surgery ICU patients, while showing limited detection in non-ICU settings [11,12].

Several factors might contribute to the failure. The hypoactive subtype of delirium accounts for 47.2% of cases in elderly surgical patients [13]. These withdrawn patients often go unnoticed because they appear calm and cooperative, not obviously delirious. Also, symptom fluctuations often peak during evening hours (17:00–01:00) when nursing ratios are lowest [14], while most hospitals screen during day shifts. Moreover, the CAM-ICU usually requires 15–20 minutes per assessment and specialized training. One prior work [15] reported that bedside nurses using CAM-ICU detected delirium in only 21.3% of patients compared to 36.7% identified through unstructured clinical assessment, suggesting the tool may underdiagnose delirium when implemented in busy surgical wards.

### EEG monitoring and methodological gaps

Electroencephalography (EEG) provides an objective measure of cortical function and is well-suited for detecting neurophysiological disturbances underlying delirium. Across more than 30 studies, delirium patients showed remarkably consistent EEG spectral patterns: increased relative delta power (1–4 Hz), decreased alpha power (8–13 Hz) with reduction in functional connectivity, and increased theta/alpha ratio in delirious patients [16,17].

The diagnostic value of these spectral biomarkers has been validated across different populations and settings. Van der Kooi et al. achieved a perfect sensitivity of 100% with 96% specificity using relative delta power from a single frontal-parietal derivation (F8-Pz) in 456 cardiothoracic surgery patients [18]. Later multicenter validation by Numan et al. confirmed that single-channel relative delta power measurements maintained strong performance (AUROC = 0.78, 95% CI: 0.72–0.84) across 159 postoperative patients from different surgical specialties [19]. Fleischmann et al. further showed that frequency-specific analysis achieved 100% sensitivity with 95–96% specificity in a large retrospective case-control study [20].

Shinozaki et al. developed a bispectral EEG (BSEEG) device using dual frontal electrodes (Fp1-Fp2) that measures delirium severity through slow-to-fast wave power ratios [21]. Initial validation in 45 patients achieved 80% sensitivity and 87.7% specificity. Later studies in 1,077 delirium patients showed significant mortality prediction (HR = 1.33, 95% CI: 1.16–1.52) [22,23]. The BSEEG score correlates with delirium presence and clinical outcomes including falls, length of stay, and 90-day mortality [24]. Recent improvements have enhanced the BSEEG approach. Topological data analysis applied to raw EEG signals improved classification accuracy by capturing nonlinear dynamics invisible to traditional spectral analysis [25]. The algorithm distinguishes delirium motor subtypes, hyperactive, hypoactive, and mixed, through distinct spectral signatures, addressing variability that confounds behavioral assessments. These single-channel EEG studies have shown strong diagnostic performance, and simplified bispectral devices have demonstrated prognostic value in large cohorts. These findings highlight EEG as a promising candidate for delirium monitoring.

However, nearly all delirium EEG studies rely on conventional frequency bands (delta, theta, alpha, beta, gamma) that average spectral power across broad ranges [26]. This approach assumes pathological activity distributes uniformly within these arbitrary boundaries. Research in other domains shows that high-resolution spectral analysis can identify clinically meaningful oscillatory patterns that are not captured by band-averaged approaches [27].

The spinal surgery population presents distinct neurophysiological responses. One pilot investigation (N = 37) specifically examined patients with spinal surgery, finding paradoxical increases in high-frequency activity rather than the typical slowing pattern associated with delirium [28]. Also, spinal procedures involve unique physiological stressors: prone positioning affecting cerebral perfusion, averaged 800–1200 mL blood loss, prolonged operative duration, and high-dose corticosteroid administration [29,30]. These factors may shape delirium-related brain activity in population-specific ways. Yet no prior study has systematically evaluated high-resolution spectral changes in spinal surgery.

### Study objectives and contributions

The present study addresses these gaps by applying 1-Hz resolution spectral analysis to single-electrode EEG recordings collected across perioperative timepoints. Our objective is to explore (1) frequency-specific spectral differences between delirium and non-delirium patients, (2) the perioperative evolution of these patterns, and (3) the diagnostic potential of narrow-band EEG features compared with traditional frequency bands. This pilot work seeks to determine whether finer spectral resolution can assist delirium detection while preserving the practicality of single-electrode EEG for routine clinical monitoring.

The rest of this paper proceeds as follows: Section 2 details methodology including patient recruitment, EEG acquisition protocols, and spectral analysis techniques. Section 3 presents results examining frequency-specific features and diagnostic performance metrics and discusses findings in context of existing literatures and explores implications for clinical implementation. Finally, the paper is concluded in Section 4 by summarizing the current study.

## Methods

### Study design and participant recruitment

This observational study was conducted by the collaboration between the Department of Neuropsychiatry and the Department of Orthopedic Surgery at Keio University Hospital (Tokyo, Japan). The Ethics Committee of the Medical School of Keio University reviewed and approved the study protocol (IRB approval number: 20180257). All procedures were aligned with the Declaration of Helsinki guidelines for research involving human subjects. Enrolled participants received both written and verbal explanations about the study, and written informed consent were obtained. As compensation for the participation, each person received 3,000 yen upon completing the study.

We recruited patients scheduled for spinal surgery between March 12^th^, 2018 and March 17^th^, 2023. The inclusion criteria are as follows: 1) age at least 60 years old, 2) Mini-Mental State Examination (MMSE) score > 23, and 3) Glasgow Coma Scale (GCS) score > 12. We excluded patients with 1) a history of epilepsy or currently under epilepsy treatment, 2) impaired cognitive capacity (MMSE ≤ 23), and 3) significantly impaired consciousness at baseline (GCS ≤ 12). Additionally, we also excluded subjects with incomplete CAM assessment or EEG recording for the subsequent analysis.

Furthermore, surgical procedures for all participants were performed under general anesthesia using isoflurane (1.5% for induction, 0.5% for maintenance) with lidocaine for local anesthesia at the surgical site. Postoperative analgesia, including acetaminophen and non-steroidal anti-inflammatory drugs, was administered based on clinical need. All postoperative EEG recordings were obtained after complete recovery from anesthesia.

### Clinical assessment and delirium diagnosis

We conducted clinical assessments at four timepoints. T1 occurred preoperatively on the day of hospital admission. T2 took place on postoperative day 1. T3 spanned postoperative days 2–4. T4 covered postoperative days 5–7 or occurred at discharge if patients left earlier. Each timepoint allowed up to 24 hours for completion to accommodate changes in patient availability and clinical status.

Delirium assessment used the confusion assessment method (CAM), an instrument with established validity in surgical populations. The CAM evaluates four cardinal features of delirium: (1) acute onset and fluctuating course, (2) inattention, (3) disorganized thinking, and (4) altered level of consciousness. A positive diagnosis requires features 1 and 2, plus either feature 3 or 4. Trained medical school students conducted all CAM assessments under the supervision of psychiatrists to ensure consistency and minimize variation between raters. We also administered the Glasgow coma scale (GCS), providing an objective measure of consciousness level at each timepoint. Additionally, we asked participants to complete a self-report questionnaire about sleep quality. The assessments taken are summarized in Table A, S1 Appendix.

Medical records provided information on routine clinical tests performed as standard care, including vital signs, blood tests, and brain imaging. We extracted relevant background factors from these records. All data underwent anonymization and secure storage with access restricted to authorized personnel only.

### EEG acquisition

We acquired EEG data using the portable recording system neuroNicle (LAXTHA, Inc., Korea), a single-channel headband device that operates at a sampling frequency of 512 Hz. The device connects via Bluetooth and was monitored using a tablet during recording sessions. The recording electrode was placed at Fp1 (left prefrontal cortex) according to the international 10–20 system, with the reference electrode at A1 (left earlobe). Before each recording, we confirmed that participants were fully alert by checking for eye opening and response to simple commands. If the participant appeared drowsy, we postponed the recording and attempted it later during the observation period.

Each recording session lasted approximately 6 minutes. Participants were asked to sit comfortably in a chair when possible. If pain or medical restrictions prevented sitting, they remained in a supine position. We instructed participants to close their eyes and minimize head and body movements during recording. To maintain alertness and prevent drowsiness, we asked them to count upward mentally from 1 for the first 180 seconds, then count downward from the highest number reached for the next 180 seconds. This mental task maintains arousal without introducing excessive cognitive load or requiring verbal responses that might introduce artifacts. During recording, the specialist monitored participants continuously to ensure eyes remained closed and no sleep occurred. If we observed signs of drowsiness, such as head nodding or lack of response to gentle verbal prompts, we terminated that recording immediately and repeated the measurement after the participant was fully alert.

The real-time artifact removal system automatically identifies and filters body movement artifacts and electrical noise during recording. When the research staff judged that excessive movement or other disturbances compromised recording quality despite the automatic filtering, we repeated the measurements during the observation period to ensure sufficient data quality for subsequent analysis.

### Data preprocessing and spectral analysis

All preprocessing and analysis were done with customized codes in MATLAB R2023a (MathWorks, MA, USA). The raw EEG data were downsampled to 256 Hz and band-pass filtered between 0.5 and 45 Hz using a Butterworth filter. The lower cutoff at 0.5 Hz removed slow DC drift and sweat artifacts. The upper cutoff at 45 Hz excluded high-frequency muscle artifacts and electrical noise while retaining the gamma band. The initial 1-minute recording was removed to ensure stable EEG quality.

We divided the filtered EEG data into non-overlapping 5-second windows. This window length provides sufficient data for reliable spectral estimation while capturing temporal dynamics at clinically relevant timescales. Windows containing excessive artifacts were identified through visual inspection and excluded from further analysis. We discarded any windows where signal amplitude exceeded ±200 μV.

For each 5-second window, we computed the power spectral density using Welch’s method [31]. We applied 1-second Hann window segments with 50% overlap. We then calculated power in five conventional frequency bands: delta (1–4 Hz), theta (4–8 Hz), alpha (8–12 Hz), beta (12–30 Hz), and gamma (30–45 Hz). For each frequency band, we summed the spectral power across all frequency bins within that band to obtain absolute band power. We then calculated total power as the sum of all five band powers. Relative power for each band was computed as the absolute band power divided by total power, expressed as a percentage.

Additionally, we computed power at 1-Hz resolution across the entire spectrum. For each integer frequency f from 1 to 45 Hz, we calculated power in a 1-Hz bin centered at that frequency (spanning from f−0.5 to f+0.5 Hz). We then expressed this power as a percentage of total power, yielding 45 frequency-specific relative power values for each 5-second window. For each subject and timepoint, we averaged the spectral measures across all valid 5-second windows to obtain a single representative spectrum. This averaging reduces the impact of transient fluctuations and provides a stable estimate of the subject’s spectral characteristics at that timepoint. The resulting dataset contained, for each subject at each timepoint, five relative band powers (delta through gamma) and 45 narrow-band relative powers. Absolute spectral power (μV²) was also computed at each timepoint using the same Welch parameters.

### Statistical analysis

We compared EEG spectral features between participants who developed postoperative delirium (delirium group) and those who did not (non-delirium group). Group assignment was based on CAM assessment, with delirium defined as a positive CAM score at each postoperative timepoint (T2, T3, and T4). In practice, all seven delirium cases were identified at T2 (postoperative day 1); no new delirium cases were detected at later timepoints.

For between-group comparisons at each timepoint, statistical inference was performed using Wilcoxon rank-sum test. This non-parametric test does not assume normal distribution and is robust to outliers, making it appropriate for clinical data. We applied this test to both conventional frequency bands (5 comparisons) and 1-Hz resolution data (45 comparisons, one per frequency bin). We calculated effect sizes using the correlation coefficient (r), computed as $r=\;Z\;/\;\sqrt{N}$, where Z is the standardized statistic from the Wilcoxon rank-sum test and N is the total sample size [32,33]. Effect sizes were interpreted according to Gignac and Szodorai’s benchmarks derived from individual differences research, where r = 0.10 represents a small effect, r = 0.20 a medium effect, and r = 0.30 a large effect [34]. These benchmarks, based on meta-analytic distributions of effect sizes, provide more empirically grounded thresholds than Cohen’s traditional conventions. Additionally, to account for multiple comparisons, we applied the Benjamini-Hochberg False Discovery Rate (FDR) correction at q < 0.05 to all spectral analyses [35]. For traditional frequency band analysis (five bands: delta, theta, alpha, beta, gamma), we corrected across the 5 band-wise comparisons at each timepoint. For high-resolution spectral analysis, we corrected across all 45 one-Hz frequency bins at each timepoint. In total, 180 statistical tests were conducted across all four timepoints for the 1-Hz feature analysis (45 frequencies × 4 timepoints), and 20 tests for band analysis (5 bands × 4 timepoints). FDR correction was applied separately within each timepoint. Note that effect size estimates may be unstable despite correction given the small sample size.

Also, we computed bootstrap confidence intervals for group means using 2,000 bootstrap resamples [36]. Bootstrap resampling is a non-parametric approach that makes no assumptions about the underlying distribution of the data, making it particularly suitable for small sample sizes where normality cannot be reliably assessed. For each bootstrap iteration, we randomly sampled with replacement from the original data, calculated the mean, and stored the result. The 95% confidence interval was defined as the 2.5th and 97.5th percentiles of the bootstrap distribution. Importantly, bootstrap resampling was used solely for confidence interval estimation.

As a post-hoc sensitivity analysis, we tested whether the spectral effects from the primary comparisons remained after accounting for demographic factors. We used analysis of covariance (ANCOVA) with group as the between-subjects factor and age and sex as covariates. Baseline MMSE was kept as a descriptive variable rather than a covariate, since the inclusion criterion of MMSE > 23 restricts its variability across the cohort. This analysis was applied only to features that reached significance after FDR correction.

To examine temporal change of spectral features, we performed paired comparisons between timepoints within the delirium group. We compared T1 (preoperative baseline) against each postoperative timepoint (T2, T3, T4) using the Wilcoxon signed-rank test for each delirium subject.

For diagnostic performance evaluation, we calculated sensitivity, specificity, positive predictive value (PPV), negative predictive value (NPV), and likelihood ratios for selected EEG features [37]. We classified each observation as delirium or non-delirium based on whether its EEG feature value exceeded the optimal threshold. This classification produced four outcomes: true positives (TP, delirium cases correctly identified as delirium), true negatives (TN, non-delirium cases correctly identified as non-delirium), false positives (FP, non-delirium cases incorrectly classified as delirium), and false negatives (FN, delirium cases incorrectly classified as non-delirium). Thus, we computed aforementioned diagnostic metrics by:


$$\begin{array}{c} Sensitivity=\frac{TP}{TP+FN} \end{array}$$ (1)

$$\begin{array}{c} Specificity=\frac{TN}{TN+FP} \end{array}$$ (2)

$$\begin{array}{c} PPV=\frac{TP}{TP\;+FP\;} \end{array}$$ (3)

$$\begin{array}{c} NPV=\frac{TN}{TN+FN} \end{array}$$ (4)

$$\begin{array}{c} Positive\;Likelihood\;ratio\;(LR+)=\frac{Sensitivity}{\text{1}-Specificity} \end{array}$$ (5)

$$\begin{array}{c} Negative\;Likelihood\;ratio\;(LR-)=\frac{\text{1}-Sensitivity}{Specificity} \end{array}$$ (6)

We determined optimal classification thresholds using Youden’s J statistic, J=Sensitivity+Specificity−1, and constructed receiver operating characteristic (ROC) curves to compute the area under the curve (AUROC) [38,39]. Bootstrapped confidence intervals also provided uncertainty estimates for these metrics.

## Results and discussion

The following section presents findings and our interpretation for the present study. We begin with cohort characteristics and delirium incidence, then examine spectral differences using conventional frequency bands and high-resolution 1-Hz analysis, within-subject temporal changes, and finally diagnostic performance through ROC analysis.

### Study cohort and demographic characteristics

**Fig 1:**
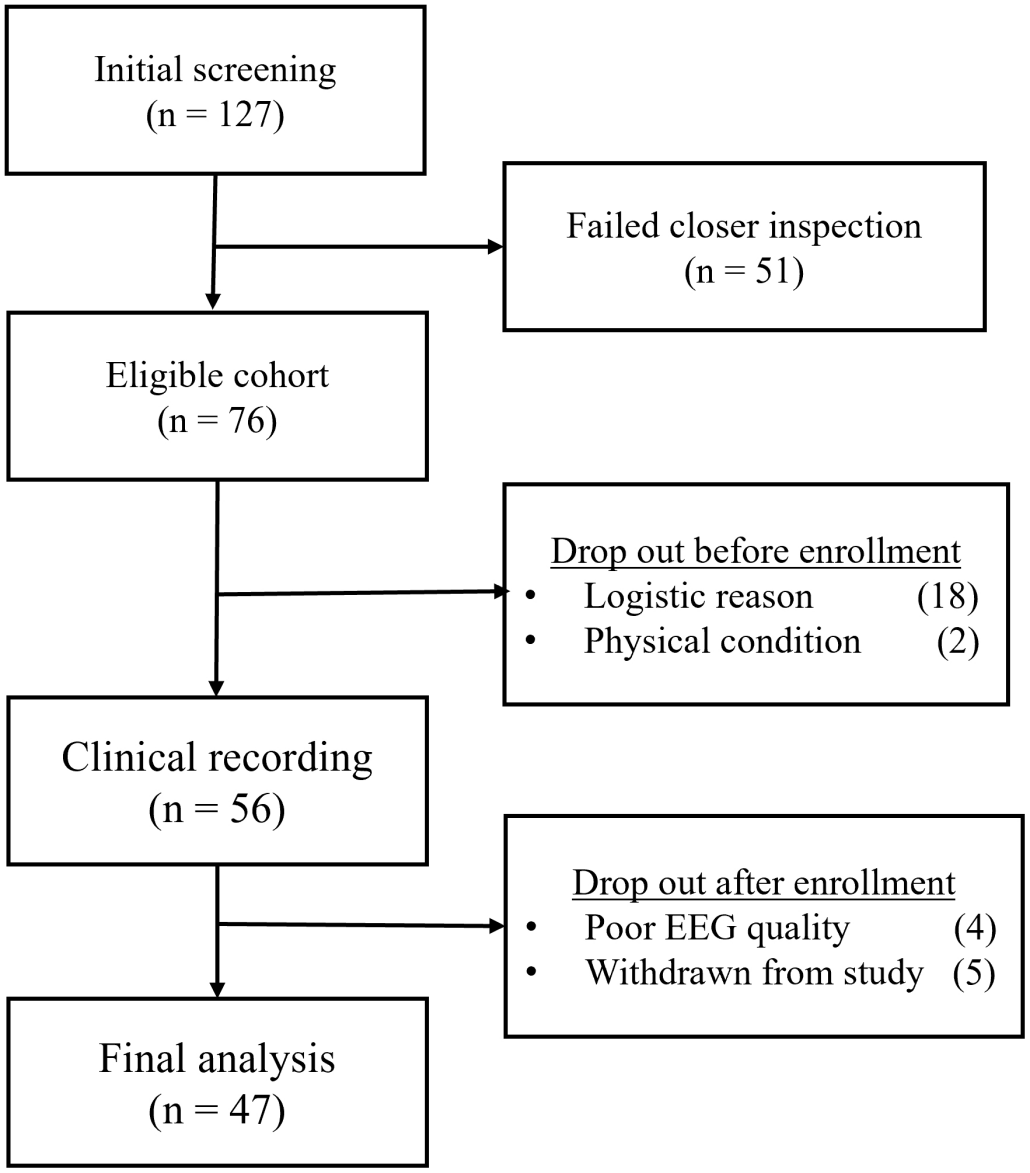
Cohort screening flow.

Initial screening identified 127 potential participants, of whom76 met the inclusion criteria. Prior to the clinical recording, 18 participants were excluded due to logistical constraints, and two participants became physically unable to provide testing due to unexpected medical complications. After recording, 9 participants either withdrew from postoperative measurements or produced EEG signals too unclear for reliable analysis. Our final analysis included 47 patients. The screening process is summarized in Fig 1 below.

Forty-seven patients completed the study with analyzable EEG recordings. Seven patients (14.9%) developed postoperative delirium based on CAM assessment. The remaining 40 patients showed no delirium throughout the observation period. Table 1 presents demographic and clinical characteristics of both groups. Mean age was comparable between non-delirium (72.9 ± 7.2 years) and delirium (73.4 ± 7.8 years) groups. Baseline MMSE scores were similar between the non-delirium (28.8 ± 1.1) and delirium (28.4 ± 1.0) groups (p = 0.45). The delirium group had a higher proportion of male participants (71.4%) compared to the non-delirium group (52.5%), though the small sample size of the delirium group limits interpretation of this difference. Relevant medical history includes hypertension, heart disease, diabetes, and prior delirium episodes.

**Table 1:** Demographic details of participants.

	Non-Delirium (*N* = 40)	Delirium (*N* = 7; 14.9%)
**Sex**		
Male	21 (52.5%)	5 (71.4%)
Female	19 (47.5%)	2 (28.6%)
Mean Age (SD)	72.9 (7.2)	73.4 (7.8)
**Relevant medical history**		
Delirium	1 (2.5%)	0 (0.0%)
Hypertension	6 (15.0%)	1 (14.3%)
Heart disease	1 (2.5%)	0 (0.0%)
Diabetes	2 (5.0%)	0 (0.0%)

EEG data completeness varied across the four assessment timepoints. Data availability decreased at subsequent time points due to patient fatigue, medical procedures, early discharge, or signal quality issues. The details of participants with valid EEG data at each timepoint are presented in Table B, S1 Appendix. Spectral analysis was performed on 5-second non-overlapping EEG windows, with distribution across timepoints summarized in Table 2 below.

**Table 2:** Data distribution of 5-second EEG windowed data.

	T1	T2	T3	T4
Non-delirium	2102	1929	1522	1268
Delirium	423	420	120	120

The observed delirium incidence of 14.9% falls within the range of published estimates for spinal surgery populations (8.4% − 40.5%). All seven delirium cases showed positive CAM scores only at T2 (postoperative day 1). By T3 and T4, these patients had returned to CAM-negative status. This pattern suggests that delirium in our cohort was acute and resolved within 24–48 hours. At T1 and T2, all 7 delirium patients contributed EEG data; however, by T3 and T4, only 2 delirium patients remained hospitalized with analyzable recordings. This drop limits the reliability of later timepoint findings.

### Between-group EEG spectral comparisons

We compared relative power between groups using standard frequency bands: delta (1–4 Hz), theta (4–8 Hz), alpha (8–12 Hz), beta (12–30 Hz), and gamma (30–45 Hz). Statistical tests used Wilcoxon rank-sum test with FDR correction. Effect sizes are reported as correlation (r), where |r| < 0.1 is small, 0.1 ≤ |r| ≤ 0.3 is typical, and |r| ≥ 0.3 is relatively large.

**Fig 2:**
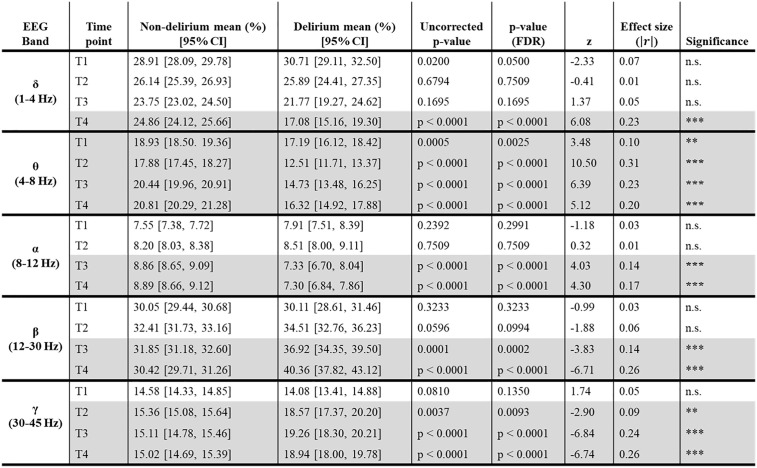
Table of EEG band power comparison between groups. * Effect size |r| – correlation; significance markers: *n.s.* – not significant; *: p < 0.05, **: p < 0.01, ***: p < 0.001.

**Fig 3:**
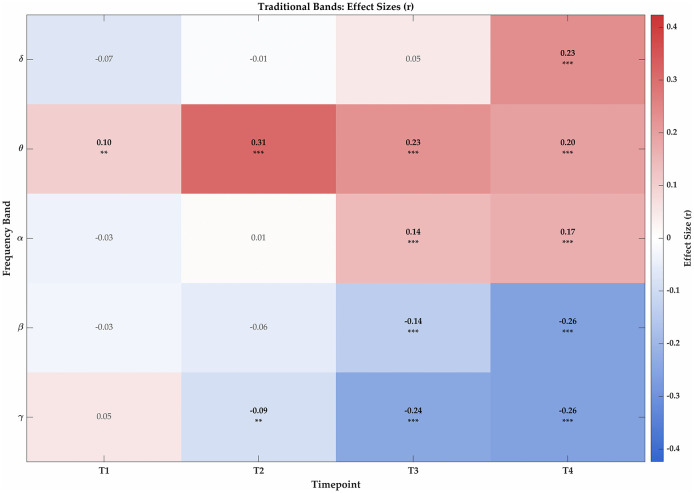
Heatmap of effect sizes for EEG frequency band comparisons across timepoints. Effect sizes are expressed as standardized effect size (r), with positive values (red) indicating higher power in the non-delirium group and negative values (blue) indicating higher power in the delirium group. Rows represent frequency bands (delta, theta, alpha, beta, gamma) and columns represent timepoints (T1-T4). Significant differences after FDR correction are marked with: *p < 0.05, **p < 0.01, ***p < 0.001.

Fig 2 and Fig 3 below summarize conventional frequency band comparisons across all time points. Detailed boxplots for EEG band are shown in S1–S5 Figs. At preoperative baseline (T1), only theta band power differed significantly between groups. Non-delirium patients demonstrated higher theta power than those who later developed delirium (18.93% [95% CI: 18.52–19.37] compared with 17.19% [16.09–18.35], r = 0.10, p = 0.003). Delta, alpha, and beta bands showed no significant differences.

During clinical delirium on postoperative day 1 (T2), two frequency bands showed significant differences between-groups. Theta power was markedly lower in delirium patients compared to controls (12.51% compared with 17.88%, r = 0.31, p < 0.001), representing a medium effect size. Gamma power was modestly elevated in delirium patients (18.57% compared with 15.36%, r = 0.09, p = 0.009). Meanwhile, delta, alpha, and beta bands showed no significant differences between-groups.

At later timepoints, spectral differences persisted. At T3, theta band power remained significantly lower in the delirium group (14.73% compared with 20.44%, r = 0.23, p < 0.001), alpha power was reduced (7.33% compared with 8.86%, r = 0.14, p < 0.001). In addition, both beta (36.92% compared with 31.85%, r = −0.14, p < 0.001) and gamma (19.26% compared with 15.12%, r = −0.24, p < 0.001) were elevated. By timepoint T4, all five frequency bands showed significant differences, with the delirium group demonstrating lower delta, theta, and alpha alongside higher beta and gamma (summarized in Fig 2). These T3-T4 findings, however, derive from only 2 delirium patients and require cautious interpretation.

The reduction in theta power during active delirium differs from commonly reported findings. Most prior studies in cardiac surgery and ICU populations report elevated theta. Several factors warrant consideration before attributing this divergence to population-specific physiology alone. The EEG recordings were obtained during a brief mental counting task, which is known to modulate frontal theta activity, and differential task engagement between groups cannot be ruled out as a contributing factor. On the methodological side, relative and absolute power showed the same direction of theta reduction at T2 (see Table E in S1 Appendix). Within these caveats, findings are consistent with Lee et al. (2022), who also reported theta suppression accompanied by beta and gamma increases in spinal surgery patients [28]. Together, these results suggest that delirium in spinal surgery represents a distinct physiological context. Factors such as prone positioning, fluctuating cerebral perfusion, substantial blood loss, and perioperative steroid administration may contribute to this different spectral signature.

Delta power did not provide meaningful discriminative value in our study. This observation is notable because most previous EEG-based delirium research reports delta-dominant patterns. The lack of delta discrimination challenges the assumption that broad frequency bands function as coherent physiological units. Clinically meaningful information can be obscured when heterogeneous sub-frequencies are averaged.

**Fig 4:**
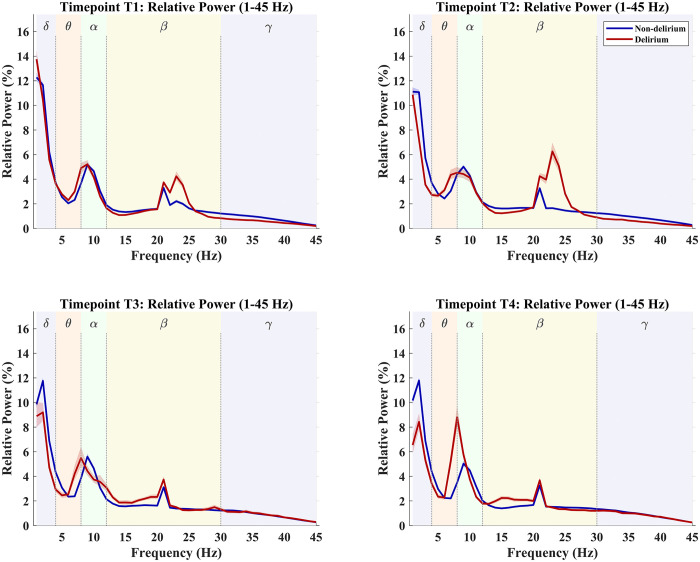
High-resolution (1-Hz) spectral power comparisons between delirium and non-delirium groups at T1 (top-left panel), T2 (top-right panel), T3 (bottom-left panel), and T4 (bottom-right panel). Relative power is plotted for each 1-Hz frequency bin from 1 to 45 Hz. Blue and red lines represent mean values for non-delirium and delirium groups, respectively, with shaded areas indicating 95% confidence intervals. Background shading denotes traditional frequency bands: delta (1-4 Hz), theta (4-8 Hz), alpha (8-12 Hz), beta (12-30 Hz), and gamma (30-45 Hz).

**Fig 5:**
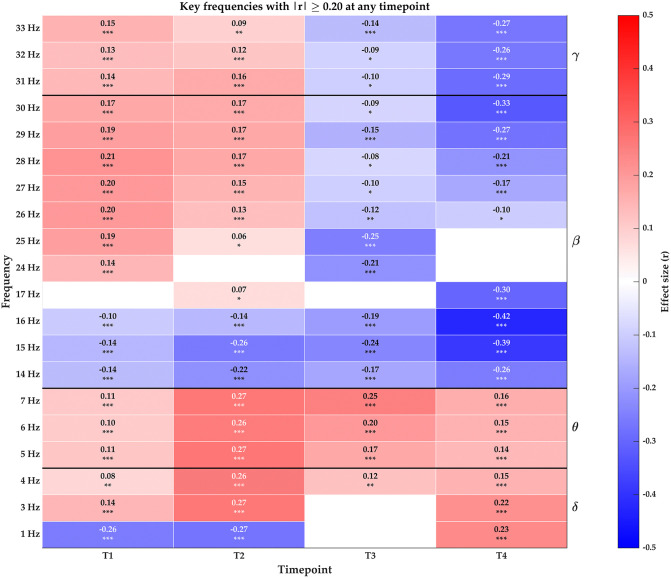
Key spectral effects across timepoints (1-Hz resolution). Heatmap showing the 20 frequencies with medium or larger effect sizes (|r| ≥ 0.20) at one or more timepoints, selected from the full 1–45 Hz spectrum (see S6 Fig for the complete heatmap). Positive r values (red) indicate higher power in the non-delirium group; negative r values (blue) indicate higher power in the delirium group. Rows represent individual frequencies grouped by conventional band (δ, θ, β, and γ; α band is not shown as no frequency in this band met the inclusion threshold), and columns represent timepoints (T1–T4). Cells are blank where the comparison was not significant after FDR correction or where |r| < 0.20 at that timepoint. Significant differences after FDR correction are marked with: *p < 0.05, **p < 0.01, ***p < 0.001. Note: T3 and T4 columns include only two delirium patients due to discharge attrition and should be interpreted with caution.

To further explore within-band differences, we applied 1-Hz resolution analysis across the 1–45 Hz spectrum (as shown in Fig 4). All reported findings survived FDR correction at q < 0.05. Frequencies showing medium or larger effect sizes (|r| ≥ 0.20) at any timepoint are summarized in Fig 5, with the full 1–45 Hz effect size pattern provided in S6 Fig and detailed statistics in Table C in S1 Appendix. Additionally, absolute spectral power values for all timepoints are presented in Supplementary Table D in S1 Appendix.

At preoperative baseline, we found 26 discrete frequencies were significantly different between non-delirium and delirium group. The most prominent finding was elevated 1 Hz power in patients who later developed delirium (7.12% [6.18–8.22] compared with 3.81% [3.31–4.34], |r| = 0.26, p < 0.001), while the 3 Hz frequency was lower in the delirium-prone group (8.05% compared with 9.75%, |r| = 0.14, p < 0.001). Within the beta band range, frequencies from 21–30 Hz showed consistently lower power in patients who later developed delirium, with the largest effects at 27–28 Hz (|r| = 0.20−0.21). Interestingly, the 14–16 Hz low-beta range showed the opposite pattern, with elevated power in delirium-prone patients (15 Hz: 2.16% compared with 1.51%, |r| = 0.14, p < 0.001). High gamma frequencies were also elevated preoperatively in those who later developed delirium (45 Hz: 1.32% compared with 1.08%, |r| = 0.19, p < 0.001).

At T2, the 1-Hz analysis revealed a distinctive spectral reorganization involving 27 discrete frequencies (Table C in S1 Appendix presents all frequencies with |r| ≥ 0.1). The pattern can be characterized across several spectral domains. At the ultra-slow frequency end, the 1 Hz frequency showed elevation in delirium patients (6.06% compared with 2.51%, |r| = 0.27, p < 0.001). In the mid-frequency range, 3–7 Hz showed consistent reductions with effect sizes largest at 3, 5, and 7 Hz (all |r| ≈ 0.27, p < 0.001), while 8 Hz showed a smaller but significant reduction (|r| = 0.18). Low-beta frequencies (13–16 Hz) were elevated in delirium patients (15 Hz: 3.43% compared with 2.19%, |r| = −0.26, p < 0.001). Conversely, high-beta frequencies (26–32 Hz) were consistently lower in delirium patients (28 Hz: 0.78% compared with 0.95%, |r| = 0.17, p < 0.001). At the highest frequencies, gamma activity (41–45 Hz) showed pronounced elevation, with 45 Hz demonstrating significantly higher power in delirium patients (2.29% compared with 0.97%, |r| = 0.18, p < 0.001). The overall pattern shows elevated activity at the spectral extremes combined with reduced mid-frequency power (3–8 Hz). This pattern is consistent with complex cortical reorganization during delirium rather than simple global slowing, though cautious interpretation is warranted given the small sample size. An alternative explanation is that differential engagement with the mental-counting task between groups may have contributed to the observed theta differences; future resting-state recordings would help clarify the problem.

At T3 and T4, spectral differences persisted and intensified despite clinical delirium resolution. The largest effects at T4 were observed at 15 Hz (|r| = 0.39, p < 0.001) and 16 Hz (|r| = 0.42, p < 0.001), with delirium patients showing significant increase in EEG power (15 Hz: 4.94% compared with 1.41%; 16 Hz: 6.57% compared with 1.86%). These findings, however, were derived from only 2 patients and could not be generalized.

### Within-subject temporal changes in delirium patients

To examine whether the between-group spectral differences reflect consistent individual-level changes, we analyzed within-subject EEG power changes in the delirium group, comparing postoperative timepoints against preoperative baseline (T1). All seven delirium patients had paired T1-T2 data, while only 2 patients (Subject 11 and Subject 23) remained hospitalized with recordings at T3 and T4 (Table B in S1 Appendix).

In the analysis of traditional EEG band power, from T1 to T2 (during active delirium), spectral changes showed notable consistency across individuals despite inter-patient variability in absolute values. We observed a decrease in theta power in 6 out of 7 patients, with reductions ranging from −12.8% to −41.5%. One notable exception is that one patient (Subject 25) showed increased theta (+12.2%) and a highly atypical profile with a 334% gamma increase, suggesting a possible distinct pathophysiology. Additionally, we found beta power increased in 5 out of 7 patients, and gamma increased in 4 of 7 patients. Delta and alpha showed more variable patterns, with approximately half of patients showing increases and half showing decreases. At later timepoints (T3 and T4), despite the reduced number of recordings, the two patients with available data both showed persistent theta reduction and beta elevation compared to their own preoperative baselines, despite CAM-negative status at these timepoints. Table 3 below shows the proportion of participants showing increasing or decreasing EEG power for all bands.

**Table 3:** Direction of spectral change from baseline (T1) to delirium onset (T2).

EEG band	Increased (n)	Decreased (n)	Consistency
**Delta**	3/7	4/7	57%
**Theta**	1/7	6/7	86%
**Alpha**	5/7	2/7	71%
**Beta**	5/7	2/7	71%
**Gamma**	4/7	3/7	57%

Given the strong individual-level consistency observed for theta and beta band power, we examined these bands at 1-Hz resolution to identify which specific frequencies drove these changes. Table 4 summarizes the most consistent changes from T1 to T2.

**Table 4:** Individual-level spectral changes at 1-Hz resolution power (from T1 to T2).

EEG Frequency	Decreased (n)	Increased (n)	Consistency	Mean % Change
**Theta Range**				
4 Hz	6/7	1/7	86% ↓	−25.9%
5 Hz	6/7	1/7	86% ↓	−26.8%
6 Hz	6/7	1/7	86% ↓	−22.1%
7 Hz	5/7	2/7	71% ↓	−17.9%
8 Hz	5/7	2/7	71% ↓	−11.0%
Low-beta Range				
13 Hz	1/7	6/7	86% ↑	+96.9%
14 Hz	1/7	6/7	86% ↑	+114.4%
15 Hz	2/7	5/7	71% ↑	+110.5%
High-beta Range				
25 Hz	1/7	6/7	86% ↑	+53.0%
27 Hz	1/7	6/7	86% ↑	+36.8%
28 Hz	1/7	6/7	86% ↑	+30.3%
29 Hz	1/7	6/7	86% ↑	+27.9%

Within the theta range, the lower frequencies (4–6 Hz) showed the most consistent reductions (86%), while 7–8 Hz showed somewhat less consistency (71%). This gradient suggests that the within-subject theta change is driven primarily by the 4–6 Hz sub-band rather than the theta band as a whole. Since conventional band averaging treats 4–8 Hz as a single unit, this internal structure would not be visible without 1-Hz resolution. Lower theta frequencies have been studied extensively in the context of memory and cognition using invasive and multi-channel recordings [40]; whether the 4–6 Hz reduction we observed at Fp1 reflects any of these processes cannot be determined from a single-electrode signal and would require source-localized or multi-channel recordings to address.

Furthermore, within the beta range, the individual-level analysis revealed an intriguing pattern. Low-beta frequencies (13–15 Hz) increased consistently (71–86% of patients), as did high-beta frequencies (25–30 Hz, 71–86% of patients); however, we observed mid-beta frequencies (18–21 Hz) showing mixed responses. When averaged across the full 12–30 Hz band, the consistent low- and high-beta increases are partially cancelled by the variable mid-beta responses, which likely contributes to the moderate consistency observed at the band level. Different beta sub-ranges have been associated with distinct sensorimotor and cognitive processes in prior work [41,42], but linking our finding to any specific process, or to clinical phenotypes, such as hyperactive and hypoactive presentations, would require multi-channel recordings together with motor or cognitive assessments.

Although data from T3 and T4 were limited, recordings from the two patients who remained hospitalized showed persistent abnormalities, particularly increases around 15–16 Hz (|r| = 0.39− 0.42); however, within the scope of the current study, we cannot determine that delirium involves ongoing neurophysiological disruption after clinical symptoms resolve. Future studies with larger samples and sustained postoperative follow-up are needed to confirm whether these abnormalities are reproducible and whether they carry prognostic value. Thus, these persistent abnormalities at T3 and T4 are reported as descriptive observations from two patients only; the following diagnostic performance in the next section was therefore evaluated solely at timepoint T2.

Since the delirium and non-delirium groups differed in gender composition (71.4% compared with 52.5% male) and showed comparable but not identical age distributions, we re-evaluated all features that reached significance after FDR correction using ANCOVA with age and sex as covariates. Results are reported as F statistics with partial η².

At the band level, neither the T1 theta difference (F(1,38) = 2.43, p = 0.128, partial η² = 0.060) nor the T2 theta reduction (F(1,35) = 0.76, p = 0.388, partial η² = 0.021) remained significant after adjustment. T2 gamma did survive (F(1,35) = 4.46, p = 0.042, partial η² = 0.113).

The 1-Hz analysis at T2 produced a coherent set of adjusted findings concentrated in three regions of the spectrum. The strongest effects were in the low-beta range, where 22 Hz (partial η² = 0.286), 23 Hz (partial η² = 0.311), 24 Hz (partial η² = 0.300), and 25 Hz (partial η² = 0.262) all remained significant at p ≤ 0.001. Reductions were observed at 2 Hz (partial η² = 0.187), 3 Hz (partial η² = 0.312), and 4 Hz (partial η² = 0.221), and across 30–38 Hz with smaller effects (partial η² = 0.107–0.166). At T1, eight high-beta and low-gamma frequencies (28–34 and 37 Hz) showed significant reductions in delirium-prone patients (partial η² = 0.099–0.148).

The narrow-band and band-level findings responded differently to post-hoc demographic adjustment. Most narrow-band signals retained significance, with the 22–25 Hz cluster showing large effects (partial η² = 0.26–0.31), while the band-level theta difference did not. The adjusted narrow-band pattern was also spectrally coherent, with reductions concentrated in low-frequency bins and elevations clustered in the mid-beta range. By contrast, the band-level results were less consistent: T1 and T2 theta did not survive adjustment, and although T2 gamma remained significant, its small effect (partial η² = 0.113) and the variability expected with only seven delirium cases make it difficult to interpret cleanly. These observations suggest that, at least in this cohort, narrow-band features describe the delirium-related spectral signal more directly than averaged band power. Whether this advantage extends to other surgical populations and whether the 22–25 Hz cluster replicates under prospective adjustment for perioperative variables remain the key questions for future work.

### Diagnostic performance

**Fig 6:**
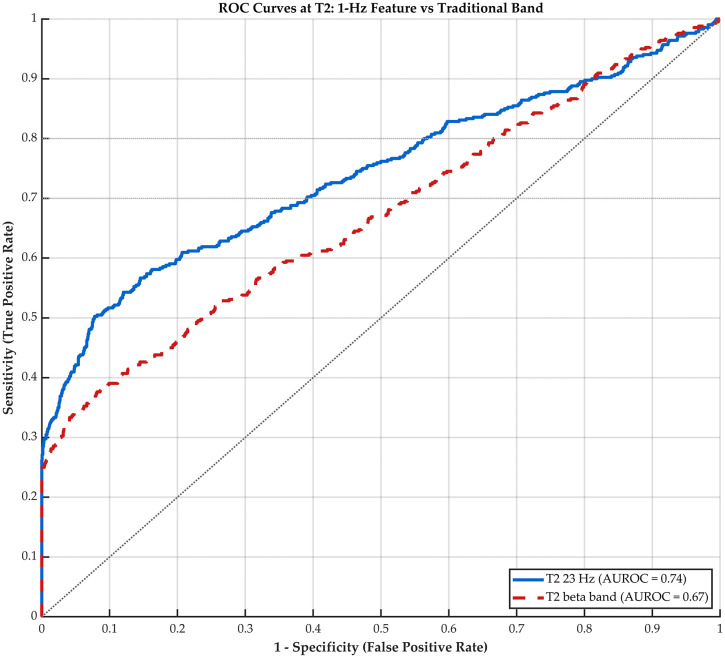
The receiver operating characteristic curve of the best-performing features at T2. The 1-Hz feature is shown in blue line, and traditional band feature is shown in red dash line. The area under the ROC curve (AUROC) is labelled in the legend for each feature.

We evaluated discriminative ability using receiver operating characteristic (ROC) analysis. Optimal thresholds were identified using Youden’s J statistic, selected to maximize the sum of sensitivity and specificity. Each spectral feature was evaluated independently based on its empirical distribution in delirium and non-delirium groups. This analysis is descriptive in nature, intended to characterize the discriminative potential of individual spectral features rather than to develop a predictive classifier. Fig 6 presents ROC curves for the best-performing 1-Hz frequency feature and the best traditional frequency band at T2.

Table 5 above compares diagnostic performance between the best traditional frequency band and the best 1-Hz frequency at timepoint T2. The 23 Hz frequency (AUROC = 0.74, 95% CI: 0.71–0.77) showed stronger discrimination than the best traditional band (beta, AUROC = 0.67, 95% CI: 0.64–0.70). The 1-Hz approach achieved higher sensitivity (50.2% compared to 37.6%) while maintaining comparable specificity (92.2% compared with 91.9%), resulting in a lower negative likelihood ratio (0.54 compared to 0.68). The 23 Hz power also remained significant after adjustment for age and sex (partial η² = 0.311), supporting interpretation as a candidate spectral feature rather than an artifact of demographic imbalance.

**Table 5:** Diagnostic performance at each timepoint: traditional Bands and 1-Hz resolution power at timepoint T2.

	Best Feature	AUC (95% CI)	p-value	Cut-off	Sensitivity (95% CI)	Specificity (95% CI)	PPV (95% CI)	NPV (95% CI)	LR+ (95% CI)	LR- (95% CI)
**T2**	Beta band	0.68 (0.63-0.74)	p < 0.01	35.04	39.3% (34.5-44.0)	88.8% (87.4-90.2)	43.2% (38.9-47.4)	87.0% (86.2-87.9)	3.49 (2.93-4.14)	0.68 (0.63-0.74)
	**23 Hz**	**0.74 (0.71-0.77)**	p < 0.01	2.27	58.6% (53.8-63.1)	83.5% (81.8-85.1)	43.5% (40.3-46.9)	90.2% (89.2-91.2)	3.54 (3.10-4.05)	0.50 (0.44-0.56)

* AUROC – area under the ROC curve; PPV – positive predictive value; NPV – negative predictive value; LR + – positive likelihood ratio; LR- – negative likelihood ratio.

Moreover, the moderate positive predictive values at T2 (50–58%) reflect the 14.9% delirium prevalence in our sample rather than poor classifier performance. This has practical implications for implementation. In high-prevalence settings such as ICU populations (30–50% delirium rates), PPV would improve substantially. More importantly, the high negative predictive values (~90%) suggest a specific clinical role: screening to rule out delirium. A negative result on a 23 Hz-based classifier would provide approximately 90% confidence that the patient does not have delirium, potentially reducing the burden of formal cognitive assessments in busy clinical environments. This application, using EEG as a triage tool rather than a definitive diagnostic, may represent the most practical implementation pathway.

Furthermore, to better contextualize these findings, we compared our diagnostic performance against EEG studies for postoperative delirium detection (shown in Table 6 below).

**Table 6:** Performance comparison with prior EEG delirium studies.

Study	Subject	EEG Method	Best Feature	AUCROC	Sensitivity	Specificity	Notes
Van der Kooi, et al 2015	Cardiac surgery ICU (n = 456)	Single-channel F8-Pz	Relative delta power	0.75	100%	96%	Multi-center
Numan, et al 2019	Mixed post-op (n = 161)	Single-channel Fp1-Fp2	Relative delta (1-min)	0.78	64%	83%	Multi-center
Fleischmann, et al 2019	Mixed ICU (n = 545)	Multi-channel qEEG	2 Hz specific	NR	100%	95-96%	19 channels
Kim, et al 2023	Elderly post-op (n = 257)	Prefrontal EEG	Median dominant freq	NR	NR	NR	8.65 vs 9.04 Hz
**Proposed study**							
1-Hz power at T2	Spinal Surgery (n = 47)	Single-channel Fp1	23 Hz	0.74	59%	84%	

* AUROC – area under the ROC curve; NR – not reported

The comparison with prior studies reveals population-dependent differences in optimal EEG biomarkers for delirium detection. In ICU patients, van der Kooi et al. achieved 100% sensitivity with 96% specificity using relative delta power from a single frontal channel [18]. Similarly, delta-based features performed well in Numan et al., who reported an AUC of 0.78 in general postoperative elderly patients [19], and in Fleischmann et al., who demonstrated AUC values of 0.82–0.86 in a heterogeneous hospital population [20]. These findings established delta power as the predominant EEG biomarker for delirium across diverse clinical settings. In our spinal surgery cohort, however, delta power provided minimal discriminative value, while beta-range frequencies (23 Hz) performed best at T2.

This divergence likely reflects differences in underlying pathophysiology. ICU patients and heterogeneous hospital cohorts frequently include individuals with sepsis, metabolic derangements, or multiorgan dysfunction. These conditions produce characteristic EEG slowing with increased delta activity [43,44]. Spinal surgery patients, by contrast, are typically medically stable but face distinct perioperative stressors. The prone position required for posterior spinal approaches elevates intrathoracic and intraabdominal pressure, impedes cerebral venous drainage, and can reduce middle cerebral artery blood velocity by approximately 10% [45,46]. Additionally, perioperative corticosteroids such as dexamethasone, routinely administered for pain management and nerve protection in spinal surgery [47,48], may modulate neuroinflammatory pathways in ways that differ from non-steroid-treated populations.

Furthermore, two recent spinal surgery studies support the notion of population-specific EEG signatures. Lee et al. [28] reported a 23.2% decrease in theta waves (p = 0.016) accompanied by elevated high-beta (19.3%, p = 0.003) and gamma activity (18.8%, p = 0.006) in delirium patients, a pattern similar to our findings. Kim et al. [49] demonstrated that lower preoperative median dominant frequency predicted postoperative delirium in 257 elderly spinal surgery patients (OR = 0.48, 95% CI: 0.27–0.85), with their prediction model achieving an AUC of 0.75. The convergence of theta reduction and beta/gamma elevation across these independent spinal surgery cohorts suggests a reproducible, population-specific spectral phenotype rather than methodological artifact.

Despite using a single-electrode montage, our diagnostic performance (AUC = 0.74) was comparable to multi-channel approaches. Van der Kooi et al. used a single frontal channel, Numan et al. employed limited-channel recordings, and Kim et al. used two prefrontal electrodes [18,19,49]. This convergence suggests that high-resolution spectral analysis may compensate for limited spatial coverage, supporting the feasibility of simplified EEG monitoring in routine clinical practice.

### Limitations and future direction

Several limitations should be considered when interpreting these findings. The most critical limitation is the small number of delirium cases. With only seven patients developing delirium out of 47 enrolled, effect estimates are inherently unstable, the group imbalance limits the reliability of between-group comparisons, and statistical power was insufficient to detect small effects reliably. A post-hoc power analysis confirms this concern. With the current sample, the study had approximately 80% power to detect only large between-group differences, corresponding to a correlation coefficient of about 0.52 or larger. Power to detect moderate effects in the range of |r| = 0.20 to 0.30 was below 40%. Even genuine moderate-sized differences may therefore have been overlooked in this dataset. We suggest that a future confirmatory study targeting a moderate effect of |r| = 0.30 at 80% power, with a similar group ratio, would require approximately 167 patients with at least 25 delirium events.

The statistical analyses carry additional constraints with the small sample size. FDR correction was applied across 45 frequency bins at each timepoint, but with a small sample size the possibility of false discoveries cannot be eliminated. Thus, the reported significant frequencies should be interpreted as candidate findings rather than validated markers. The ROC analysis is similarly constrained by the small sample size: with only seven delirium cases, AUROC estimates may be unstable, and the optimal thresholds derived using Youden’s J were identified and evaluated in the same dataset. Reported sensitivity and specificity values should therefore be interpreted with caution.

The scope of statistical adjustment was also limited. Age and sex were the only covariates included in the post-hoc analysis, and other perioperative variables could not be added without producing unstable estimates given only seven delirium cases. The reported findings therefore reflect adjustment for demographic factors alone, and replication in larger cohorts that can accommodate broader adjustment will be needed before stronger conclusions about these spectral features can be drawn.

Beyond sample size, the study population was limited to older adults undergoing elective spinal surgery at a single Japanese academic center, and findings may not generalize to other surgical populations, medical settings, or different ethnic groups. The atypical spectral pattern observed here may partly reflect population-specific factors rather than a universal delirium signature. Multi-site studies spanning different surgical types would help establish how broadly these findings apply. In addition, since EEG recordings were obtained during a brief mental counting task, future studies incorporating resting-state recordings would help disambiguate task-related from state-related theta changes.

Moreover, the single-electrode montage at Fp1 was selected for its practical advantages in a postoperative ward setting, where minimal setup time and compatibility with routine monitoring make single-channel EEG far more feasible than multi-channel systems. This practicality comes with a recognized trade-off, as spatial interpretation is limited, connectivity analyses cannot be performed, and spectral changes in posterior or temporal regions cannot be captured from a single prefrontal channel. Comparing single-channel and multi-channel recordings in the same patients would clarify whether the diagnostic signal identified here is prefrontal in origin or reflects broader cortical activity. Finally, delirium subtypes were not examined and may have distinct spectral profiles that group-level comparisons cannot capture.

## Conclusions

This pilot study suggests that 1-Hz spectral analysis can capture EEG features that traditional frequency bands do not, for postoperative delirium in spinal surgery patients. By analyzing narrow frequency bins, we found two notable spectral patterns: theta suppression at 3–7 Hz and elevated power at 23 Hz. These specific features were not present in broad-band analysis and were associated with higher diagnostic accuracy at delirium onset. The high negative predictive values make them most relevant for clinical screening, especially for ruling out delirium cases.

Our findings raise questions about treating traditional frequency bands as uniform physiological processes. When signals from different sub-frequencies are averaged together, the resulting band power may not reflect what is happening at finer scales. This may explain why broad-band theta and delta measures showed limited value in our cohort, even though these measures are the standard approach in most EEG delirium studies.

The small sample size limits the strength of these conclusions. Nevertheless, our results suggest that finer spectral resolution may offer a practical direction for improving EEG-based delirium detection. Multicenter studies with larger patient numbers will be needed to confirm whether these findings apply across different surgical populations.

## Supporting information

S1 Appendix Supporting tables for experimental conditions and statistical reports. Table A-D are presented in the appendix.
(DOCX)

S1 Fig Delta band (1–4 Hz) relative power distribution across timepoints. Boxplots compare non-delirium (blue) and delirium (red) groups. Sample sizes: T1 (non-delirium n = 35, delirium n = 7), T2 (control n = 32, delirium n = 7), T3 (non-delirium n = 26, delirium n = 2), and T4 (non-delirium n = 21, delirium n = 2). Effect sizes are reported as correlation (r). Significance levels after FDR correction: *p < 0.05, **p < 0.01, ***p < 0.001.
(TIF)

S2 Fig Theta band (4–8 Hz) relative power comparison across timepoints. Boxplots compare non-delirium (blue) and delirium (red) groups.
(TIF)

S3 Fig Alpha band (8–12 Hz) relative power comparison across timepoints. Boxplots compare non-delirium (blue) and delirium (red) groups.
(TIF)

S4 Fig Beta band (12–30 Hz) relative power comparison across timepoints. Boxplots compare non-delirium (blue) and delirium (red) groups.
(TIF)

S5 Fig Gamma band (30–45 Hz) relative power comparison across timepoints. Boxplots compare non-delirium (blue) and delirium (red) groups.
(TIF)

S6 Fig Complete heatmap of effect sizes for 1-Hz resolution spectral analysis across timepoints. Effect sizes are expressed as standardized effect size (r), with positive values (red) indicating higher power in the non-delirium group and negative values (blue) indicating higher power in the delirium group.
(TIF)
