# Supplementary material for: EEG spectral biomarkers of postoperative delirium in spinal surgery: A high-resolution analysis
Source: PLoS One. 2026 Jul 30;21(7):e0352607. doi: 10.1371/journal.pone.0352607 (PMC13423033; doi:10.1371/journal.pone.0352607)
Supplement: S1 Appendix — Table A-D are presented in the appendix. (DOCX) [file pone.0352607.s001.docx]

## S1 Appendix

**Table A**. Summary of assessments at different timepoints.


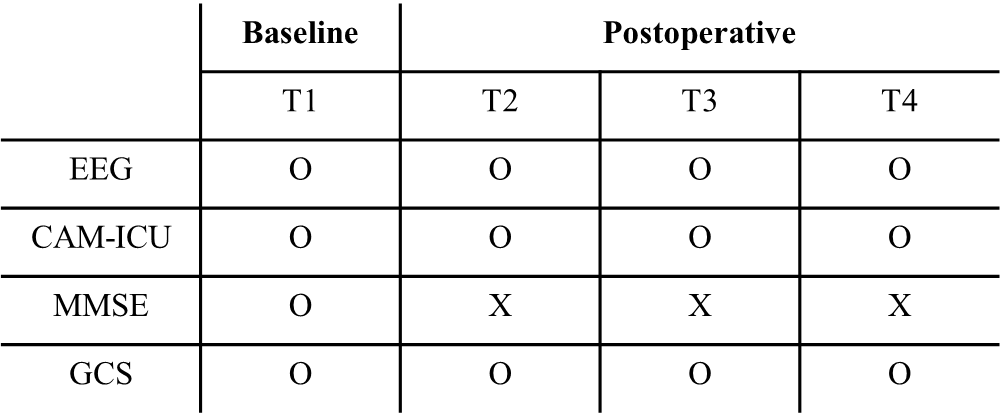


* “O” – test was conducted; “X” – test was not conducted.

**Table B**. Recording Information for Participants

| **Subject ID** | **EEG Recording Timepoint** | | | |
| --- | --- | --- | --- | --- |
|  | **T1** | **T2** | **T3** | **T4** |
| 1 | O | O | O | O |
| 2 | O | X | X | X |
| 3 | O | O | O | O |
| 4 | O | O | O | O |
| 5 | O | O | O | O |
| 6 | O | O | O | O |
| 7 | O | X | X | X |
| 8 | O | O | O | O |
| 9 | O | O | O | X |
| 10 | O | O | O | O |
| **11** | O | O | O | O |
| 12 | O | O | O | O |
| 13 | O | X | X | X |
| 14 | O | X | O | O |
| 15 | O | O | O | O |
| 16 | O | O | O | O |
| 17 | O | O | O | O |
| 18 | O | O | O | O |
| 19 | O | X | O | X |
| 20 | O | O | X | X |
| 21 | O | X | X | X |
| **22** | O | O | X | X |
| **23** | O | O | O | O |
| 24 | X | X | O | O |
| **25** | O | O | X | X |
| 26 | X | O | O | O |
| 27 | X | O | O | O |
| 28 | O | O | O | O |
| 29 | O | O | X | X |
| 30 | O | O | O | X |
| 31 | O | O | O | O |
| 32 | O | O | O | O |
| 33 | X | O | O | O |
| 34 | O | X | X | X |
| 35 | O | O | X | X |
| 36 | O | O | O | O |
| 37 | X | O | O | X |
| 38 | O | O | X | X |
| 39 | O | O | O | X |
| 40 | O | O | X | X |
| 41 | O | O | X | X |
| 42 | O | O | X | X |
| **43** | O | O | X | X |
| **44** | O | O | X | X |
| 45 | O | O | X | X |
| **46** | O | O | X | X |
| 47 | O | O | X | X |

* Bolded Subject ID indicates the subject was diagnosed with delirium.

** **O** – EEG data were collected; **X** – EEG recording was not available OR the experiment was not conducted.


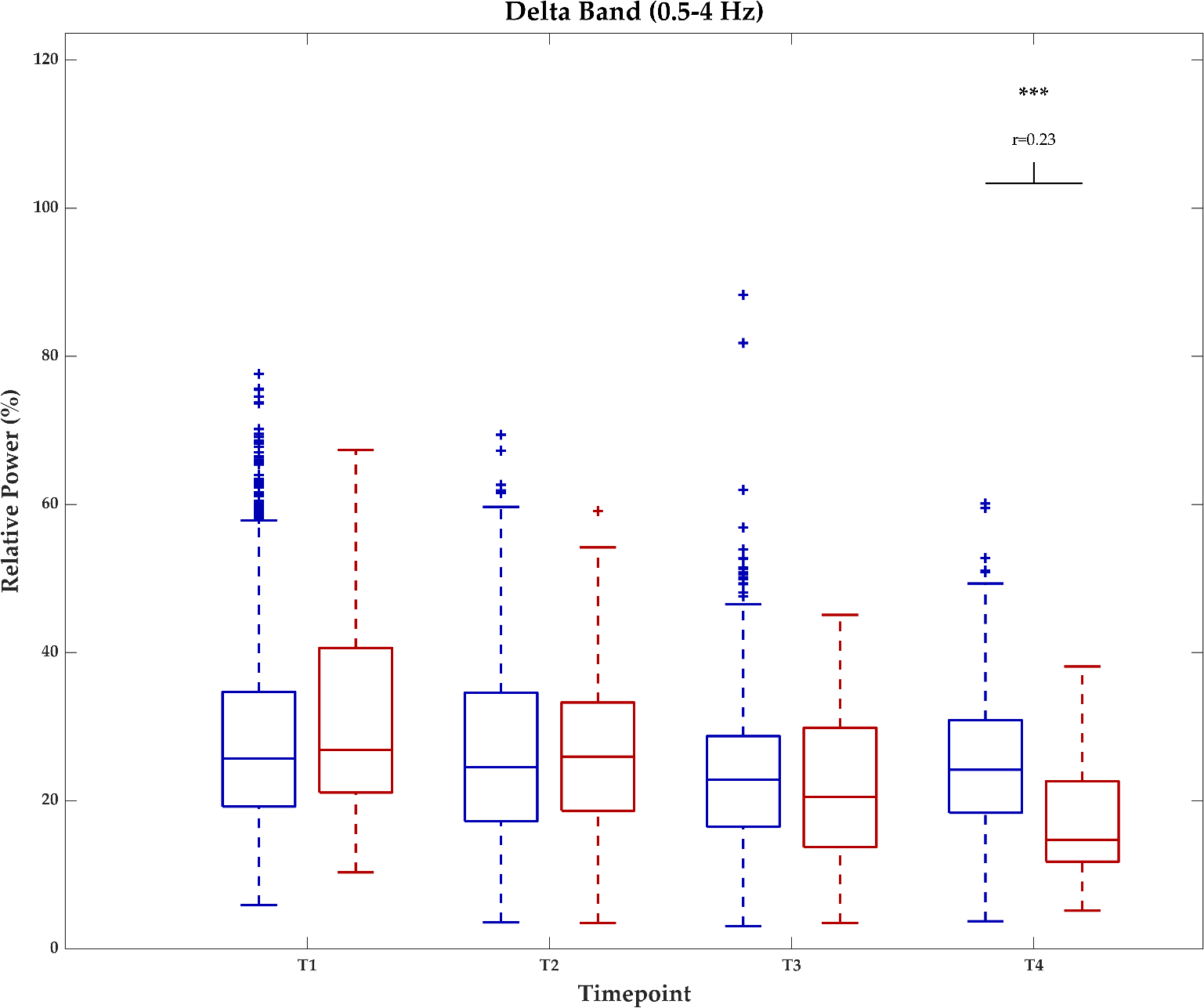


**S1 Fig**. Delta band (1-4 Hz) relative power distribution across timepoints. Boxplots compare non-delirium (blue) and delirium (red) groups. Sample sizes: T1 (non-delirium n = 35, delirium n = 7), T2 (control n = 32, delirium n = 7), T3 (non-delirium n = 26, delirium n = 2), and T4 (non-delirium n = 21, delirium n = 2). Effect sizes are reported as correlation (r). Significance levels after FDR correction: *p < 0.05, **p < 0.01, ***p < 0.001.


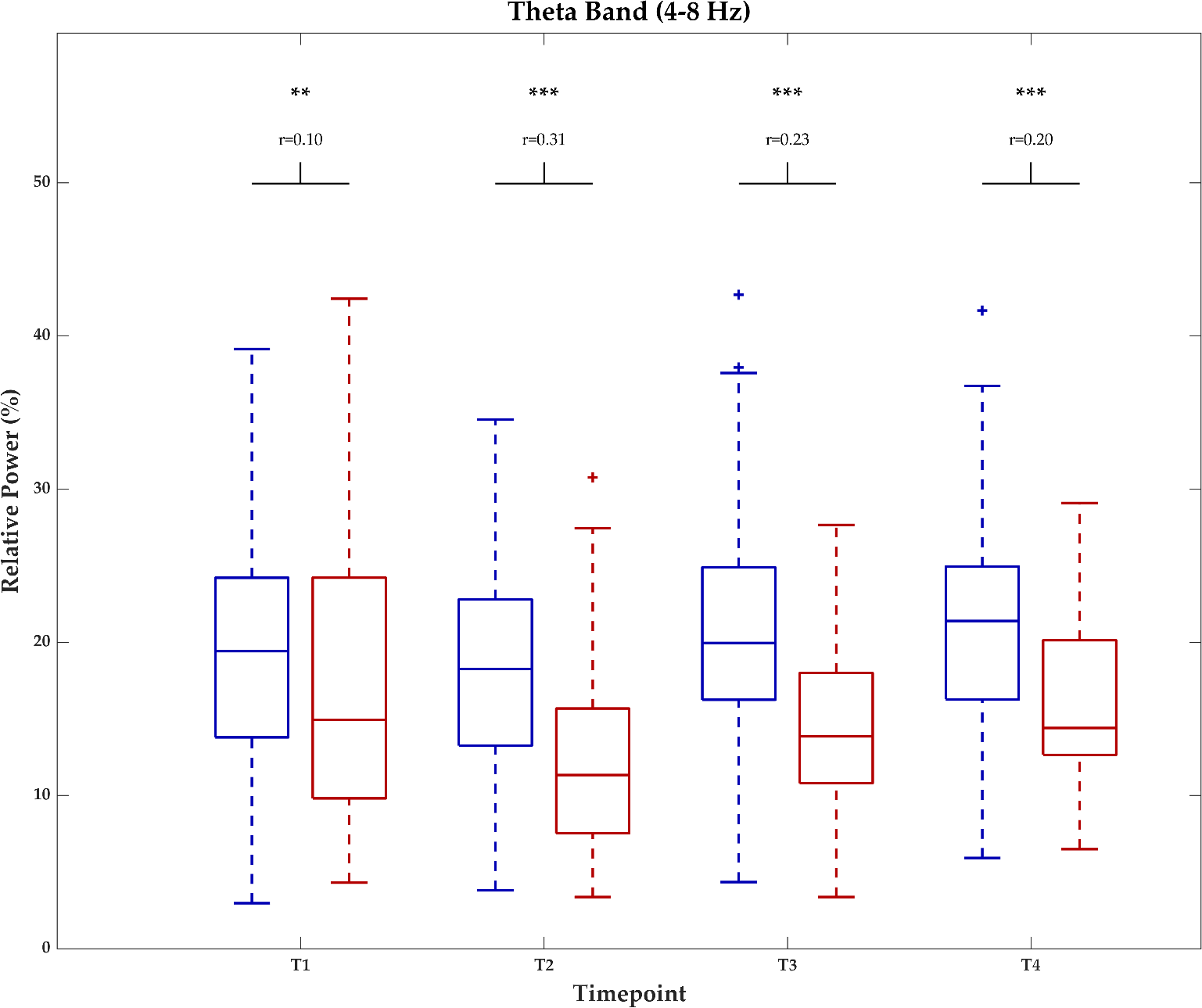


**S2 Fig**. Theta band (4-8 Hz) relative power comparison across timepoints. Boxplots compare non-delirium (blue) and delirium (red) groups. Sample sizes: T1 (non-delirium n = 35, delirium n = 7), T2 (control n = 32, delirium n = 7), T3 (non-delirium n = 26, delirium n = 2), and T4 (non-delirium n = 21, delirium n = 2). Effect sizes are reported as correlation (r). Significance levels after FDR correction: *p < 0.05, **p < 0.01, ***p < 0.001.


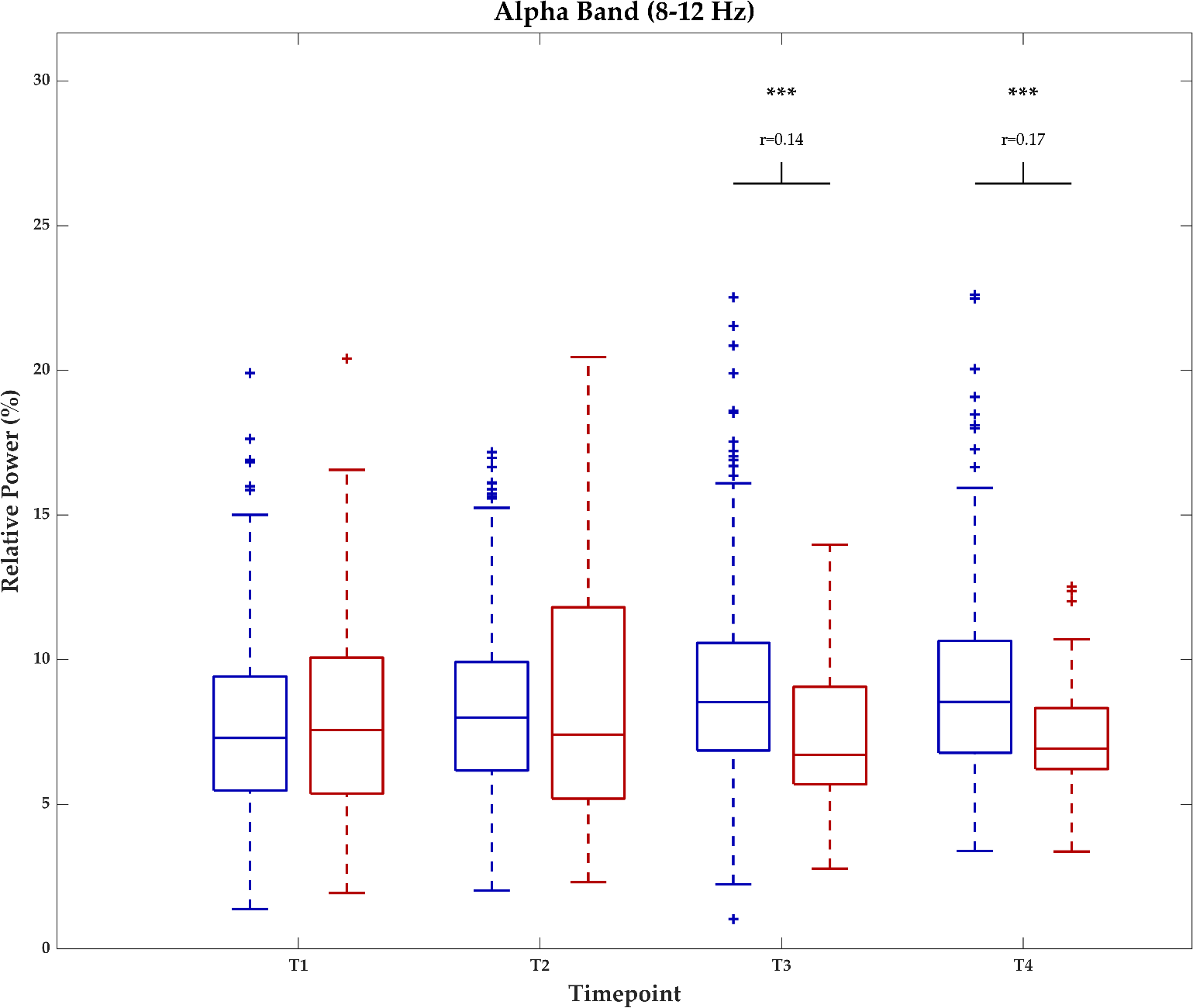


**S3 Fig**. Alpha band (8-12 Hz) relative power comparison across timepoints. Boxplots compare non-delirium (blue) and delirium (red) groups. Sample sizes: T1 (non-delirium n = 35, delirium n = 7), T2 (control n = 32, delirium n = 7), T3 (non-delirium n = 26, delirium n = 2), and T4 (non-delirium n = 21, delirium n = 2). Effect sizes are reported as correlation (r). Significance levels after FDR correction: *p < 0.05, **p < 0.01, ***p < 0.001.


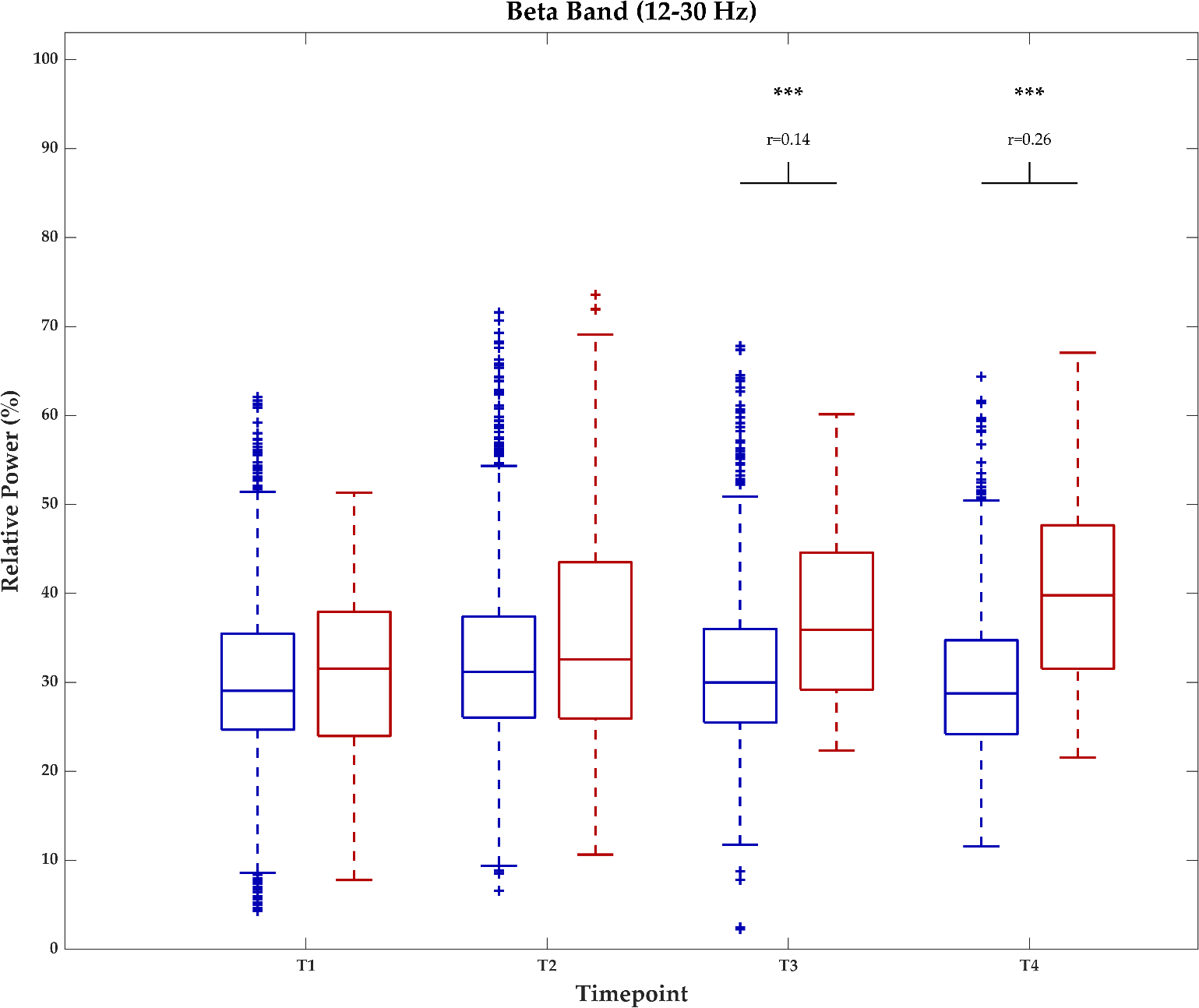


**S4 Fig.** Beta band (12-30 Hz) relative power comparison across timepoints. Boxplots compare non-delirium (blue) and delirium (red) groups. Sample sizes: T1 (non-delirium n = 35, delirium n = 7), T2 (control n = 32, delirium n = 7), T3 (non-delirium n = 26, delirium n = 2), and T4 (non-delirium n = 21, delirium n = 2). Effect sizes are reported as correlation (r). Significance levels after FDR correction: *p < 0.05, **p < 0.01, ***p < 0.001.


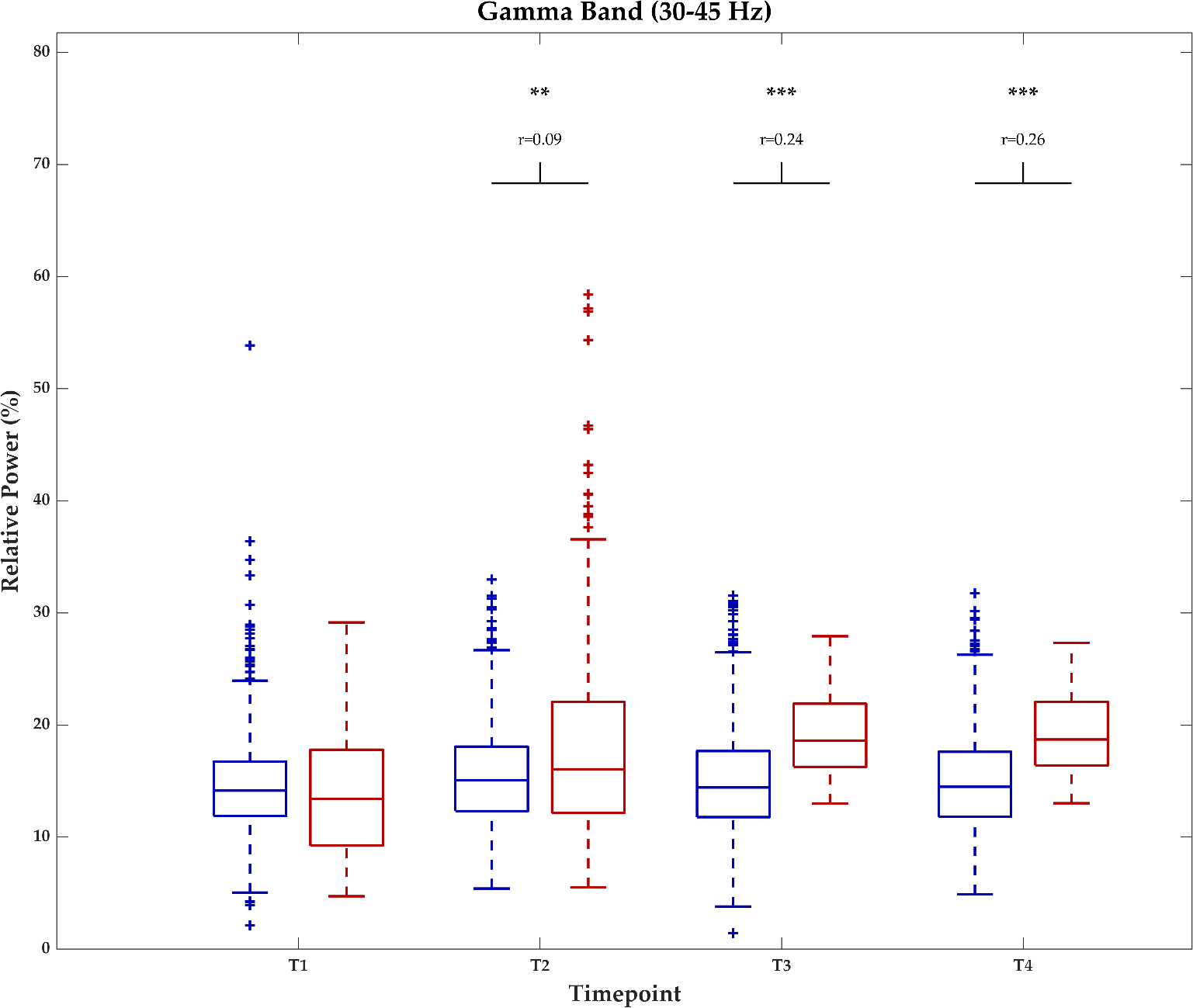

**S5 Fig**. Gamma band (30-45 Hz) relative power comparison across timepoints. Boxplots compare non-delirium (blue) and delirium (red) groups. Sample sizes: T1 (non-delirium n = 35, delirium n = 7), T2 (control n = 32, delirium n = 7), T3 (non-delirium n = 26, delirium n = 2), and T4 (non-delirium n = 21, delirium n = 2). Effect sizes are reported as correlation (r). Significance levels after FDR correction: *p < 0.05, **p < 0.01, ***p < 0.001.

**Table C**. Significant 1-Hz resolution EEG power comparisons with medium or above effect size ($\left| r \right| > 0.1$)

| **Timepoint** | **Freq  (Hz)** | **Band** | **Non-delirium mean (%) [95% CI]** | **Delirium mean (%) [95% CI]** | **z** | $\left\vert\boldsymbol{r} \right\vert$ | **Uncorrected p-value** | **FDR-corrected p-value** |
| --- | --- | --- | --- | --- | --- | --- | --- | --- |
| **T1** | 1 | delta | 3.81 [3.31, 4.34] | 7.12 [6.18, 8.22] | -8.92 | 0.26 | p<0.0001 | p<0.0001 |
|  | 3 | delta | 9.75 [9.49, 10.04] | 8.05 [7.51, 8.59] | 4.97 | 0.14 | p<0.0001 | p<0.0001 |
|  | 5 | theta | 4.57 [4.44, 4.71] | 4.11 [3.73, 4.51] | 3.92 | 0.11 | p<0.0001 | 0.0002 |
|  | 6 | theta | 3.40 [3.30, 3.53] | 3.03 [2.79, 3.32] | 3.61 | 0.10 | 0.0003 | 0.0005 |
|  | 7 | theta | 2.59 [2.52, 2.68] | 2.26 [2.10, 2.44] | 3.73 | 0.11 | 0.0002 | 0.0004 |
|  | 14 | beta | 1.24 [1.19, 1.30] | 1.53 [1.41, 1.68] | -4.72 | 0.14 | p<0.0001 | p<0.0001 |
|  | 15 | beta | 1.51 [1.43, 1.59] | 2.16 [1.93, 2.43] | -4.95 | 0.14 | p<0.0001 | p<0.0001 |
|  | 16 | beta | 1.97 [1.87, 2.08] | 3.42 [2.91, 4.00] | -3.65 | 0.10 | 0.0003 | 0.0004 |
|  | 21 | beta | 2.22 [2.19, 2.46] | 1.80 [1.59, 2.06] | 5.39 | 0.15 | p<0.0001 | p<0.0001 |
|  | 22 | beta | 1.65 [1.58, 1.73] | 1.38 [1.21, 1.58] | 5.74 | 0.16 | p<0.0001 | p<0.0001 |
|  | 23 | beta | 1.26 [1.22, 1.31] | 1.07 [0.97, 1.20] | 5.63 | 0.16 | p<0.0001 | p<0.0001 |
|  | 24 | beta | 1.04 [1.01, 1.07] | 0.91 [0.83, 1.01] | 4.99 | 0.14 | p<0.0001 | p<0.0001 |
|  | 25 | beta | 0.96 [0.94, 0.99] | 0.78 [0.72, 0.88] | 6.64 | 0.19 | p<0.0001 | p<0.0001 |
|  | 26 | beta | 0.89 [0.87, 0.92] | 0.71 [0.65 0.77] | 6.99 | 0.20 | p<0.0001 | p<0.0001 |
|  | 27 | beta | 0.81 [0.79, 0.84] | 0.64 [0.59, 0.69] | 7.01 | 0.20 | p<0.0001 | p<0.0001 |
|  | 28 | beta | 0.80 [0.77, 0.82] | 0.62 [0.57, 0.68] | 7.49 | 0.21 | p<0.0001 | p<0.0001 |
|  | 29 | beta | 0.78 [0.76, 0.81] | 0.63 [0.58, 0.68] | 6.56 | 0.19 | p<0.0001 | p<0.0001 |
|  | 30 | beta | 0.78 [0.76 0.80] | 0.64 [0.60, 0.69] | 5.91 | 0.17 | p<0.0001 | p<0.0001 |
|  | 31 | gamma | 0.78 [0.76, 0.80] | 0.66 [0.61, 0.71] | 4.79 | 0.14 | p<0.0001 | p<0.0001 |
|  | 32 | gamma | 0.80 [0.78, 0.82] | 0.70 [0.65, 0.76] | 4.55 | 0.13 | p<0.0001 | p<0.0001 |
|  | 33 | gamma | 0.83 [0.81 0.85] | 0.71 [0.66, 0.78] | 5.30 | 0.15 | p<0.0001 | p<0.0001 |
|  | 34 | gamma | 0.84 [0.82 0.87] | 0.72 [0.66, 0.79] | 5.23 | 0.15 | p<0.0001 | p<0.0001 |
|  | 35 | gamma | 0.87 [0.85, 0.90] | 0.78 [0.72, 0.85] | 4.20 | 0.12 | p<0.0001 | 0.0001 |
|  | 37 | gamma | 0.91 [0.90, 0.94] | 0.85 [0.77, 0.93] | 3.72 | 0.11 | 0.0002 | 0.0004 |
|  | 44 | gamma | 1.03 [0.98, 1.07] | 1.15 [1.07, 1.24] | -4.17 | 0.12 | p<0.0001 | 0.0001 |
|  | 45 | gamma | 1.08 [1.02, 1.14] | 1.32 [1.22, 1.43] | -6.51 | 0.19 | p<0.0001 | p<0.0001 |
| **T2** | 1 | delta | 2.51 [2.24, 2.83] | 6.06 [5.23, 6.86] | -9.18 | 0.27 | p<0.0001 | p<0.0001 |
|  | 2 | delta | 8.04 [7.64, 8.48] | 9.13 [8.41, 10.00] | -3.69 | 0.11 | 0.0002 | 0.0004 |
|  | 3 | delta | 9.35 [9.07, 9.63] | 6.47 [5.94, 7.02] | 8.94 | 0.27 | p<0.0001 | p<0.0001 |
|  | 4 | delta | 6.24 [6.03, 6.48] | 4.23 [3.84, 4.67] | 8.70 | 0.26 | p<0.0001 | p<0.0001 |
|  | 5 | theta | 4.14 [4.02, 4.27] | 2.82 [2.57, 3.09] | 8.96 | 0.27 | p<0.0001 | p<0.0001 |
|  | 6 | theta | 3.00 [2.91, 3.10] | 2.09 [1.95 2.25] | 8.61 | 0.26 | p<0.0001 | p<0.0001 |
|  | 7 | theta | 2.44 [2.37, 2.52] | 1.71 [1.60, 1.83] | 8.94 | 0.27 | p<0.0001 | p<0.0001 |
|  | 8 | theta | 2.05 [2.00, 2.12] | 1.66 [1.54, 1.78] | 6.04 | 0.18 | p<0.0001 | p<0.0001 |
|  | 12 | alpha | 1.32 [1.28, 1.37] | 1.90 [1.72, 2.09] | -4.56 | 0.14 | p<0.0001 | p<0.0001 |
|  | 13 | beta | 1.36 [1.31,1.41] | 2.14 [1.91, 2.36] | -5.38 | 0.16 | p<0.0001 | p<0.0001 |
|  | 14 | beta | 1.65 [1.57, 1.75] | 2.68 [2.42, 3.02] | -7.27 | 0.22 | p<0.0001 | p<0.0001 |
|  | 15 | beta | 2.19 [2.03, 2.38] | 3.43 [2.98, 4.03] | -8.72 | 0.26 | p<0.0001 | p<0.0001 |
|  | 16 | beta | 2.59 [2.43, 2.80] | 2.71 [2.46, 3.03] | -4.72 | 0.14 | p<0.0001 | p<0.0001 |
|  | 20 | beta | 2.50 [2.39, 2.63] | 2.61 [2.27, 3.05] | 3.71 | 0.11 | 0.0002 | 0.0004 |
|  | 21 | beta | 2.03 [1.95, 2.12] | 1.87 [1.68, 2.10] | 3.39 | 0.10 | 0.0007 | 0.0012 |
|  | 26 | beta | 1.04 [1.01, 1.07] | 0.93 [0.85, 1.01] | 4.26 | 0.13 | p<0.0001 | p<0.0001 |
|  | 27 | beta | 0.97 [0.94, 1.00] | 0.83 [0.77, 0.90] | 4.89 | 0.15 | p<0.0001 | p<0.0001 |
|  | 28 | beta | 0.95 [0.92, 0.98] | 0.78 [0.72, 0.84] | 5.79 | 0.17 | p<0.0001 | p<0.0001 |
|  | 29 | beta | 0.95 [0.93, 0.98] | 0.78 [0.72, 0.84] | 5.71 | 0.17 | p<0.0001 | p<0.0001 |
|  | 30 | beta | 0.94 [0.91, 0.97] | 0.77 [0.71, 0.83] | 5.63 | 0.17 | p<0.0001 | p<0.0001 |
|  | 31 | gamma | 0.93 [0.90, 0.96] | 0.76 [0.71, 0.82] | 5.55 | 0.16 | p<0.0001 | p<0.0001 |
|  | 32 | gamma | 0.93 [0.91, 0.96] | 0.82 [0.76, 0.87] | 3.90 | 0.12 | 0.0001 | 0.0002 |
|  | 41 | gamma | 0.97 [0.94, 1.00] | 1.34 [1.22, 1.49] | -3.43 | 0.10 | 0.0006 | 0.0010 |
|  | 42 | gamma | 0.94 [0.91, 0.97] | 1.37 [1.23, 1.54] | -4.31 | 0.13 | p<0.0001 | p<0.0001 |
|  | 43 | gamma | 0.95 [0.92, 0.98] | 1.59 [1.41, 1.80] | -5.34 | 0.16 | p<0.0001 | p<0.0001 |
|  | 44 | gamma | 0.97 [0.94, 1.00] | 1.73 [1.50, 1.98] | -4.40 | 0.13 | p<0.0001 | p<0.0001 |
|  | 45 | gamma | 0.97 [0.94., 1.00] | 2.29 [1.94, 2.74] | -5.96 | 0.18 | p<0.0001 | p<0.0001 |
| **T3** | 4 | delta | 6.50 [6.31, 6.70] | 5.19 [4.60, 5.79] | 3.45 | 0.12 | 0.0006 | 0.0012 |
|  | 5 | theta | 4.86 [4.69, 5.03] | 3.47 [3.08, 3.99] | 4.79 | 0.17 | p<0.0001 | p<0.0001 |
|  | 6 | theta | 3.77 [3.64, 3.93] | 2.53 [2.24, 2.92] | 5.67 | 0.20 | p<0.0001 | p<0.0001 |
|  | 7 | theta | 2.94 [2.85, 3.04] | 1.83 [1.65, 2.03] | 7.01 | 0.25 | p<0.0001 | p<0.0001 |
|  | 8 | theta | 2.36 [2.28, 2.44] | 1.71 [1.52, 1.96] | 4.78 | 0.17 | p<0.0001 | p<0.0001 |
|  | 9 | alpha | 2.01 [1.95, 2.08] | 1.47 [1.32. 1.63] | 4.65 | 0.17 | p<0.0001 | p<0.0001 |
|  | 14 | beta | 1.26 [1.21, 1.30] | 2.15 [1.84, 2.54] | -4.92 | 0.17 | p<0.0001 | p<0.0001 |
|  | 15 | beta | 1.48 [1.42, 1.54] | 3.26 [2.68, 4.14] | -6.65 | 0.24 | p<0.0001 | p<0.0001 |
|  | 16 | beta | 2.01 [1.91, 2.12] | 3.44 [2.85, 4.26] | -5.48 | 0.19 | p<0.0001 | p<0.0001 |
|  | 23 | beta | 1.35 [1.31, 1.40] | 1.90 [1.63, 2.27] | -3.19 | 0.11 | 0.0014 | 0.0027 |
|  | 24 | beta | 1.17 [1.13, 1.21] | 1.78 [1.58. 2.02] | -5.98 | 0.21 | p<0.0001 | p<0.0001 |
|  | 25 | beta | 1.11 [1.07, 1.15] | 1.76 [1.58, 1.97] | -7.04 | 0.25 | p<0.0001 | p<0.0001 |
|  | 26 | beta | 1.05 [1.01, 1.09] | 1.26 [1.15, 1.39] | -3.48 | 0.12 | 0.0005 | 0.0011 |
|  | 29 | beta | 0.90 [0.87, 0.93] | 1.17 [1.05, 1.31] | -4.36 | 0.15 | p<0.0001 | p<0.0001 |
|  | 33 | gamma | 0.94 [0.90, 0.97] | 1.13 [1.04, 1.24] | -3.85 | 0.14 | 0.0001 | 0.0003 |
|  | 34 | gamma | 0.93 [0.90, 0.96] | 1.15 [1.05, 1.27] | -3.90 | 0.14 | 0.0001 | 0.0002 |
|  | 35 | gamma | 0.98 [0.95, 1.01] | 1.24 [1.14, 1.37] | -4.70 | 0.17 | p<0.0001 | p<0.0001 |
|  | 37 | gamma | 0.98 [0.95, 1.01] | 1.34 [1.21, 1.52] | -4.62 | 0.16 | p<0.0001 | p<0.0001 |
|  | 38 | gamma | 0.97 [0.94, 1.01] | 1.42 [1.24, 1.65] | -4.59 | 0.16 | p<0.0001 | p<0.0001 |
|  | 39 | gamma | 0.96 [0.93, 1.00] | 1.47 [1.29, 1.70] | -5.03 | 0.18 | p<0.0001 | p<0.0001 |
|  | 40 | gamma | 0.96 [0.93, 1.00] | 1.35 [1.19, 1.55] | -4.79 | 0.17 | p<0.0001 | p<0.0001 |
|  | 41 | gamma | 0.95 [0.92, 0.99] | 1.43 [1.24, 1.71] | -4.67 | 0.17 | p<0.0001 | p<0.0001 |
|  | 42 | gamma | 0.93 [0.89, 0.96] | 1.15 [1.04, 1.32] | -3.44 | 0.12 | 0.0006 | 0.0012 |
|  | 43 | gamma | 0.92 [0.89, 0.96] | 1.12 [0.99, 1.26] | -3.26 | 0.12 | 0.0011 | 0.0022 |
| **T4** | 1 | delta | 1.08 [1.02, 1.14] | 0.61 [0.52, 0.73] | 5.91 | 0.23 | p<0.0001 | p<0.0001 |
|  | 2 | delta | 7.09 [6.72, 7.50] | 4.42 [3.70, 5.37] | 4.93 | 0.19 | p<0.0001 | p<0.0001 |
|  | 3 | delta | 10.22 [9.85, 10.60] | 6.87 [5.96, 8.09] | 5.59 | 0.22 | p<0.0001 | p<0.0001 |
|  | 4 | delta | 6.47 [6.27, 6.68] | 5.18 [4.52, 5.81] | 3.89 | 0.15 | 0.0001 | 0.0002 |
|  | 5 | theta | 4.90 [4.73, 5.07] | 3.89 [3.48, 4.32] | 3.57 | 0.18 | 0.0004 | 0.0006 |
|  | 6 | theta | 3.86 [3.72, 4.01] | 2.97 [2.62, 3.41] | 4.01 | 0.15 | 0.0001 | 0.0001 |
|  | 7 | theta | 3.12 [3.01, 3.22] | 2.39 [2.14, 2.69] | 4.13 | 0.16 | p<0.0001 | 0.0001 |
|  | 8 | theta | 2.46 [2.37, 2.55] | 1.89 [1.72, 2.12] | 3.73 | 0.14 | 0.0002 | 0.0004 |
|  | 9 | alpha | 2.04 [1.97, 2.12] | 1.64 [1.51, 1.80] | 3.25 | 0.13 | 0.0012 | 0.0018 |
|  | 11 | alpha | 1.45 [1.41, 1.51] | 1.15 [1.04, 1.27] | 3.60 | 0.14 | 0.0003 | 0.0006 |
|  | 14 | beta | 1.20 [1.16, 1.25] | 2.29 [1.97, 2.68] | -6.62 | 0.26 | p<0.0001 | p<0.0001 |
|  | 15 | beta | 1.41 [1.35, 1.48] | 4.94 [4.20, 5.80] | -10.11 | 0.39 | p<0.0001 | p<0.0001 |
|  | 16 | beta | 1.86 [1.76, 1.97] | 6.57 [5.71, 7.48] | -10.98 | 0.42 | p<0.0001 | p<0.0001 |
|  | 17 | beta | 2.75 [2.54, 3.01] | 4.42 [3.99, 4.89] | -7.79 | 0.30 | p<0.0001 | p<0.0001 |
|  | 22 | beta | 1.86 [1.75, 2.00] | 1.15 [1.06, 1.26] | 4.12 | 0.16 | p<0.0001 | 0.0001 |
|  | 26 | beta | 0.97 [0.93, 1.01] | 1.09 [0.99, 1.19] | -2.60 | 0.10 | 0.0093 | 0.0131 |
|  | 27 | beta | 0.88 [0.85, 0.91] | 1.13 [1.02, 1.28] | -4.37 | 0.17 | p<0.0001 | p<0.0001 |
|  | 28 | beta | 0.86 [0.83, 0.89] | 1.14 [1.04, 1.25] | -5.47 | 0.21 | p<0.0001 | p<0.0001 |
|  | 29 | beta | 0.82 [0.79, 0.86] | 1.23 [1.14, 1.35] | -7.10 | 0.27 | p<0.0001 | p<0.0001 |
|  | 30 | beta | 0.81 [0.79, 0.84] | 1.33 [1.22, 1.44] | -8.49 | 0.33 | p<0.0001 | p<0.0001 |
|  | 31 | gamma | 0.84 [0.81, 0.88] | 1.31 [1.20, 1.43] | -7.45 | 0.29 | p<0.0001 | p<0.0001 |
|  | 32 | gamma | 0.87 [0.84, 0.90] | 1.31 [1.19, 1.46] | -6.82 | 0.26 | p<0.0001 | p<0.0001 |
|  | 33 | gamma | 0.87 [0.84, 0.90] | 1.27 [1.17, 1.37] | -6.93 | 0.27 | p<0.0001 | p<0.0001 |
|  | 34 | gamma | 0.90 [0.87, 0.93] | 1.17 [1.07, 1.30] | -4.61 | 0.18 | p<0.0001 | p<0.0001 |
|  | 35 | gamma | 0.95 [0.92, 0.98] | 1.15 [1.05, 1.27] | -3.57 | 0.14 | 0.0004 | 0.0006 |
|  | 36 | gamma | 0.94 [0.91, 0.97] | 1.17 [1.06, 1.30] | -3.72 | 0.14 | 0.0002 | 0.0004 |
|  | 37 | gamma | 0.95 [0.92, 0.99] | 1.18 [1.08, 1.28] | -4.28 | 0.17 | p<0.0001 | p<0.0001 |
|  | 38 | gamma | 0.98 [0.94, 1.02] | 1.24 [1.13, 1.36] | -4.69 | 0.18 | p<0.0001 | p<0.0001 |
|  | 39 | gamma | 1.00 [0.96, 1.04] | 1.22 [1.11, 1.33] | -4.46 | 0.17 | p<0.0001 | p<0.0001 |
|  | 42 | gamma | 0.99 [0.95, 1.04] | 1.12 [1.01, 1.23] | -2.81 | 0.11 | 0.0050 | 0.0073 |
|  | 43 | gamma | 0.98 [0.94, 1.02] | 1.10 [1.01, 1.20] | -3.10 | 0.12 | 0.0019 | 0.0029 |
|  | 44 | gamma | 0.96 [0.92, 1.00] | 1.13 [1.03, 1.26] | -3.51 | 0.14 | 0.0004 | 0.0007 |

* z: z-statistics from Wilcoxon rank-sum test; $\left| r \right|$: absolute correlation

**Table D. Absolute spectral power comparisons between non-delirium and delirium groups across timepoints.**

| **Timepoint** | **Band** | **Non-delirium mean (μV²) [95% CI]** | **Delirium mean (μV²)  [95% CI]** | **z** | **r** | **FDR-corrected p-value** |
| --- | --- | --- | --- | --- | --- | --- |
| **T1** | delta | 0.2071 [0.2007, 0.2133] | 0.2372 [0.2228, 0.2513] | -4.14 | 0.12 | 0.0002 |
|  | theta | 0.1225 [0.1188, 0.1258] | 0.1144 [0.1067, 0.1223] | 2.15 | 0.06 | 0.0393 |
|  | alpha | 0.0542 [0.0528, 0.0557] | 0.0613 [0.0576, 0.0654] | -3.08 | 0.09 | 0.0052 |
|  | beta | 0.2160 [0.2112, 0.2210] | 0.2335 [0.2209, 0.2456] | -2.59 | 0.07 | 0.0162 |
|  | gamma | 0.1046 [0.1025, 0.1066] | 0.1076 [0.1018, 0.1139] | 0.34 | 0.01 | 0.7368 |
| **T2** | delta | 0.1954 [0.1893, 0.2019] | 0.1859 [0.1732, 0.1990] | 0.80 | 0.02 | 0.4229 |
|  | theta | 0.1174 [0.1140, 0.1207] | 0.0776 [0.0709, 0.0848] | 10.37 | 0.31 | <0.0001 |
|  | alpha | 0.0601 [0.0587, 0.0616] | 0.0610 [0.0563, 0.0658] | 0.88 | 0.03 | 0.6316 |
|  | beta | 0.2394 [0.2339, 0.2456] | 0.2457 [0.2309, 0.2604] | -1.19 | 0.04 | 0.5861 |
|  | gamma | 0.1137 [0.1114, 0.1162] | 0.1191 [0.1133, 0.1258] | -0.87 | 0.03 | 0.4793 |
| **T3** | delta | 0.1722 [0.1663, 0.1783] | 0.1576 [0.1373, 0.1798] | 1.15 | 0.04 | 0.2500 |
|  | theta | 0.1305 [0.1268, 0.1344] | 0.0932 [0.0837, 0.1054] | 5.29 | 0.19 | <0.0001 |
|  | alpha | 0.0628 [0.0612, 0.0645] | 0.0512 [0.0470, 0.0559] | 3.97 | 0.14 | 0.0001 |
|  | beta | 0.2325 [0.2261, 0.2387] | 0.2533 [0.2413, 0.2650] | -2.92 | 0.10 | 0.0044 |
|  | gamma | 0.1097 [0.1069, 0.1128] | 0.1359 [0.1279, 0.1450] | -5.20 | 0.18 | <0.0001 |
| **T4** | delta | 0.1761 [0.1705, 0.1819] | 0.1214 [0.1054, 0.1396] | 5.47 | 0.21 | <0.0001 |
|  | theta | 0.1300 [0.1263, 0.1336] | 0.1012 [0.0906, 0.1134] | 4.37 | 0.17 | <0.0001 |
|  | alpha | 0.0623 [0.0607, 0.0641] | 0.0496 [0.0466, 0.0533] | 4.78 | 0.18 | <0.0001 |
|  | beta | 0.2189 [0.2124, 0.2259] | 0.2731 [0.2568, 0.2921] | -5.28 | 0.20 | <0.0001 |
|  | gamma | 0.1076 [0.1046, 0.1109] | 0.1295 [0.1229, 0.1379] | -4.78 | 0.18 | <0.0001 |

*z: z-statistics from Wilcoxon rank-sum test; r: absolute correlation. FDR correction applied within each timepoint across the 5 bands. Bootstrap 95% confidence intervals computed from 2,000 resamples. Frequency bands: delta (0.5–4 Hz), theta (4–7 Hz), alpha (8–12 Hz), beta (12–30 Hz), gamma (30–45 Hz).*


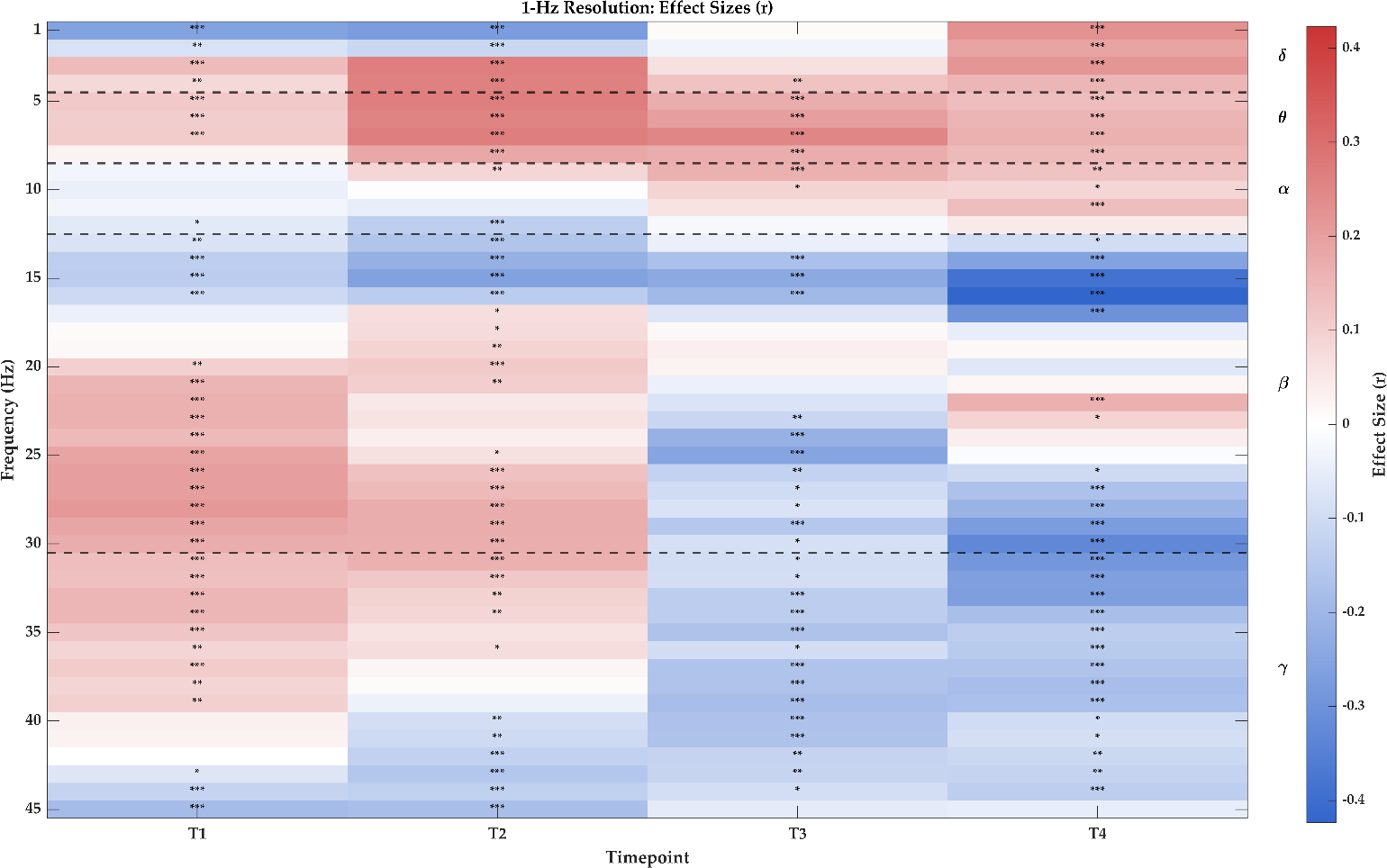


**S6 Fig**. Complete heatmap of effect sizes for 1-Hz resolution spectral analysis across timepoints. Effect sizes are expressed as Rosenthal's r, with positive values (red) indicating higher power in the non-delirium group and negative values (blue) indicating higher power in the delirium group. Rows represent individual frequencies (1-45 Hz) and columns represent timepoints (T1-T4). Significant differences after FDR correction are marked with: *p < 0.05, **p < 0.01, ***p < 0.001.
