# Supplementary material for: EEG spectral biomarkers of postoperative delirium in spinal surgery: A high-resolution analysis
Source: PLoS One. 2026 Jul 30;21(7):e0352607. doi: 10.1371/journal.pone.0352607 (PMC13423033; doi:10.1371/journal.pone.0352607)
Supplement: Attachment — Submitted filename: Response to Reviewers.docx [file pone.0352607.s011.docx]

**Response to Reviewers**

*Manuscript ID: PONE-D-25-68816*

We sincerely thank both reviewers for their constructive feedback. The comments led us to rework the framing, the statistical reporting, and the interpretation of our findings. The study is now presented as an exploratory pilot investigation, the diagnostic claims have been moderated, and the limitations are stated more directly. Reviewer comments are reproduced in italics below, followed by our response.

**Reviewer #1**

**Major Concerns**

**1. Sample size and statistical power**

**Comment:**

*The study includes 47 patients, of whom only 7 developed delirium. All delirium cases occurred at T2, and only 2 delirium patients contributed data at T3 and T4. The manuscript must clearly state that this is an exploratory pilot study. T3 and T4 findings should be described as preliminary observations only. Any physiological interpretation based on two patients must be toned down or moved to supplementary material. A brief discussion of statistical power would strengthen the paper.*

**Response:**

We agree. The Abstract, Introduction, and Conclusion now describe the work as a pilot study, and the small sample is treated as the primary constraint throughout.

T3 and T4 are now reported as descriptive observations from two patients. Physiological claims that depending on those two patients have been removed, and the diagnostic performance evaluation is restricted to T2 (Fig 5 and Table 6). The within-subject T1–T4 trajectory is retained because both patients with available recordings showed the same direction of change, but it is clearly labeled as such.

On power, a post-hoc power analysis has been added to the Limitations section. With the current sample, the study had approximately 80% power to detect only large between-group differences (correlation coefficient of about 0.52 or larger), and power to detect moderate effects in the range of |r| = 0.20 to 0.30 was below 40%. We also estimate that a future confirmatory study targeting a moderate effect of |r| = 0.30 at 80% power, with a similar group ratio, would require approximately 167 patients with at least 25 delirium events.

**2. Multiple comparisons and frequency-level interpretation**

**Comment:**

*At each timepoint, 45 frequency bins were tested. Even with FDR correction, the large number of positive bins raises concern for type I error. The authors should explicitly state the total number of tests performed, clarify how FDR correction was applied at each timepoint, focus interpretation on consistent effect size patterns rather than number of significant bins, and avoid attributing physiological meaning to every significant frequency.*

**Response:**

The Methods section now explicitly states the total test count: 180 tests for the 1-Hz analysis (45 frequencies × 4 timepoints) and 20 tests for the band analysis (5 bands × 4 timepoints). FDR correction was applied within each timepoint, separately for the band-level and the 1-Hz analyses, since each timepoint represents a different clinical state.

The Results and Discussion section focuses on three coherent patterns at T2: a 1 Hz elevation, a 3–7 Hz reduction, and a low-beta elevation around 14–16 Hz. Fig 4 shows only frequencies meeting both FDR significance and |r| ≥ 0.20, which makes the consistent patterns visible without the visual clutter of small-effect bins. The full 45-frequency heatmap is moved to Fig S6.

**3. Diagnostic performance and risk of optimism bias**

**Comment:**

*ROC curves and optimal thresholds were derived and tested in the same dataset. With only 7 delirium cases, AUROC values such as 0.74 may be optimistic. The manuscript should clearly state: no cross validation was performed, no external validation cohort was used, thresholds were optimized on the same sample, diagnostic metrics are hypothesis generating.*

**Response:**

All four points are now stated in the Limitations section. The AUROC is described as moderate rather than strong, the 95% CI is reported alongside the point estimate (AUROC = 0.74, 95% CI 0.71–0.77), and the threshold was identified and evaluated in the same dataset. The 23 Hz feature is described as a candidate for future validation, not as a classifier ready for clinical use.

We considered leave-one-out cross-validation but did not include it in the revised manuscript. With seven cases, leave-one-out at the patient level removes one of seven events at each fold, and the cross-validated estimate carries the same instability as the in-sample one. External validation in a larger cohort is identified as the appropriate next step.

**4. Interpretation of theta suppression**

**Comment:**

*Most prior delirium EEG studies report increased theta and delta power. This study reports reduced theta in delirium patients. The authors should discuss: whether relative power normalization influenced findings, possible impact of the mental counting task on theta activity, whether absolute power analyses were performed, alternative explanations beyond population specificity.*

**Response:**

This part of the Discussion has been rewritten. The reduction in theta is no longer presented as a clean spinal-surgery phenotype, and two alternative explanations are discussed alongside it.

First, the mental counting task. Frontal midline theta increases during mental arithmetic, so reduced engagement with the task in delirium patients could itself produce a theta reduction unrelated to resting-state activity.

Second, the relative versus absolute power question. We ran the absolute power analysis to check. Both relative and absolute power show theta reduction at T2 in the same direction (Table S4), so the finding is less likely to be an artifact generated from normalization. This is noted in the Discussion.

**5. Confounding variables**

**Comment:**

*The study does not adjust for perioperative variables such as duration of surgery, blood loss, steroid exposure, opioid dose, type of procedure, baseline cognitive reserve. Even a simple logistic regression adjusting for age and sex would strengthen credibility.*

**Response:**

A post-hoc ANCOVA with age and sex as covariates has been added, applied to all features that reached FDR significance in the primary analysis. The results are reported in the Results section.

The outcome was informative. At the band level, the T1 and T2 theta differences did not survive adjustment, while T2 gamma did. At the 1-Hz level, the picture was different. The 22–25 Hz cluster at T2 remained significant after adjustment, with partial η² = 0.26–0.31 across these four bins, and the 23 Hz feature held up at partial η² = 0.311. Reductions in the low-frequency bins (2–4 Hz) also persisted.

The adjustment was not extended to the perioperative variable list. With seven events, even the two-covariate model is at the edge of acceptable events-per-variable, and adding more covariates produces unstable estimates. This is stated in the Limitations, and broader adjustment is identified as a task for the validation cohort.

**Moderate Concerns**

**6. Definition of delirium group**

**Comment:**

*Delirium is defined as CAM positive at any postoperative timepoint. In practice, all cases occurred at T2. This should be clarified clearly in the Methods section, not only in Results.*

**Response:**

We appreciate this comment. The Methods section now clarifies this as: delirium was defined as CAM-positive at any postoperative assessment, but in this cohort all seven cases were CAM-positive at T2 only, with no new incident cases at T3 or T4.

**7. Baseline predictive claims**

**Comment:**

*The manuscript reports preoperative spectral differences and implies predictive potential. With only 7 future delirium cases and no predictive model, this should be framed as preliminary signal exploration rather than prediction.*

**Response:**

The predictive framing has been removed from the T1 results. The context now describes T1 differences as preliminary observations of baseline spectral patterns in patients who later did and did not develop delirium. We do not claim a predictive model for the T1 findings.

**8. Data availability**

**Comment:**

*The data availability statement indicates restrictions. PLOS One requires strong justification for restricted data. The authors should clarify whether deidentified spectral data can be shared.*

**Response:**

The Data Availability statement has been revised. Raw EEG recordings remain restricted under the original IRB-approved consent at Keio University Hospital, but readers can request access through the Keio School of Medicine Ethics Committee, subject to a data use agreement and local approval. Please contact the administrative officer Dr. Sumitaka Hase, via [shase@adst.keio.ac.jp](mailto:shase@adst.keio.ac.jp).

**9. Single-electrode limitation**

**Comment:**

*Using Fp1 only limits spatial interpretation. The manuscript should explicitly state that connectivity and regional dynamics cannot be assessed and that spectral resolution does not replace spatial information.*

**Response:**

This is now stated in the Limitations. Single-channel Fp1 recordings cannot support connectivity analyses, source localization, or any inference about posterior or temporal regions, and spectral resolution does not substitute for spatial information. Fp1 was selected because single-electrode recording is realistic for routine ward use, and the trade-off is acknowledged as such.

**Minor Issues**

**10. Language and clarity**

**Comment:**

*Some sections contain repeated phrases or long speculative paragraphs. The discussion would benefit from tightening and removal of redundant sentences.*

**Response:**

Thank you for your comment. We have revised the document accordingly.

**11. Overstatement of conclusions**

**Comment:**

*Phrases such as identifying discrete EEG biomarkers or outperforming conventional bands should be softened. Suggested wording: candidate frequency-specific features in this dataset; conventional bands did not detect certain frequency-specific differences observed in this sample.*

**Response:**

We appreciate the suggestion and we realize some of our wording might be misleading. The revision was made to those phrases.

**12. Figure interpretation**

**Comment:**

*The 1 Hz heatmap is visually dense. Consider adding a simplified summary figure highlighting key frequencies with medium or larger effects.*

**Response:**

We agreed. Fig 4 has been redrawn to show only the 20 frequencies meeting both FDR significance and |r| ≥ 0.20 across timepoints, grouped by conventional band. The full 45-frequency heatmap is retained as Fig S6. The simplified figure which highlights the principal patterns (3–7 Hz reduction, 14–16 Hz elevation, 23 Hz peak at T2) should be easier to read now.

**Reviewer #2**

Reviewer 2's concerns overlap closely with those raised by Reviewer 1. Where the underlying issue is the same, we cross-reference rather than repeat. We appreciate your time for reading our response.

**Main Problems**

**1. Sample size, number of events, and statistical stability**

**Comment:**

*With a limited sample size (N = 47), only 7 cases (14.9%) were diagnosed with delirium. Only 2 cases of delirium remained in the later stages (T3 and T4). Recommendation: (1) explicitly acknowledge limited and unstable statistical power throughout. (2) describe T3 and T4 findings as exploratory only. (3) restrict main conclusions to T2.*

**Response:**

Implemented as described in our response to **Reviewer 1, Major Concern 1**. The framing is now exploratory throughout, T3 and T4 are descriptive observations from two patients only, and the diagnostic performance evaluation is restricted to T2. A post-hoc power analysis has been added showing ~80% power only for large effects (|r| ≥ 0.52) and below 40% power for moderate effects (|r| = 0.20–0.30).

**2. Confounding factors and limitations of adjustment**

**Comment:**

*The sex distribution was unbalanced (71.4% male in the delirium group and 52.5% in the control group). Age and baseline MMSE are also predictors of delirium. Recommendation: consider adjusted analyses, but treat them as exploratory given seven events. Acknowledge the lack of robust adjustment as a limitation.*

**Response:**

Addressed as described in our response to **Reviewer 1, Major Concern 5**. A post-hoc ANCOVA with age and sex was added, applied only to features that reached FDR significance in the primary analysis. The 22–25 Hz cluster at T2, including the 23 Hz feature, survived adjustment with partial η² = 0.26–0.31; band-level theta did not. The Limitations section notes that broader perioperative adjustment was not feasible with seven events.

**3. Interpretation of diagnostic performance**

**Comment:**

*AUROC of 0.74 is moderate rather than strong. With small N, the AUROC and the Youden cutoff are susceptible to overfitting. Recommendation: temper claims about superiority, report AUROC confidence intervals, and consider internal validation.*

**Response:**

Comparative language has been softened, and 95% CIs are reported for AUROC, sensitivity, specificity, PPV, NPV, and likelihood ratios in Table 6. The in-sample, hypothesis-generating nature of the metrics is stated in the Limitations. Leave-one-out CV was considered but not included, as explained in our response to **Reviewer 1, Major Concern 3**.

**4. Data availability**

**Comment:**

*The current statement does not comply with PLOS ONE's data availability standards.*

**Response:**

Addressed as described in our response to **Reviewer 1, Moderate Concern 8**. Raw recordings and processed data remain restricted under the original consent but can be requested through the institutional ethics committee. Please contact the administrative officer Dr. Sumitaka Hase, via [shase@adst.keio.ac.jp](mailto:shase@adst.keio.ac.jp).

**Minor Revisions**

- **Comment:**

*The ROC curves for T3 and T4 should be removed because the analysis based on N=2 is statistically meaningless.*

**Response:**

Thank you for your suggestion. Fig 5 now shows only the T2 ROC curves, comparing the best 1-Hz feature (23 Hz) with the best traditional band (beta). The figure caption and the corresponding Results text have been updated.

- **Comment:**

*Replace assertive terms such as "proof" with more appropriate language.*

**Response:**

Thank you for your suggestion, and we described in our response to **Reviewer 1, Minor Issue 11**. Assertive terms have been replaced with hedged equivalents, and “biomarkers” is now “candidate features.”

- **Comment:**

*The manuscript should discuss the limitations more prominently, especially small sample size, overfitting, and the risk of false positives.*

**Response:**

The Limitations section has been fully rewritten. It now opens with the small number of events as the primary constraint, including the post-hoc power analysis described above, addresses overfitting from in-sample threshold selection using Youden's J, and discusses false-positive risk despite FDR correction. The low PPV at T2 is contextualized as a consequence of the 14.9% prevalence rather than poor classifier performance, and the practical role of these features is described as rule-out screening rather than diagnostic confirmation.
