# Supplementary material for: EEG spectral biomarkers of postoperative delirium in spinal surgery: A high-resolution analysis
Source: PLoS One. 2026 Jul 30;21(7):e0352607. doi: 10.1371/journal.pone.0352607 (PMC13423033; doi:10.1371/journal.pone.0352607)
Supplement: Attachment — Submitted filename: PONE-D-25-68816 reviewer with revision notes.docx [file pone.0352607.s008.docx]

**PLOS One**

**EEG spectral biomarkers of postoperative delirium in spinal surgery: a high-resolution analysis**

--Manuscript Draft--

| **Manuscript Number:** | PONE-D-25-68816 |
| --- | --- |
| **Article Type:** | Research Article |
| **Full Title:** | EEG spectral biomarkers of postoperative delirium in spinal surgery: a high-resolution analysis |
| **Short Title:** | EEG spectral biomarkers of postoperative delirium |
| **Corresponding Author:** | Yasue Mitsukura  Keio University: Keio Gijuku Daigaku Yokohama, Kanagawa JAPAN |
| **Keywords:** | deliriium; Postoperative delirium; EEG; Biomarker |
| **Abstract:** | Background  Postoperative delirium is common in older adults following spinal surgery, yet current EEG-based detection methods rely on broad conventional frequency bands that may obscure clinically meaningful oscillatory changes. More granular spectral approaches may reveal frequency-specific biomarkers that improve diagnostic performance.  Methods  We recorded single-electrode frontal EEG (Fp1) from 47 patients at four perioperative timepoints and compared traditional band-level power with high-resolution 1-Hz spectral analysis (1–45 Hz). Group differences were evaluated using non-parametric statistics with FDR correction, and diagnostic accuracy was assessed using AUROC and threshold-optimized metrics.  Results  Seven patients (14.9%) developed delirium. Conventional band analysis detected only reduced theta power during active delirium (\|r\| = 0.31; AUROC = 0.53). In contrast, 1- Hz analysis revealed a richer pattern: elevated 1-Hz power (\|r\| = 0.27), reduced 3–7 Hz power (\|r\| = 0.25–0.27), and increased 14–15 Hz power (\|r\| = 0.22–0.26). The 23-Hz narrow-band feature achieved the best discrimination (AUROC = 0.74), despite showing no significant effect within the traditional beta band (12–30 Hz). Several frequencies remained altered even after clinical resolution of delirium.  Conclusions  High-resolution spectral analysis identifies discrete EEG biomarkers that outperform conventional frequency bands for postoperative delirium detection. Broad-band averaging masks physiologically meaningful oscillatory signatures, thus adopting finer spectral resolution may enhance the utility of single-channel EEG for routine clinical monitoring. |
| **Order of Authors:** | Gengtao Lin |
|  | Takahito Uchida |
|  | Kota Watanabe |
|  | Satoshi Suzuki |
|  | Masaya Nakamura |
|  | Yasue Mitsukura |
|  | Hiroyoshi Takeuchi |
| **Additional Information:** |  |

| **Question** | **Response** |
| --- | --- |
| **Financial Disclosure**  Enter a financial disclosure statement that describes the sources of funding for the work included in this submission. Review the [submission guidelines](http://journals.plos.org/plosone/s/submission-guidelines#loc-financial-disclosure-statement) for detailed requirements. View published research articles from [*PLOS ONE*](https://journals.plos.org/plosone/) for specific examples.  This statement is required for submission and **will appear in the published article** if the submission is accepted. Please make sure it is accurate. | The author(s) received no specific funding for this work. |
| **Funded studies**  Enter a statement with the following details:   - Initials of the authors who received each award - Grant numbers awarded to each author - The full name of each funder - URL of each funder website - Did the sponsors or funders play any role in the study design, data collection and analysis, decision to publish, or preparation of the manuscript? |  |
| Did you receive funding for this work? |  |
| **Competing Interests**  Use the instructions below to enter a competing interest statement for this submission. On behalf of all authors, disclose any [competing interests](http://journals.plos.org/plosone/s/competing-interests) that could be perceived to bias this work—acknowledging all financial support and any other relevant financial or non- financial competing interests.  This statement is required for submission and **will appear in the published article** if the submission is accepted. Please make sure it is accurate and that any funding sources listed in your Funding Information later in the submission form are also | The authors have declared that no competing interests exist. |

| declared in your Financial Disclosure statement.  View published research articles from  [*PLOS ONE*](http://journals.plos.org/plosone/) for specific examples. |  |
| --- | --- |
| **NO authors have competing interests**  Enter: *The authors have declared that no competing interests exist.*  **Authors with competing interests**  Enter competing interest details beginning with this statement:  *I have read the journal's policy and the authors of this manuscript have the following competing interests: [insert competing interests here]* |  |
| * typeset |  |
| **Ethics Statement**  Enter an ethics statement for this submission. This statement is required if the study involved:   - Human participants - Human specimens or tissue - Vertebrate animals or cephalopods - Vertebrate embryos or tissues - Field research   Write "N/A" if the submission does not require an ethics statement.  General guidance is provided below. Consult the [submission guidelines](http://journals.plos.org/plosone/s/submission-guidelines#loc-guidelines-for-specific-study-types) for detailed instructions. **Make sure that all information entered here is included in the Methods section of the manuscript.** | This proposed observational study was conducted by the collaboration between the Department of Neuropsychiatry and the Department of Orthopedic Surgery at Keio University Hospital (Tokyo, Japan). The Ethics Committee of the Medical School of Keio University reviewed and approved the study protocol (IRB approval number: 20180257). All procedures were aligned with the Declaration of Helsinki guidelines for research involving human subjects. Enrolled participants received both written and verbal explanations about the study, and written informed consent were collected. The data were analyzed anonymously |

| **Format for specific study types**  **Human Subject Research (involving human participants and/or tissue)**   - Give the name of the institutional review board or ethics committee that approved the study - Include the approval number and/or a statement indicating approval of this research - Indicate the form of consent obtained (written/oral) or the reason that consent was not obtained (e.g. the data were analyzed anonymously)   **Animal Research (involving vertebrate animals, embryos or tissues)**   - Provide the name of the Institutional Animal Care and Use Committee (IACUC) or other relevant ethics board that reviewed the study protocol, and indicate whether they approved this research or granted a formal waiver of ethical approval - Include an approval number if one was obtained - If the study involved *non-human primates*, add *additional details* about animal welfare and steps taken to ameliorate suffering - If anesthesia, euthanasia, or any kind of animal sacrifice is part of the study, include briefly which substances and/or methods were applied   **Field Research**  Include the following details if this study involves the collection of plant, animal, or other materials from a natural setting:   - Field permit number - Name of the institution or relevant body that granted permission |  |
| --- | --- |
|  |  |
| **Data Availability**  Authors are required to make all data underlying the findings described fully available, without restriction, and from the time of publication. PLOS allows rare exceptions to address legal and ethical concerns. See the [PLOS Data Policy](http://journals.plos.org/plosone/s/data-availability) and [FAQ](http://journals.plos.org/plosone/s/data-availability#loc-faqs-for-data-policy) for detailed information. | No - some restrictions will apply |

| A Data Availability Statement describing where the data can be found is required at submission. Your answers to this question constitute the Data Availability Statement and **will be published in the article**, if accepted. |  |
| --- | --- |
| **Important:** Stating ‘data available on request from the author’ is not sufficient. If your data are only available upon request, select ‘No’ for the first question and explain your exceptional situation in the text box. |  |
| Do the authors confirm that all data underlying the findings described in their manuscript are fully available without restriction? |  |
| **Describe where the data may be found in full sentences. If you are copying our sample text, replace any instances of XXX with the appropriate details.**   - If the data are **held or will be held in a public repository**, include URLs, accession numbers or DOIs. If this information will only be available after acceptance, indicate this by ticking the box below. For example: *All XXX files are available from the XXX database (accession number(s) XXX, XXX.*). - If the data are all contained **within the manuscript and/or Supporting Information files**, enter the following: *All relevant data are within the manuscript and its Supporting Information files.* - If neither of these applies but you are able to provide **details of access elsewhere**, with or without limitations, please do so. For example:   *Data cannot be shared publicly because of [XXX]. Data are available from the XXX Institutional Data Access / Ethics Committee (contact via XXX) for researchers who meet the criteria for access to confidential data.*  *The data underlying the results presented in the study are available from (include the name of the third party* | Data cannot be shared publicly due to the restriction from the Ethics Committee of the Medical School of Keio University. Researchers may contact the corresponding author via [mitsukura@keio.jp](mailto:mitsukura@keio.jp) for approval. Data will be made available on reasonable request for those who meet the criteria. |

| *and contact information or URL).*   - This text is appropriate if the data are owned by a third party and authors do not have permission to share the data.   * typeset |  |
| --- | --- |
| Additional data availability information: |  |

<https://www2.cloud.editorialmanager.com/pone/download.aspx?id=43359950&guid=6c479b8d-e31d-4e3c-9b4e-4e8d26c40ac1&scheme=1>Manuscript	[Click here to](https://www2.cloud.editorialmanager.com/pone/download.aspx?id=43359950&guid=6c479b8d-e31d-4e3c-9b4e-4e8d26c40ac1&scheme=1)

[access/download;Manuscript;Delirium_manuscript_ver5_mitsu_0](https://www2.cloud.editorialmanager.com/pone/download.aspx?id=43359950&guid=6c479b8d-e31d-4e3c-9b4e-4e8d26c40ac1&scheme=1)

1	EEG spectral biomarkers of postoperative delirium in

2	spinal surgery: a high-resolution analysis

3 **Gengtao Lin ^1^, Takahito Uchida ^2,3^, Kota Watanabe ^4^, Satoshi Suzuki ^4^, Masaya Nakamura^4^, Yasue Mitsukura ^5*^,**
4 **Hiroyoshi Takeuchi ^2^**
5 ^1^ Graduate School of Science and Technology, Keio University, Yokohama, Kanagawa, Japan.
6 ^2^ Department of Neuropsychiatry, School of Medicine, Keio University, Tokyo, Japan.
7 ^3^ Uchida Clinic, Tokyo, Japan
8 ^4^ Department of Orthopedic Surgery, School of Medicine, Keio University, Tokyo, Japan.
9 ^5^ Department of System Design Engineering, Faculty of Science and Science, Keio University, Yokohama, Kanagawa, Japan.

#### 10

11 ^*^ Corresponding author
12 Yasue Mitsukura, Email: [mitsukura@keio.jp](mailto:mitsukura@keio.jp)

# 13 Abstract

*Revision note: Please frame this study explicitly as an exploratory pilot study. Only seven delirium cases were included. Diagnostic thresholds were derived and tested in the same dataset without cross validation. Conclusions should be moderated to state that candidate frequency specific features were identified in this cohort and require validation.*

*Revision note: Moderate the conclusions. Replace statements suggesting identification of biomarkers with wording indicating candidate frequency specific features observed in this dataset. State clearly that larger prospective validation studies are required before clinical implementation.*

14 **Background:**
15 Postoperative delirium is common in older adults following spinal surgery, yet current EEG-based detection methods rely on
16 broad conventional frequency bands that may obscure clinically meaningful oscillatory changes. More granular spectral
17 approaches may reveal frequency-specific biomarkers that improve diagnostic performance.

*Revision note: Remove statements implying absence of overfitting. Performance metrics were derived and tested in the same dataset and may be optimistic. The 23 Hz feature should be described as a candidate marker requiring validation.*

18 **Methods:**
19 We recorded single-electrode frontal EEG (Fp1) from 47 patients at four perioperative timepoints and compared traditional
20 band-level power with high-resolution 1-Hz spectral analysis (1–45 Hz). Group differences were evaluated using non-
21 parametric statistics with FDR correction, and diagnostic accuracy was assessed using AUROC and threshold-optimized
22 metrics.
23 **Results:**
24 Seven patients (14.9%) developed delirium. Conventional band analysis detected only reduced theta power during active
25 delirium (|r| = 0.31; AUROC = 0.53). In contrast, 1-Hz analysis revealed a richer pattern: elevated 1-Hz power (|r| = 0.27),
26 reduced 3–7 Hz power (|r| = 0.25–0.27), and increased 14–15 Hz power (|r| = 0.22–0.26). The 23-Hz narrow-band feature
27 achieved the best discrimination (AUROC = 0.74), despite showing no significant effect within the traditional beta band (12–
28 30 Hz). Several frequencies remained altered even after clinical resolution of delirium.
29 **Conclusions:**
30 High-resolution spectral analysis identifies discrete EEG biomarkers that outperform conventional frequency bands for
31 postoperative delirium detection. Broad-band averaging masks physiologically meaningful oscillatory signatures, thus adopting
32 finer spectral resolution may enhance the utility of single-channel EEG for routine clinical monitoring.

33

# 34 Introduction

35 Postoperative delirium is a frequent and serious complication following spinal surgery, with reported incidence ranging from
36 8.4% to 40.5% in older adults. [1,2]. Yet more than half of cases remain undetected in routine practice. This neuropsychiatric
37 syndrome is characterized by its unpredictable change. Patients may alternate between agitation and withdrawal, making
38 bedside diagnosis difficult [3]. Undetected delirium contributes to worse outcomes: increased mortality, prolonged
39 hospitalization, long-term cognitive impairment, and a four-fold increase in institutionalization after discharge [4,5]. These
40 consequences also impose substantial economic burdens, with an estimated additional annual cost of $44,291 per case [6].
41 This problem is poised to intensify. As global populations age, the number of elderly patients undergoing surgery will rise,
42 and with it, the burden of postoperative delirium. Japan offers a clear example into this future. According to the annual report
43 from the Cabinet Office of Japan, it is estimated that people aged 65 and older will make up 34.8% of the population by 2040
44 [7]. Although studies report delirium in 15–53% of elderly orthopedic patients, national registry data show a diagnosis rate of
45 only 0.2% among surgical cases. Most diagnosed patients (88.2%) require antipsychotic medication, indicating that detection
46 tends to occur only after severe clinical deterioration [8,9]. This gap does not reflect poor clinician awareness but rather
47 fundamental limitations in current delirium assessment methods.

*Revision note: Expand this section to explicitly include: small number of delirium cases; high number of frequency comparisons; lack of multivariable adjustment for perioperative confounders; absence of cross validation; reliance on relative power; single electrode limitation; and T3-T4 analyses based on only two patients. State clearly that findings are hypothesis generating.*

## 48 Current detection paradigms fail to capture delirium's complexity

49 The Confusion Assessment Method for Intensive Care Units (CAM-ICU) is a widely used bedside screening tool for delirium
50 diagnosis. Research studies report acceptable performance with pooled sensitivity of 0.78 (95% CI: 0.72-0.83) and specificity
51 of 0.95 (95% CI: 0.92-0.97) [10]; however, its real-world application shows concerning gaps: Bedside sensitivity drops to
52 38% in cardiac surgery ICU patients, while showing limited detection in non-ICU settings [11,12].
53 Several factors might contribute to the failure. The hypoactive subtype of delirium accounts for 47.2% of cases in elderly
54 surgical patients [13]. These withdrawn patients often go unnoticed because they appear calm and cooperative, not obviously
55 delirious. Also, symptom fluctuations often peak during evening hours (17:00-01:00) when nursing ratios are lowest [14],
56 while most hospitals screen during day shifts. Moreover, the CAM-ICU usually requires 15-20 minutes per assessment and
57 specialized training. One prior work [15] reported that bedside nurses using CAM-ICU detected delirium in only 21.3% of
58 patients compared to 36.7% identified through unstructured clinical assessment, suggesting the tool may underdiagnose
59 delirium when implemented in busy surgical wards.

## 60 EEG monitoring and methodological gaps

61 Electroencephalography (EEG) provides an objective measure of cortical function and is well-suited for detecting
62 neurophysiological disturbances underlying delirium. Across more than 30 studies, delirium patients showed remarkably
63 consistent EEG spectral patterns: increased relative delta power (1-4 Hz), decreased alpha power (8-13 Hz) with reduction in
64 functional connectivity, and increased theta/alpha ratio in delirious patients [16,17].
65 The diagnostic value of these spectral biomarkers has been validated across different populations and settings. Van der Kooi
66 et al. achieved a perfect sensitivity of 100% with 96% specificity using relative delta power from a single frontal-parietal
67 derivation (F8-Pz) in 456 cardiothoracic surgery patients [18]. Later multicenter validation by Numan et al. confirmed that
68 single-channel relative delta power measurements maintained strong performance (AUROC=0.78, 95% CI: 0.72-0.84) across
69 159 postoperative patients from different surgical specialties [19]. Fleischmann et al. further showed that frequency-specific
70 analysis achieved 100% sensitivity with 95-96% specificity in a large retrospective case-control study [20].
71 Shinozaki et al. developed a bispectral EEG (BSEEG) device using dual frontal electrodes (Fp1-Fp2) that measures delirium
72 severity through slow-to-fast wave power ratios [21]. Initial validation in 45 patients achieved 80% sensitivity and 87.7%
73 specificity. Later studies in 1,077 delirium patients showed significant mortality prediction (HR=1.33, 95% CI: 1.16-1.52)
74 [22,23]. The BSEEG score correlates with delirium presence and clinical outcomes including falls, length of stay, and 90-day
75 mortality [24]. Recent improvements have enhanced the BSEEG approach. Topological data analysis applied to raw EEG
76 signals improved classification accuracy by capturing nonlinear dynamics invisible to traditional spectral analysis [25]. The
77 algorithm distinguishes delirium motor subtypes, hyperactive, hypoactive, and mixed, through distinct spectral signatures,
78 addressing variability that confounds behavioral assessments. These single-channel EEG studies have shown strong
79 diagnostic performance, and simplified bispectral devices have demonstrated prognostic value in large cohorts. These
80 findings highlight EEG as a promising candidate for delirium monitoring.
81 However, nearly all delirium EEG studies rely on conventional frequency bands (delta, theta, alpha, beta, gamma) that
82 average spectral power across broad ranges [26]. This approach assumes pathological activity distributes uniformly within
83 these arbitrary boundaries. Study in other domains shows that high-resolution spectral analysis can reveal clinically
84 significant oscillatory patterns invisible to band-averaged approaches [27].
85 The spinal surgery population presents distinct neurophysiological responses. One pilot investigation (N=37) specifically
86 examined patients with spinal surgery, finding paradoxical increases in high-frequency activity rather than the typical
87 slowing pattern associated with delirium [28]. Also, spinal procedures involve unique physiological stressors: prone
88 positioning affecting cerebral perfusion, averaged 800-1200 mL blood loss, prolonged operative duration, and high-dose
89 corticosteroid administration [29,30]. These factors may shape delirium-related brain activity in population-specific ways.
90 Yet no prior study has systematically evaluated high-resolution spectral changes in spinal surgery.

## 91 Study objectives and contributions

*Revision note: The objectives should emphasize exploratory signal identification rather than enhanced detection performance. Please soften language suggesting superiority over conventional bands.*

92 The present study addresses these gaps by applying 1-Hz resolution spectral analysis to single-electrode EEG recordings
93 collected across perioperative timepoints. Our objectives were to (1) identify frequency-specific spectral differences between
94 delirium and non-delirium patients, (2) characterize the perioperative evolution of these patterns, and (3) evaluate the
95 diagnostic performance of narrow-band EEG features compared with traditional frequency bands. This work seeks to
96 determine whether finer spectral resolution can enhance delirium detection while preserving the practicality of single-
97 electrode EEG for routine clinical monitoring.
98 The rest of this paper proceeds as follows: Section 2 details methodology including patient recruitment, EEG acquisition
99 protocols, and spectral analysis techniques. Section 3 presents results examining frequency-specific features and diagnostic
100 performance metrics and discusses findings in context of existing literatures and explores implications for clinical
101 implementation. Finally, the paper is concluded in Section 4 by summarizing the proposed study.

# 102 Methods

## 103 Study design and participant recruitment

104 This proposed observational study was conducted by the collaboration between the Department of Neuropsychiatry and the
105 Department of Orthopedic Surgery at Keio University Hospital (Tokyo, Japan). The Ethics Committee of the Medical School
106 of Keio University reviewed and approved the study protocol (IRB approval number: 20180257). All procedures were
107 aligned with the Declaration of Helsinki guidelines for research involving human subjects. Enrolled participants received
108 both written and verbal explanations about the study, and written informed consent were collected. As compensation for the
109 participation, each person received 3,000 yen upon completing the study.
110 We recruited patients scheduled for spinal surgery between March 12^th^, 2018 and March 17^th^, 2023. The inclusion criteria are
111 as follows: 1) age at least 60 years old, 2) Mini-Mental State Examination (MMSE) score > 23, and 3) Glasgow Coma Scale
112 (GCS) score > 12. We excluded patients with 1) a history of epilepsy or currently under epilepsy treatment, 2) impaired
113 cognitive capacity (MMSE ≤ 23), and 3) significantly impaired consciousness at baseline (GCS ≤ 12). Additionally, we also
114 excluded subjects with incomplete CAM assessment or EEG recording for the subsequent analysis.
115 Furthermore, surgical procedures for all participants were performed under general anesthesia using isoflurane (1.5% for
116 induction, 0.5% for maintenance) with lidocaine for local anesthesia at the surgical site. Postoperative analgesia, including
117 acetaminophen and non-steroidal anti-inflammatory drugs, were administered based on clinical need. All postoperative EEG
118 recordings were obtained after complete recovery from anesthesia.

## 119 Clinical assessment and delirium diagnosis

120 We conducted clinical assessments at four timepoints. T1 occurred preoperatively on the day of hospital admission. T2 took
121 place on postoperative day 1. T3 spanned postoperative days 2 through 4. T4 covered postoperative days 5 through 7 or
122 occurred at discharge if patients left earlier. Each timepoint allowed up to 24 hours for completion to accommodate changes
123 in patient availability and clinical status.
124 Delirium assessment used the confusion assessment method (CAM), an instrument with established validity in surgical
125 populations. The CAM evaluates four cardinal features of delirium: (1) acute onset and fluctuating course, (2) inattention, (3)
126 disorganized thinking, and (4) altered level of consciousness. A positive diagnosis requires features 1 and 2, plus either
127 feature 3 or 4. Trained medical school students conducted all CAM assessments under the supervision of psychiatrists to
128 ensure consistency and minimize variation between raters. We also administered the Glasgow coma scale (GCS), providing
129 an objective measure of consciousness level at each timepoint. Additionally, we asked participants to complete a self-report
130 questionnaire about sleep quality. The assessments taken are summarized in Table S1.
131 Medical records provided information on routine clinical tests performed as standard care, including vital signs, blood tests,
132 and brain imaging. We extracted relevant background factors from these records. All data underwent anonymization and
133 secure storage with access restricted to authorized personnel only.

## 134 EEG Acquisition

135 We acquired EEG data using the portable recording system neuroNicle (LAXTHA, Inc., Korea), a single-channel headband
136 device that operates at a sampling frequency of 512 Hz. The device connects via Bluetooth and was monitored using a tablet
137 during recording sessions. The recording electrode was placed at Fp1 (left prefrontal cortex) according to the international
138 10-20 system, with the reference electrode at A1 (left earlobe). Before each recording, we confirmed that participants were
139 fully alert by checking for eye opening and response to simple commands. If the participant appeared drowsy, we postponed
140 the recording and attempted it later during the observation period.
141 Each recording session lasted approximately 6 minutes. Participants were asked to sit comfortably in a chair when possible. If
142 pain or medical restrictions prevented sitting, they remained in a supine position. We instructed participants to close their
143 eyes and minimize head and body movements during recording. To maintain alertness and prevent drowsing, we asked them
144 to count upward mentally from 1 for the first 180 seconds, then count downward from the highest number reached for the
145 next 180 seconds. This mental task maintains arousal without introducing excessive cognitive load or requiring verbal
146 responses that might introduce artifacts. During recording, the specialist monitored participants continuously to ensure eyes
147 remained closed and no sleep occurred. If we observed signs of drowsing, such as head nodding or lack of response to gentle
148 verbal prompts, we terminated that recording immediately and repeated the measurement after the participant was fully alert.
149 The real-time artifact removal system automatically identifies and filters body movement artifacts and electrical noise during
150 recording. The research staff judged that excessive movement or other disturbances compromised recording quality despite
151 the automatic filtering, we repeated the measurements during the observation period to ensure sufficient data quality for
152 subsequent analysis.

## 153 Data preprocessing and spectral analysis

154 All preprocessing and analysis were done with customized codes in MATLAB R2023a (MathWorks, MA, USA). The raw
155 EEG data were downsampled to 256 Hz and band-pass filtered between 0.5 and 45 Hz using a Butterworth filter. The lower
156 cutoff at 0.5 Hz removed slow DC drift and sweat artifacts. The upper cutoff at 45 Hz excluded high-frequency muscle
157 artifacts and electrical noise while retaining the gamma band. The initial 1-minute recording was removed to keep the stable
158 EEG quality.
159 We divided the filtered EEG data into non-overlapping 5-second windows. This window length provides sufficient data for
160 reliable spectral estimation while capturing temporal dynamics at clinically relevant timescales. Windows containing
161 excessive artifacts were identified through visual inspection and excluded from further analysis. We discarded any windows
162 where signal amplitude exceeded ±200 μV.
163 For each 5-second window, we computed the power spectral density using Welch's method [31]. We applied 1-second Hann
164 window segments with 50% overlap. We then calculated power in five conventional frequency bands: delta (1-4 Hz), theta
165 (4-8 Hz), alpha (8-12 Hz), beta (12-30 Hz), and gamma (30-45 Hz). For each frequency band, we summed the spectral power
166 across all frequency bins within that band to obtain absolute band power. We then calculated total power as the sum of all
167 five band powers. Relative power for each band was computed as the absolute band power divided by total power, expressed
168 as a percentage.
169 Additionally, we computed power at 1-Hz resolution across the entire spectrum. For each integer frequency 𝑓 from 1 to 45
170 Hz, we calculated power in a 1-Hz bin centered at that frequency (spanning from 𝑓 − 0.5 to 𝑓 + 0.5 Hz). We then expressed
171 this power as a percentage of total power, yielding 45 frequency-specific relative power values for each 5-second window.
172 For each subject and timepoint, we averaged the spectral measures across all valid 5-second windows to obtain a single
173 representative spectrum. This averaging reduces the impact of transient fluctuations and provides a stable estimate of the
174 subject's spectral characteristics at that timepoint. The resulting dataset contained, for each subject at each timepoint, five
175 relative band powers (delta through gamma) and 45 narrow-band relative powers.

## 176 Statistical analysis

*Revision note: Please explicitly state the total number of statistical tests conducted across all timepoints. Clarify that FDR correction was applied separately at each timepoint. Acknowledge that small group size may lead to unstable estimates despite correction. Add a statement that ROC thresholds were optimized and evaluated in the same dataset, and that no external validation was performed. Consider adding a simple logistic regression adjusting for age and sex.*

177 We compared EEG spectral features between participants who developed postoperative delirium (delirium group) and those
178 who did not (non-delirium group). Group assignment was based on CAM assessment, with delirium defined as a positive
179 CAM score at any postoperative timepoint (T2, T3, or T4).
180 For between-group comparisons at each timepoint, statistical inference was performed using Wilcoxon rank-sum test. This
181 non-parametric test does not assume normal distribution and is robust to outliers, making it appropriate for clinical data. We
182 applied this test to both conventional frequency bands (5 comparisons) and 1-Hz resolution data (45 comparisons, one per
183 frequency bin). We calculated effect sizes using the correlation coefficient (r), computed as 𝑟 = 𝑍 / √𝑁, where z is the
184 standardized statistics from the Wilcoxon rank-sum test and N is the total sample size [32,33]. Effect sizes were interpreted
185 according to Gignac and Szodorai's benchmarks derived from individual differences research, where r = 0.10 represents a
186 small effect, r = 0.20 a medium effect, and r = 0.30 a large effect [34]. These benchmarks, based on meta-analytic
187 distributions of effect sizes, provide more empirically grounded thresholds than Cohen's traditional conventions.
188 Additionally, to account for multiple comparisons, we applied the Benjamini-Hochberg False Discovery Rate (FDR)
189 correction at q < 0.05 to all spectral analyses [35]. For traditional frequency band analysis (five bands: delta, theta, alpha,
190 beta, gamma), we corrected across the 5 band-wise comparisons at each timepoint. For high-resolution spectral analysis, we
191 corrected across all 45 one-Hz frequency bins at each timepoint.
192 Also, we computed bootstrap confidence intervals for group means using 2,000 bootstrap resamples [36]. Bootstrap
193 resampling is a non-parametric approach that makes no assumptions about the underlying distribution of the data, making it
194 particularly suitable for small sample sizes where normality cannot be reliably assessed. For each bootstrap iteration, we
195 randomly sampled with replacement from the original data, calculated the mean, and stored the result. The 95% confidence
196 interval was defined as the 2.5th and 97.5th percentiles of the bootstrap distribution. Importantly, bootstrap resampling was
197 used solely for confidence interval estimation.
198 To examine temporal change of spectral features, we performed paired comparisons between timepoints within the delirium
199 group. We compared T1 (preoperative baseline) against each postoperative timepoint (T2, T3, T4) using the Wilcoxon
200 signed-rank test for each delirium subject.
201 For diagnostic performance evaluation, we calculated sensitivity, specificity, positive predictive value (PPV), negative
202 predictive value (NPV), and likelihood ratios for selected EEG features [37]. We classified each observation as delirium or
203 non-delirium based on whether its EEG feature value exceeded the optimal threshold. This classification produced four
204 outcomes: true positives (TP, delirium cases correctly identified as delirium), true negatives (TN, non-delirium cases
205 correctly identified as non-delirium), false positives (FP, non-delirium cases incorrectly classified as delirium), and false
206 negatives (FN, delirium cases incorrectly classified as non-delirium). Thus, we computed aforementioned diagnostic metrics
207 by:

#### 208

209

𝑆𝑒𝑛𝑠𝑖𝑡𝑖𝑣𝑖𝑡𝑦 =

𝑆𝑝𝑒𝑐𝑖𝑓𝑖𝑐𝑖𝑡𝑦 =

𝑇𝑃

𝑇𝑃 + 𝐹𝑁

𝑇𝑁

𝑇𝑁 + 𝐹𝑃

(1)

(2)

#### 210

211

𝑃𝑃𝑉 =

𝑁𝑃𝑉 =

𝑇𝑃

𝑇𝑃 + 𝐹𝑃

𝑇𝑁

𝑇𝑁 + 𝐹𝑁

𝑆𝑒𝑛𝑠𝑖𝑡𝑖𝑣𝑖𝑡𝑦

(3)

(4)

#### 212

𝑃𝑜𝑠𝑖𝑡𝑖𝑣𝑒 𝐿𝑖𝑘𝑒𝑙𝑖ℎ𝑜𝑜𝑑 𝑟𝑎𝑡𝑖𝑜 (𝐿𝑅 +) =	(5)

1 − 𝑆𝑝𝑒𝑐𝑖𝑓𝑖𝑐𝑖𝑡𝑦

1 − 𝑆𝑒𝑛𝑠𝑖𝑡𝑖𝑣𝑖𝑡𝑦

#### 213

214

𝑁𝑒𝑔𝑎𝑡𝑖𝑣𝑒 𝐿𝑖𝑘𝑒𝑙𝑖ℎ𝑜𝑜𝑑 𝑟𝑎𝑡𝑖𝑜 (𝐿𝑅 −) =

(6)

𝑆𝑝𝑒𝑐𝑖𝑓𝑖𝑐𝑖𝑡𝑦

215 We determined optimal classification thresholds using Youden's J statistic, 𝐽 = 𝑆𝑒𝑛𝑠𝑖𝑡𝑖𝑣𝑖𝑡𝑦 + 𝑆𝑝𝑒𝑐𝑖𝑓𝑖𝑐𝑖𝑡𝑦 − 1, and
216 constructed receiver operating characteristic (ROC) curves to compute the area under the curve (AUROC) [38,39].
217 Bootstrapped confidence intervals also provided uncertainty estimates for these metrics.

218

# 219 Results and Discussion

220 The following section aims to present findings and our interpretation for the proposed study. This section presents our
221 findings and interpretation. We begin with cohort characteristics and delirium incidence. We then examine spectral
222 differences using conventional frequency bands. Building on these findings, we apply high-resolution 1-Hz analysis to
223 identify specific frequencies. We also examine within-subject temporal changes in delirium patients. Finally, we evaluate

*Revision note: Interpret within-subject findings cautiously given only seven delirium cases. Avoid mechanistic claims regarding hippocampal circuits unless directly tested.*

224 diagnostic performance through ROC analysis.

## 225 Study cohort and demographic characteristics

226 Initial screening identified 127 potential participants and only selected 76 eligible participants upon further inspection. Prior
227 to the clinical recording, 18 participants were excluded due to logistical constraints, and two participants became physically
228 unable to provide testing due to unexpected medical complications. After recording, 9 participants either withdrew from
229 postoperative measurements or produced EEG signals too unclear for reliable analysis. Our final analysis included 47
230 patients. The summary of the screening process is summarized in Fig 1 below.

#### 231

232	**Fig 1.** Cohort screening flow.

#### 233

234 Forty-seven patients completed the study with analyzable EEG recordings. Seven patients (14.9%) developed postoperative
235 delirium based on CAM assessment. The remaining 40 patients showed no delirium throughout the observation period. Table
236 1 presents demographic and clinical characteristics of both groups. Mean age was comparable between control (72.9 ± 7.2
237 years) and delirium (73.4 ± 7.8 years) groups. The delirium group had a higher proportion of male participants (71.4%)
238 compared to the control group (52.5%), though the small sample size of the delirium group limits interpretation of this
239 difference. Relevant medical history includes hypertension, heart disease, diabetes, and prior delirium episodes.

#### 240

241

**Table 1.** Demographic details of participants

242 EEG data completeness varied across the four assessment timepoints. Data availability decreased at subsequent time points
243 due to patient fatigue, medical procedures, early discharge, or signal quality issues. The details of participants with valid EEG
244 data at each timepoint are presented in Table S2. Spectral analysis was performed on 5-second non-overlapping EEG
245 windows, with distribution across timepoints summarized in Table 2 below.

#### 246

247

**Table 2.** Data distribution of 5-second EEG windowed data

248 The observed delirium incidence of 14.9% falls within the range of published estimates for spinal surgery populations (8.4%
249 - 40.5%). All seven delirium cases showed positive CAM scores only at T2 (postoperative day 1). By T3 and T4, these
250 patients had returned to CAM-negative status. This pattern suggests that delirium in our cohort was acute and resolved within
251 24-48 hours. At T1 and T2, all 7 delirium patients contributed EEG data; however, by T3 and T4, only 2 delirium patients
252 remained hospitalized with analyzable recordings. This drop limits the reliability of later timepoint findings.

253

## 254 Between-group EEG spectral comparisons

*Revision note: Avoid attributing physiological meaning to each significant frequency. Focus interpretation on broader patterns. Clarify whether absolute power was analyzed, since relative power may reflect redistribution effects.*

255 We compared relative power between groups using standard frequency bands: delta (1-4 Hz), theta (4-8 Hz), alpha (8-12 Hz),
256 beta (12-30 Hz), and gamma (30-45 Hz). Statistical tests used Wilcoxon rank-sum test with FDR correction. Effect sizes are
257 reported as correlation (r), where |𝑟| < 0.1 is small, 0.1 ≤ |𝑟| ≤ 0.3 is typical, and |𝑟| ≥ 0.3 is relatively large.
258 Table 3 and Fig 2 below summarize conventional frequency band comparisons across all time points. Detailed boxplots for
259 EEG band are shown in Fig S1-S5. At preoperative baseline (T1), only theta band power differed significantly between
260 groups. Non-delirium patients demonstrated higher theta power than those who later developed delirium (18.93% [95% CI:
261 18.52-19.37] compared with 17.19% [16.09-18.35], r = 0.10, p = 0.003). Delta, alpha, and beta bands showed significant
262 differences.
263 During clinical delirium on postoperative day 1 (T2), two frequency bands showed significant differences between-groups.
264 Theta power was markedly lower in delirium patients compared to controls (12.51% compared with 17.88%, r = 0.31, p <
265 0.001), representing a medium effect size. Gamma power was modestly elevated in delirium patients (18.57% compared with
266 15.36%, r = 0.09, p = 0.009). Meanwhile, delta, alpha, and beta bands showed no significant differences between-groups.
267 At later timepoints, spectral differences persisted. At T3, theta band power remained significantly lower in the delirium group
268 (14.73% compared with 20.44%, r = 0.23, p < 0.001), alpha power was reduced (7.33% compared with 8.86%, r = 0.14, p <
269 0.001). In addition, both beta (36.92% compared with 31.85%, r = -0.14, p < 0.001) and gamma (19.26% compared with
270 15.12%, r = -0.24, p < 0.001) were elevated. By timepoint T4, all five frequency bands showed significant differences, with
271 the delirium group demonstrating lower delta, theta, and alpha alongside higher beta and gamma (summarized in Table 3).
272 These T3-T4 findings, however, derive from only 2 delirium patients and require cautious interpretation.
273 The reduction in theta power during active delirium differs from commonly reported findings. Most prior studies in cardiac
274 surgery and ICU population report elevated theta. Our findings are consistent with Lee et al. (2022), who also reported theta
275 suppression accompanied by beta and gamma increases in spinal surgery patients [28]. Together, these results suggest that
276 delirium in spinal surgery represents a distinct physiological context. Factors such as prone positioning, fluctuating cerebral
277 perfusion, substantial blood loss, and perioperative steroid administration may contribute to this different spectral signature.
278 Delta power did not provide meaningful discriminative value in our study. This observation is notable because most previous
279 EEG-based delirium research reports delta-dominant patterns. The lack of delta discrimination challenges the assumption that
280 broad frequency bands function as coherent physiological units. Clinically meaningful information can be obscured when
281 heterogeneous sub-frequencies are averaged.

#### 282

283	**Table 3.** EEG band power comparison between groups

#### 284

285	* Effect size |𝑟| – correlation; significance markers: *n.s.* – not significant; *: 𝑝 < 0.05, **: 𝑝 < 0.01, ***: 𝑝 < 0.001

#### 286

287 **Fig 2**. Heatmap of effect sizes for EEG frequency band comparisons across timepoints. Effect sizes are expressed as
288 standardized effect size (r), with positive values (red) indicating higher power in the non-delirium group and negative values
289 (blue) indicating higher power in the delirium group. Rows represent frequency bands (delta, theta, alpha, beta, gamma) and
290 columns represent timepoints (T1-T4). Significant differences after FDR correction are marked with: *p < 0.05, **p < 0.01,
291 ***p < 0.001.

#### 292

293 To further explore within-band difference, we applied 1-Hz resolution analysis across the 1-45 Hz spectrum (as shown in Fig
294 3). All reported findings survived FDR correction at q < 0.05. Effect size patterns across all frequencies and time points are
295 visualized in Fig 4, with detailed statistics provided in Table S3.

#### 296

297 **Fig 3**. High-resolution (1-Hz) spectral power comparisons between delirium and non-delirium groups at T1 (top-left panel),
298 T2 (top-right panel), T3 (bottom-left panel), and bottom-right (bottom-right panel). Relative power is plotted for each 1-Hz
299 frequency bin from 1 to 45 Hz. Blue and red lines represent mean values for control and delirium groups, respectively, with
300 shaded areas indicating 95% confidence intervals. Background shading denotes traditional frequency bands: delta (1-4 Hz),
301 theta (4-8 Hz), alpha (8-12 Hz), beta (12-30 Hz), and gamma (30-45 Hz).
302

303 **Fig 4**. Heatmap of effect sizes for 1-Hz resolution spectral analysis across timepoints. Effect sizes are expressed as
304 Rosenthal's r, with positive values (red) indicating higher power in the control group and negative values (blue) indicating
305 higher power in the delirium group. Rows represent individual frequencies (1-45 Hz) and columns represent timepoints (T1-
306 T4). Significant differences after FDR correction are marked with: *p < 0.05, **p < 0.01, ***p < 0.001.

#### 307

308 At preoperative baseline, we found 26 discrete frequencies were significantly different between non-delirium and delirium
309 group. The most prominent finding was elevated 1 Hz power in patients who later developed delirium (7.12% [6.18-8.22]
310 compared with 3.81% [3.31-4.34], |𝑟| = 0.26, p < 0.001), while the 3 Hz frequency was lower in the delirium-prone group
311 (8.05% compared with 9.75%, |𝑟| = 0.14, p < 0.001). Within the beta band range, frequencies from 21-30 Hz showed
312 consistently lower power in patients who later developed delirium, with the largest effects at 27-28 Hz (|𝑟| = 0.20 − 0.21).
313 Interestingly, the 14-16 Hz low-beta range showed the opposite pattern, with elevated power in delirium-prone patients (15
314 Hz: 2.16% compared with 1.51%, |𝑟| = 0.14, p < 0.001). High gamma frequencies were also elevated preoperatively in
315 those who later developed delirium (45 Hz: 1.32% compared with 1.08%, |𝑟| = 0.19, p < 0.001).
316 At T2, the 1-Hz analysis revealed a distinctive spectral reorganization involving 27 discrete frequencies (Table S3 presents
317 all frequencies with |𝑟| ≥ 0.1). The pattern can be characterized across several spectral domains. At the ultra-slow
318 frequency end, the 1 Hz frequency showed elevation in delirium patients (6.06% compared with 2.51%, |𝑟| = 0.27, p <
319 0.001). In the mid-frequency range, 3-7 Hz showed consistent reductions with effect sizes largest at 3, 5, and 7 Hz (all |𝑟| ≈
320 0.27, p < 0.001), while 8 Hz showed a smaller but significant reduction (|𝑟| = 0.18). Low-beta frequencies (13-16 Hz) were
321 elevated in delirium patients (15 Hz: 3.43% compared with 2.19%, |𝑟| = -0.26, p < 0.001). Conversely, high-beta frequencies
322 (26-32 Hz) were consistently lower in delirium patients (28 Hz: 0.78% compared with 0.95%, |𝑟| = 0.17, p < 0.001). At the
323 highest frequencies, gamma activity (41-45 Hz) showed pronounced elevation, with 45 Hz demonstrating significantly higher
324 power in delirium patients (2.29% compared with 0.97%,|𝑟| = 0.18, p < 0.001). The overall pattern shows elevated activity at
325 the spectral extremes combined with reduced mid-frequency power (3-8 Hz). This suggests complex cortical reorganization
326 during delirium rather than simple global slowing.
327 At T3 and T4, spectral differences persisted and intensified despite clinical delirium resolution. The largest effects at T4 were
328 observed at 15 Hz (|𝑟| = 0.39, p < 0.001) and 16 Hz (|𝑟| = 0.42, p < 0.001), with delirium patients showing significant
329 increase in EEG power (15 Hz: 4.94% compared with 1.41%; 16 Hz: 6.57% compared with 1.86%). These findings,
330 however, were derived from only 2 patients and could not be generalized.

331

## 332 Within-subject temporal changes in delirium patients

333 To examine whether the between-group spectral differences reflect consistent individual-level changes, we analyzed within-
334 subject EEG power changes in the delirium group, comparing postoperative timepoints against preoperative baseline (T1).
335 All seven delirium patients had paired T1-T2 data, while only 2 patients (Subject 11 and Subject 23) remained hospitalized
336 with recordings at T3 and T4 (Table S2).
337 In the analysis of traditional EEG band power, from T1 to T2 (during active delirium), spectral changes showed notable
338 consistency across individuals despite inter-patient variability in absolute values. We observed a decrease in theta power in 6
339 out of 7 patients, with reductions ranging from -12.8% to -41.5%. One notable exception is that one patient (Subject 25)
340 showed increased theta (+12.2%) and a highly atypical profile with a 334% gamma increase, suggesting a possible distinct
341 pathophysiology. Additionally, we found beta power increased in 5 out of 7 patients, and gamma increased in 4 of 7 patients.
342 Delta and alpha showed more variable patterns, with approximately half of patients showing increases and half showing
343 decreases. At later timepoints T3 and T4, although we had declined number of recordings, the two patients with available
344 data both showed persistent theta reduction and beta elevation compared to their own preoperative baselines, despite CAM-
345 negative status at these timepoints. Table 4 below shows the ratio of participants show increasing or decreasing EEG power
346 for all bands.

#### 347

348

**Table 4**. Direction of spectral change from baseline (T1) to delirium onset (T2)

349 Given the strong individual-level consistency observed for theta and beta band power, we examined these bands at 1-Hz
350 resolution to identify which specific frequencies drove these changes. Table 5 summarizes the most consistent changes from
351 T1 to T2.

#### 352

353

**Table 5**. Individual-level spectral changes at 1-Hz resolution power (from T1 to T2)

354 Within the theta range, the lower frequencies (4-6 Hz) showed the most consistent reductions (86%), while 7-8 Hz showed
355 somewhat less consistency (71%). This gradient suggests that the between-group theta difference is driven primarily by the 4-
356 6 Hz sub-band. The gradient within the theta band (stronger effects at 4-6 Hz than 7-8 Hz) the pathological process
357 preferentially affects lower theta frequencies. These frequencies are associated with hippocampal-cortical communication and
358 memory encoding. These frequencies are associated with hippocampal-cortical communication and memory encoding [40],
359 functions that are characteristically impaired in delirium. The gradient of theta suppression across 4–8 Hz supports the idea
360 that specific memory-related circuits are disrupted, rather than a generalized slowing of cortical activity. Band-level analysis
361 cannot capture this specificity.
362 Furthermore, within the beta range, the individual-level analysis revealed an intriguing pattern. Low-beta frequencies (13-15
363 Hz) increased consistently (71-86% of patients), as did high-beta frequencies (25-30 Hz, 71-86% of patients); however, we
364 observed mid-beta frequencies (18-21 Hz) showing mixed responses. The discriminative detection only with 1-Hz resolution,
365 was obscured in the conventional 12–30 Hz beta band due to cancellation of opposing subcomponents. These beta subranges
366 are linked to different cognitive and motor processes [41,42] , and their dissociation may be relevant to different delirium
367 phenotypes, such as hypoactive or hyperactive presentations.
368 Although data from T3 and T4 were limited, recordings from the two patients who remained hospitalized showed persistent
369 abnormalities, particularly increases around 15–16 Hz (|𝑟| = 0.39 − 0.42). While interpretation must be cautious, these
370 findings raise the possibility that delirium involves a subclinical recovery period with ongoing neurophysiological disruption.
371 They are also compatible with the known phenomenon that EEG complexity, connectivity, and variability remain impaired
372 even after clinical delirium resolves.

## 373 Diagnostic performance

374 We evaluated discriminative ability using receiver operating characteristic (ROC) analysis with optimal thresholds
375 determined by Youden's J statistics. Each spectral feature was evaluated independently based on its empirical distribution in
376 delirium versus control groups, with thresholds selected to maximize the sum of sensitivity and specificity. This approach
377 provides transparent, reproducible performance estimates without the risk of overfitting that can inflate machine learning-
378 based metrics in small samples. Fig 5 below present ROC curves comparing the best-performing 1-Hz frequency at each
379 timepoint.

#### 380

381 **Fig 5.** The receiver operating characteristic (ROC) curve of 1-Hz resolution power at each timepoint, T2 (blue), T3 (red), and
382 T4 (green). The area under the ROC curve (AUROC) is labelled in the legend for each feature.
383**Table 6**. Diagnostic performance at each timepoint: traditional Bands and 1-Hz resolution power

#### 384

385 * AUC – area under the ROC curve; PPV – positive predictive value; NPV – negative predictive value; LR+ – positive
386 likelihood ratio; LR- – negative likelihood ratio

#### 387

388 Table 6 above compares diagnostic performance between the best traditional frequency band and the best 1-Hz frequency at
389 each timepoint. At T2, the 23 Hz frequency (AUC = 0.74, 95% CI: 0.71-0.77) outperformed the best traditional band (beta,
390 AUC = 0.68, 95% CI: 0.63-0.874). The 1-Hz approach achieved higher sensitivity (58.6% compared to 39.3%) while
391 maintaining comparable specificity (83.5% compared to 88.8%), resulting in a substantially lower negative likelihood ratio
392 (0.50 compared to 0.68).
393 At T3 and T4, where only 2 delirium patients contributed data, both approaches showed improved discrimination. Traditional
394 bands and 1-Hz features performed comparably at T3 (beta AUC = 0.73, 95% CI: 0.70-0.77; 19 Hz AUC = 0.71, 95% CI:
395 0.66-0.76), while at T4 the 8 Hz frequency (AUC = 0.86, 95% CI: 0.93-0.90) outperformed theta band (AUC = 0.78, 95% CI:
396 0.73-0.83). These later timepoint results should be interpreted as preliminary observations requiring validation in larger
397 cohorts
398 The 1-Hz approach yielded superior diagnostic performance compared with band analysis, particularly during active
399 delirium. The improvement from beta band (AUC = 0.68) to 23 Hz (AUC = 0.74) is clinically meaningful. The 1-Hz
400 approach detected nearly 60% of delirium cases while maintaining over 80% specificity. The traditional beta band detected
401 less than 40%.
402 Moreover, the relatively low positive predictive values across all features (13-43%) reflect the 14.9% delirium prevalence in
403 our sample rather than poor classifier performance. This has practical implications for implementation. In high-prevalence
404 settings such as ICU populations (30-50% delirium rates), PPV would improve substantially. More importantly, the
405 consistently high negative predictive values (>90%) suggest a specific clinical role: screening to rule out delirium. A negative
406 result on a 23 Hz-based classifier would provide over 90% of confidence that the patient does not have delirium, potentially
407 reducing the burden of formal cognitive assessments in busy clinical environments. This application, using EEG as a triage
408 tool rather than a definitive diagnostic, may represent the most practical implementation pathway.
409 Furthermore, to better contextualize these findings, we compared our diagnostic performance against EEG studies for
410 postoperative delirium detection (shown in Table 7 below).
411**Table 7**. Performance comparison with prior EEG delirium studies

#### 412

413	* AUROC – area under the ROC curve; NR – not reported

#### 414

415 The comparison with prior studies reveals population-dependent differences in optimal EEG biomarkers for delirium
416 detection. In ICU patients, van der Kooi et al. achieved 100% sensitivity with 96% specificity using relative delta power from
417 a single frontal channel [18]. Similarly, delta-based features performed well in Numan et al., who reported an AUC of 0.78 in
418 general postoperative elderly patients [19], and in Fleischmann et al., who demonstrated AUC values of 0.82-0.86 in a
419 heterogeneous hospital population [20]. These findings established delta power as the predominant EEG biomarker for
420 delirium across diverse clinical settings. In our spinal surgery cohort, however, delta power provided minimal discriminative
421 value, while beta-range frequencies (23 Hz) performed best at T2.
422 This divergence likely reflects differences in underlying pathophysiology. ICU patients and heterogeneous hospital cohorts
423 frequently include individuals with sepsis, metabolic derangements, or multiorgan dysfunction. These conditions produce
424 characteristic EEG slowing with increased delta activity [43,44]. Spinal surgery patients, by contrast, are typically medically
425 stable but face distinct perioperative stressors. The prone position required for posterior spinal approaches elevates
426 intrathoracic and intraabdominal pressure, impedes cerebral venous drainage, and can reduce middle cerebral artery blood
427 velocity by approximately 10% [45,46]. Additionally, perioperative corticosteroids such as dexamethasone, routinely
428 administered for pain management and nerve protection in spinal surgery [47,48], may modulate neuroinflammatory
429 pathways in ways that differ from non-steroid-treated populations.
430 Furthermore, two recent spinal surgery studies support the notion of population-specific EEG signatures. Lee et al. [28]
431 reported a 23.2% decrease in theta waves (p = 0.016) accompanied by elevated high-beta (19.3%, p = 0.003) and gamma
432 activity (18.8%, p = 0.006) in delirium patients, a pattern similar to our findings. Kim et al. [49] demonstrated that lower
433 preoperative median dominant frequency predicted postoperative delirium in 257 elderly spinal surgery patients (OR = 0.48,
434 95% CI: 0.27-0.85), with their prediction model achieving an AUC of 0.75. The convergence of theta reduction and
435 beta/gamma elevation across these independent spinal surgery cohorts suggests a reproducible, population-specific spectral
436 phenotype rather than methodological artifact.
437 Despite using a single-electrode montage, our diagnostic performance (AUC = 0.74) was comparable to multi-channel
438 approaches. Van der Kooi et al. used a single frontal channel, Numan et al. employed limited-channel recordings, and Kim et
439 al. used two prefrontal electrodes [18,19,49]. This convergence suggests that high-resolution spectral analysis may
440 compensate for limited spatial coverage, supporting the feasibility of simplified EEG monitoring in routine clinical practice.

## 441 Limitations and future direction

442 Several limitations should be considered in the proposed study. First, the sample size was small, particularly at later time
443 points, which limits statistical power and generalizability. Larger multicenter cohort will be needed to validate these
444 preliminary findings. Second, the study population was limited to spinal surgery patients and may not generalize to other
445 surgical types or medical delirium. Third, the mental-counting task used during EEG acquisition may influence theta activity;
446 future research should incorporate both resting-state and task-based recordings. Fourth, the single-electrode montage limits
447 spatial characterization of cortical changes. Finally, delirium subtypes were not examined, and spectral patterns may differ
448 across subtypes. Future studies should also validate narrow-band biomarkers in larger and more diverse patient populations,
449 evaluate whether spatial EEG features further enhance performance, investigate delirium subtypes using combined spectral
450 and behavioral clustering, and develop real-time algorithms for bedside monitoring.

# 451 Conclusions

452 The proposed study shows that high-resolution 1-Hz spectral analysis can identify EEG features that outperform traditional
453 frequency bands for detecting postoperative delirium in spinal surgery patients. By analyzing narrow frequency bins, we
454 found specific markers, theta suppression at 3–7 Hz and elevated power at 23 Hz. These specific features were entirely
455 missed by broad-band analysis, and they showed better diagnostic accuracy during delirium onset, with high negative
456 predictive values that make them well-suited for clinical screening.
457 Our findings challenge the common assumption that traditional frequency bands represent uniform physiological processes.
458 When signals from different frequencies are averaged together, important diagnostic information can be lost. This "dilution
459 effect" likely explains why broad-band theta and delta measures performed poorly despite being the standard approach in
460 most EEG delirium studies.
461 The small sample size limits the strength of these conclusions. Nevertheless, our results suggest that finer spectral resolution
462 offers a practical way to improve EEG-based delirium detection. Multicenter studies with larger patient numbers will be
463 needed to confirm whether these findings apply across different surgical populations.

464

465

466

467	**References**

468	1.	Kang T, Park SY, Lee JH, Lee SH, Park JH, Kim SK, et al. Incidence & Risk Factors of Postoperative Delirium After

469	Spinal Surgery in Older Patients. Sci Rep. 2020;10: 9232. doi:10.1038/s41598-020-66276-3

470	2.	Luo M, Wang D, Shi Y, Yi Q, Wang Z, Zhou B, et al. Risk factors of postoperative delirium following spine surgery:

471	A meta-analysis of 50 cohort studies with 1.1 million participants. Heliyon. 2024;10: e24967.

472	doi:10.1016/j.heliyon.2024.e24967

473	3.	Robinson TN, Raeburn CD, Tran Z V., Angles EM, Brenner LA, Moss M. Postoperative Delirium in the Elderly.

474	Ann Surg. 2009;249: 173–178. doi:10.1097/SLA.0b013e31818e4776

475 4.	Susano MJ, Grasfield RH, Friese M, Rosner B, Crosby G, Bader AM, et al. Brief Preoperative Screening for Frailty
476 and Cognitive Impairment Predicts Delirium after Spine Surgery. Anesthesiology. 2020;133: 1184–1191.
477 doi:10.1097/ALN.0000000000003523
478 5.	Brown CH, LaFlam A, Max L, Wyrobek J, Neufeld KJ, Kebaish KM, et al. Delirium After Spine Surgery in Older
479 Adults: Incidence, Risk Factors, and Outcomes. J Am Geriatr Soc. 2016;64: 2101–2108. doi:10.1111/jgs.14434
480 6.	Gleason LJ, Schmitt EM, Kosar CM, Tabloski P, Saczynski JS, Robinson T, et al. Effect of Delirium and Other
481 Major Complications on Outcomes After Elective Surgery in Older Adults. JAMA Surg. 2015;150: 1134.
482 doi:10.1001/jamasurg.2015.2606
483 7.	Cabinet Office Japan. Annual Report on the Ageing Society FY2024. Jun 2024 [cited 4 Oct 2025]. Available:
484 https://www8.cao.go.jp/kourei/english/annualreport/2024/pdf/2024.pdf
485 8.	Fong TG, Tulebaev SR, Inouye SK. Delirium in elderly adults: diagnosis, prevention and treatment. Nat Rev Neurol.

486	2009;5: 210–20. doi:10.1038/nrneurol.2009.24

487 9.	Ueda N, Igarashi M, Okuyama K, Sano H, Takahashi K, P Qureshi Z, et al. Demographic and clinical characteristics
488 of patients with delirium: analysis of a nationwide Japanese medical database. BMJ Open. 2022;12: e060630.
489 doi:10.1136/bmjopen-2021-060630
490 10.	Miranda F, Gonzalez F, Plana MN, Zamora J, Quinn TJ, Seron P. Confusion Assessment Method for the Intensive
491 Care Unit (CAM-ICU) for the diagnosis of delirium in adults in critical care settings. Cochrane Database of
492 Systematic Reviews. 2023;2023. doi:10.1002/14651858.CD013126.pub2
493 11.	Koga Y, Tsuruta R, Murata H, Matsuo K, Ito T, Ely EW, et al. Reliability and validity assessment of the Japanese
494 version of the Confusion Assessment Method for the Intensive Care Unit (CAM-ICU). Intensive Crit Care Nurs.
495 2015;31: 165–170. doi:10.1016/j.iccn.2014.10.002
496 12.	Neufeld KJ, Leoutsakos JS, Sieber FE, Joshi D, Wanamaker BL, Rios-Robles J, et al. Evaluation of two delirium
497 screening tools for detecting post-operative delirium in the elderly. Br J Anaesth. 2013;111: 612–618.
498 doi:10.1093/bja/aet167
499 13.	Marcantonio ER. Delirium in Hospitalized Older Adults. New England Journal of Medicine. 2017;377: 1456–1466.

500	doi:10.1056/NEJMcp1605501

501 14.	Kanno M, Doi M, Kubota K, Kanoya Y. Risk factors for postoperative delirium and subsyndromal delirium in older
502 patients in the surgical ward: A prospective observational study. PLoS One. 2021;16: e0255607.
503 doi:10.1371/journal.pone.0255607
504 15.	van Eijk MM, van den Boogaard M, van Marum RJ, Benner P, Eikelenboom P, Honing ML, et al. Routine Use of the
505 Confusion Assessment Method for the Intensive Care Unit. Am J Respir Crit Care Med. 2011;184: 340–344.
506 doi:10.1164/rccm.201101-0065OC
507 16.	Wiegand TLT, Rémi J, Dimitriadis K. Electroencephalography in delirium assessment: a scoping review. BMC

508	Neurol. 2022;22: 86. doi:10.1186/s12883-022-02557-w

509	17.	Kim SB, Bong SH, Lee JH, Choi TY, Yoon SY, Kim JW. The Usefulness of Quantitative Electroencephalography in

510	Diagnosis and Severity Evaluation of Delirium: A Retrospective Study. Psychiatry Investig. 2023;20: 144–151.

511	doi:10.30773/pi.2022.0294

512	18.	Van Der Kooi AW, Zaal IJ, Klijn FA, Koek HL, Meijer RC, Leijten FS, et al. Delirium detection using EEG: What

513	and how to measure. Chest. 2015;147: 94–101. doi:10.1378/chest.13-3050

514 19.	Numan T, van den Boogaard M, Kamper AM, Rood PJT, Peelen LM, Slooter AJC, et al. Delirium detection using
515 relative delta power based on 1-minute single-channel EEG: a multicentre study. Br J Anaesth. 2019;122: 60–68.
516 doi:10.1016/j.bja.2018.08.021
517 20.	Fleischmann R, Tränkner S, Bathe-Peters R, Rönnefarth M, Schmidt S, Schreiber SJ, et al. Diagnostic Performance
518 and Utility of Quantitative EEG Analyses in Delirium: Confirmatory Results From a Large Retrospective Case-
519 Control Study. Clin EEG Neurosci. 2019;50: 111–120. doi:10.1177/1550059418767584
520 21.	Shinozaki G, Chan AC, Sparr NA, Zarei K, Gaul LN, Heinzman JT, et al. Delirium detection by a novel bispectral
521 electroencephalography device in general hospital. Psychiatry Clin Neurosci. 2018;72: 856–863.
522 doi:10.1111/pcn.12783
523 22.	Nishizawa Y, Yamanashi T, Saito T, Marra P, Crutchley KJ, Wahba NE, et al. Bispectral EEG (BSEEG) Algorithm
524 Captures High Mortality Risk Among 1,077 Patients: Its Relationship to Delirium Motor Subtype. The American
525 Journal of Geriatric Psychiatry. 2023;31: 704–715. doi:10.1016/j.jagp.2023.03.002
526 23.	Shinozaki G, Bormann NL, Chan AC, Zarei K, Sparr NA, Klisares MJ, et al. Identification of Patients With High
527 Mortality Risk and Prediction of Outcomes in Delirium by Bispectral EEG. J Clin Psychiatry. 2019;80.
528 doi:10.4088/JCP.19m12749
529 24.	Yamanashi T, Crutchley KJ, Wahba NE, Sullivan EJ, Comp KR, Kajitani M, et al. Evaluation of point-of-care
530 thumb-size bispectral electroencephalography device to quantify delirium severity and predict mortality. The British
531 Journal of Psychiatry. 2022;220: 322–329. doi:10.1192/bjp.2021.101
532 25.	Yamanashi T, Kajitani M, Iwata M, Crutchley KJ, Marra P, Malicoat JR, et al. Topological data analysis (TDA)
533 enhances bispectral EEG (BSEEG) algorithm for detection of delirium. Sci Rep. 2021;11: 304. doi:10.1038/s41598-
534 020-79391-y
535 26.	van der Kooi AW, Leijten FSS, van der Wekken RJ, Slooter AJC. What Are the Opportunities for EEG-Based
536 Monitoring of Delirium in the ICU? J Neuropsychiatry Clin Neurosci. 2012;24: 472–477.
537 doi:10.1176/appi.neuropsych.11110347
538 27.	Prerau MJ, Brown RE, Bianchi MT, Ellenbogen JM, Purdon PL. Sleep Neurophysiological Dynamics Through the
539 Lens of Multitaper Spectral Analysis. Physiology. 2017;32: 60–92. doi:10.1152/physiol.00062.2015
540 28.	Lee S-B, Kwon J-W, Sung S, Moon S-H, Lee BH. Delirium after Spinal Surgery: A Pilot Study of
541 Electroencephalography Signals from a Wearable Device. Applied Sciences. 2022;12: 9899.
542 doi:10.3390/app12199899
543 29.	Ushida T, Yokoyama T, Kishida Y, Hosokawa M, Taniguchi S, Inoue S, et al. Incidence and Risk Factors of
544 Postoperative Delirium in Cervical Spine Surgery. Spine (Phila Pa 1976). 2009;34: 2500–2504.
545 doi:10.1097/BRS.0b013e3181b321e6
546 30.	Kawaguchi Y, Kanamori M, Ishihara H, Abe Y, Nobukiyo M, Sigeta T, et al. Postoperative delirium in spine surgery.

547	The Spine Journal. 2006;6: 164–169. doi:10.1016/j.spinee.2005.06.010

548	31.	Welch P. The use of fast Fourier transform for the estimation of power spectra: A method based on time averaging

549	over short, modified periodograms. IEEE Transactions on Audio and Electroacoustics. 1967;15: 70–73.

550	doi:10.1109/TAU.1967.1161901

551	32.	Field Andy. Discovering statistics using IBM SPSS statistics. Sage Publications Inc.; 2018.

552	33.	Rosenthal R. Meta-Analytic Procedures for Social Research. 2455 Teller Road, Thousand

553	Oaks California 91320 United States of America : SAGE Publications, Inc.; 1991. doi:10.4135/9781412984997

554	34.	Gignac GE, Szodorai ET. Effect size guidelines for individual differences researchers. Pers Individ Dif. 2016;102:

555	74–78. doi:10.1016/j.paid.2016.06.069

556	35.	Benjamini Y, Hochberg Y. Controlling the False Discovery Rate: A Practical and Powerful Approach to Multiple

557	Testing. J R Stat Soc Series B Stat Methodol. 1995;57: 289–300. doi:10.1111/j.2517-6161.1995.tb02031.x

558	36.	Efron B. Bootstrap Methods: Another Look at the Jackknife. The Annals of Statistics. 1979;7.

559	doi:10.1214/aos/1176344552

560	37.	Šimundić A-M. Measures of Diagnostic Accuracy: Basic Definitions. EJIFCC. 2009;19: 203–11.

561	38.	Hanley JA, McNeil BJ. The meaning and use of the area under a receiver operating characteristic (ROC) curve.

562	Radiology. 1982;143: 29–36. doi:10.1148/radiology.143.1.7063747

563	39.	YOUDEN WJ. Index for rating diagnostic tests. Cancer. 1950;3: 32–5. doi:10.1002/1097-0142(1950)3:1<32::aid-

564	cncr2820030106>3.0.co;2-3

565	40.	Buzsáki G. Theta Oscillations in the Hippocampus. Neuron. 2002;33: 325–340. doi:10.1016/S0896-6273(02)00586-

#### 566 X

567	41.	Pfurtscheller G, Lopes da Silva FH. Event-related EEG/MEG synchronization and desynchronization: basic

568	principles. Clinical Neurophysiology. 1999;110: 1842–1857. doi:10.1016/S1388-2457(99)00141-8

569	42.	Engel AK, Fries P. Beta-band oscillations — signalling the status quo? Curr Opin Neurobiol. 2010;20: 156–165.

570	doi:10.1016/j.conb.2010.02.015

571	43.	Young GB, Bolton CF, Archibald YM, Austin TW, Wells GA. The Electroencephalogram in Sepsis-Associated

572	Encephalopathy. Journal of Clinical Neurophysiology. 1992;9: 145–152. doi:10.1097/00004691-199201000-00016

573	44.	Gofton TE, Young GB. Sepsis-associated encephalopathy. Nat Rev Neurol. 2012;8: 557–566.

574	doi:10.1038/nrneurol.2012.183

575 45.	Højlund J, Sandmand M, Sonne M, Mantoni T, Jørgensen HL, Belhage B, et al. Effect of Head Rotation on Cerebral
576 Blood Velocity in the Prone Position. Anesthesiol Res Pract. 2012;2012: 1–6. doi:10.1155/2012/647258
577 46.	Yeoh TY, Tan A, Manninen P, Chan VWS, Venkatraghavan L. Effect of different surgical positions on the cerebral
578 venous drainage: a pilot study using healthy volunteers. Anaesthesia. 2016;71: 806–813. doi:10.1111/anae.13494
579 47.	Ranguis SC, Li D, Webster AC. Perioperative epidural steroids for lumbar spine surgery in degenerative spinal

580	disease. J Neurosurg Spine. 2010;13: 745–757. doi:10.3171/2010.6.SPINE09796

581 48.	Sharma M, Gupta S, Purohit S, Goyal AK. The Effect of Intravenous Dexamethasone on Intraoperative and Early
582 Postoperative Pain in Lumbar Spine Surgery: A Randomized Double-Blind Placebo-Controlled Study. Anesth Essays
583 Res. 2018;12: 803–808. doi:10.4103/aer.AER_115_18
584 49.	Kim J, Park S, Kim K-N, Ha Y, Shin S-J, Cha W, et al. Resting-state prefrontal EEG biomarker in correlation with
585 postoperative delirium in elderly patients. Front Aging Neurosci. 2023;15. doi:10.3389/fnagi.2023.1224264

586

## 587 Supporting information

588 **Table S1. Summary of assessments at different timepoints.** * “O” – test was conducted; “X” – test was not conducted.
589 **Table S2. Recording Information for Participants**.
590 **Fig S1. Comparisons of relative delta power between groups.** Delta band (1-4 Hz) relative power distribution across
591 timepoints. Boxplots compare non-delirium (blue) and delirium (red) groups. Sample sizes: T1 (non-delirium n = 35,
592 delirium n = 7), T2 (control n = 32, delirium n = 7), T3 (non-delirium n = 26, delirium n = 2), and T4 (non-delirium n = 21,
593 delirium n = 2). Effect sizes are reported as correlation (r). Significance levels after FDR correction: *p < 0.05, **p < 0.01,
594 ***p < 0.001.
595 **Fig S2. Comparisons of relative theta power between groups.**
596 **Fig S3. Comparisons of relative alpha power between groups.**
597 **Fig S4. Comparisons of relative beta power between groups.**
598 **Fig S5. Comparisons of relative gamma power between groups.**
599 **Table S3. Significant 1-Hz resolution EEG power comparisons with medium or above effect size (** |𝒓| > 𝟎. 𝟏**).**

Supporting Information

### [Click here to access/download](https://www2.cloud.editorialmanager.com/pone/download.aspx?id=43153804&guid=295efb48-b6fe-48ed-8e7f-78a44c3819eb&scheme=1)

[**Supporting Information** Plos_One_supportingInfo.docx](https://www2.cloud.editorialmanager.com/pone/download.aspx?id=43153804&guid=295efb48-b6fe-48ed-8e7f-78a44c3819eb&scheme=1)

IRB(approval)

### [Click here to access/download](https://www2.cloud.editorialmanager.com/pone/download.aspx?id=43400685&guid=95e30ae9-edc6-45e7-92db-a1f04816a5d1&scheme=1)

[**Supporting Information** 20180257_approval_of_IRB.pdf](https://www2.cloud.editorialmanager.com/pone/download.aspx?id=43400685&guid=95e30ae9-edc6-45e7-92db-a1f04816a5d1&scheme=1)
