# Supplementary material for: EEG spectral biomarkers of postoperative delirium in spinal surgery: A high-resolution analysis
Source: PLoS One. 2026 Jul 30;21(7):e0352607. doi: 10.1371/journal.pone.0352607 (PMC13423033; doi:10.1371/journal.pone.0352607)
Supplement: Attachment — Submitted filename: Reviewer Comments for PONE.docx [file pone.0352607.s009.docx]

### Reviewer Comments for PONE-D-25-68816

**General Comments** This study investigates the neurophysiological signatures of delirium in patients undergoing spinal surgery using single-channel Fp1 EEG and 1-Hz resolution spectral analysis. The topic is clinically relevant, particularly in the effort to identify low-cost, accessible screening methods and exploring frequency-specific biomarkers (e.g., 23 Hz) beyond traditional band analysis.

However, several significant methodological and statistical limitations substantially weaken the strength of the conclusions. In particular, the very small number of delirium cases raises concerns regarding the robustness, stability, and generalizability of the reported results. Major revisions are required before the manuscript can be considered for publication.

**Major Issues**

**1. Sample Size, Event Rates, and Statistical Stability** The sample size is limited (N = 47), with only 7 cases of delirium (14.9%). More critically, at later time points (T3 and T4), only 2 delirium cases remained. In such instances, any estimates of diagnostic performance are highly unstable and prone to random variation. This limitation is not restricted to the T3/T4 analysis; even the primary findings (e.g., AUROC at T2) may suffer from significant uncertainty due to the low number of events.

**Recommendation:**

(1) The authors must explicitly acknowledge the limited statistical power and instability of effect estimates throughout the manuscript.

(2) Findings at T3 and T4 should be described strictly as exploratory and should not support confirmatory claims.

(3) Primary conclusions should be strictly confined to the most reliable time point (T2) and interpreted with caution.

**2. Confounding Factors and Limitations of Adjustment** There is a notable imbalance in gender distribution (71.4% male in the delirium group vs. 52.5% in the control group), and key variables such as age and baseline cognitive function (MMSE) are established predictors of delirium. These factors may significantly confound the reported association between spectral features and delirium.

**Recommendation:** The authors should consider adjusted analyses (e.g., logistic regression or ANCOVA) to explore whether the observed spectral features retain diagnostic value after accounting for potential confounders. However, given there are only 7 delirium events, any multivariable model would be underpowered and unstable (limited events-per-variable ratio).

**Therefore:**

（1）Any adjusted analysis, if performed, must be treated as purely exploratory.

（2）The lack of robust confounding adjustment must be more clearly acknowledged as a limitation.

**3. Interpretation of Diagnostic Performance** The manuscript highlights an AUROC of 0.74 for the 23 Hz feature. In clinical diagnostics, this level of discrimination is generally considered moderate rather than strong. Furthermore, with such a small number of cases, the estimated AUROC and the optimal cutoff derived from Youden’s J-index are highly susceptible to overfitting.

**Recommendation:**

(1) The authors should temper claims regarding the "superiority" of specific frequencies compared to traditional band-based methods.

(2) Confidence intervals for AUROCs must be explicitly reported and discussed.

(3) If feasible, internal validation (e.g., leave-one-out cross-validation) should be performed to assess the robustness of the findings.

**4. Data Availability** The current statement regarding the inability to share data due to ethical restrictions does not meet PLOS ONE’s Data Availability standards.

**Minor Revisions**

**1.Figure 5:** The ROC curves for T3 and T4 should be removed, as analyses based on N=2 are statistically meaningless.

**2.Terminology:** Replace assertive terms such as "proved" with more appropriate academic hedging (e.g., "suggested" or "associated with").

**3.Limitations:** The manuscript should more prominently and explicitly discuss limitations, specifically small sample size, overfitting, and the risk of false positives.

**Conclusion**

The study addresses an interesting and clinically significant question and introduces a potentially useful analytical approach. However, the very small number of delirium cases severely limits the validity and interpretability of the findings. The manuscript may be considered for publication after major revisions, provided the authors:

1.Appropriately temper their conclusions,

2.Explicitly acknowledge the statistical limitations, and

3.Enhance the methodological rigor and transparency of the analysis.
