# Supplementary material for: EEG spectral biomarkers of postoperative delirium in spinal surgery: A high-resolution analysis
Source: PLoS One. 2026 Jul 30;21(7):e0352607. doi: 10.1371/journal.pone.0352607 (PMC13423033; doi:10.1371/journal.pone.0352607)
Supplement: Attachment — Submitted filename: Response to Reviewers_rev2_0526.docx [file pone.0352607.s012.docx]

**Response to Reviewers**

*Manuscript ID: PONE-D-25-68816*

We sincerely appreciate both the careful and constructive assessment and thank you for recognizing the improvement we have made since last revision. We have addressed each remaining minor point below.

**Reviewer #1**

**1. Comment:**

*Wording in Abstract and Conclusions still slightly stronger than warranted (e.g., "identifies discrete EEG biomarkers," "outperform conventional frequency bands")*

**Response:**

We appreciate this comment. We have further softened the wording in the Abstract and Conclusions as suggested.

**2. Comment:**

*Although the authors appropriately acknowledge the very limited sample at T3 and T4, parts of the interpretation of these later timepoints remain somewhat extensive considering only two delirium patients contributed data. I recommend continuing to emphasize that these observations are descriptive and preliminary.*

**Response:**

We have revised the relevant passages so that the T3 and T4 findings are presented as descriptive and preliminary observations from a very limited number of patients, and we have removed interpretive statements that extend beyond what the data support.

**3. Comment:**

*Minor English editing and copyediting would further improve readability.*

**Response:**

We have carried out a copyediting pass throughout the manuscript and hopefully it will improve the general readability.

**4. Comment:**

*The Data Availability statement has improved substantially; however, the authors should ensure that it fully complies with PLOS ONE policy requirements regarding accessibility of underlying data.*

**Response:**

Thank you for pointing this out. Yes, we have updated our data availability statement and corresponding contact information of the Ethics Committee. The data would be made available on reasonable request, following by screening and approval from the Ethics Committee.
