# Supplementary material for: EEG spectral biomarkers of postoperative delirium in spinal surgery: A high-resolution analysis
Source: PLoS One. 2026 Jul 30;21(7):e0352607. doi: 10.1371/journal.pone.0352607 (PMC13423033; doi:10.1371/journal.pone.0352607)
Supplement: Peer review history

# Peer review: Decision Letter 0

**DOI:** 10.1371/journal.pone.0352607.r001

**Authors:** Hiroki Annaka

27 Mar 2026

PONE-D-25-68816

EEG spectral biomarkers of postoperative delirium in spinal surgery: a high-resolution analysis

PLOS One

Dear Dr. Mitsukura,

-->Thank you for submitting your manuscript to PLOS ONE. After careful consideration, we feel that it has merit but does not fully meet PLOS ONE’s publication criteria as it currently stands. Therefore, we invite you to submit a revised version of the manuscript that addresses the points raised during the review process.

==============================

**Dear author,**

**Please address the comments from the two reviewers.  While the two reviewers have given this study a generally positive evaluation, they have expressed concerns about the accuracy of the results due to the small number of patients who exhibited delirium. While I understand the techniques involved, such as the bootstrap method, I believe this issue requires a detailed explanation.**

**Reviewer 2’s comments in English are attached. Please make revisions based on these comments.**

**I have also attached the revision notes from Reviewer 1.**

==============================

Please submit your revised manuscript by May 10 2026 11:59PM. If you will need more time than this to complete your revisions, please reply to this message or contact the journal office at plosone@plos.org. When you're ready to submit your revision, log on to https://www.editorialmanager.com/pone/ and select the 'Submissions Needing Revision' folder to locate your manuscript file.

Please include the following items when submitting your revised manuscript:

- A letter that responds to each point raised by the academic editor and reviewer(s). You should upload this letter as a separate file labeled 'Response to Reviewers'.
- A marked-up copy of your manuscript that highlights changes made to the original version. You should upload this as a separate file labeled 'Revised Manuscript with Track Changes'.
- An unmarked version of your revised paper without tracked changes. You should upload this as a separate file labeled 'Manuscript'.

If you would like to make changes to your financial disclosure, please include your updated statement in your cover letter. Guidelines for resubmitting your figure files are available below the reviewer comments at the end of this letter.

If applicable, we recommend that you deposit your laboratory protocols in protocols.io to enhance the reproducibility of your results. Protocols.io assigns your protocol its own identifier (DOI) so that it can be cited independently in the future. For instructions see: https://journals.plos.org/plosone/s/submission-guidelines#loc-laboratory-protocols. Additionally, PLOS ONE offers an option for publishing peer-reviewed Lab Protocol articles, which describe protocols hosted on protocols.io. Read more information on sharing protocols at https://plos.org/protocols?utm_medium=editorial-email&utm_source=authorletters&utm_campaign=protocols.

As the corresponding author, your ORCID iD is verified in the submission system and will appear in the published article. PLOS supports the use of ORCID, and we encourage all coauthors to register for an ORCID iD and use it as well. Please encourage your coauthors to verify their ORCID iD within the submission system before final acceptance, as unverified ORCID iDs will not appear in the published article. *Only*  the individual author can complete the verification step; PLOS staff *cannot*  verify ORCID iDs on behalf of authors.

We look forward to receiving your revised manuscript.

Kind regards,

Hiroki Annaka

Academic Editor

PLOS One

Journal Requirements:

When submitting your revision, we need you to address these additional requirements.

1. Please ensure that your manuscript meets PLOS ONE's style requirements, including those for file naming. The PLOS ONE style templates can be found at

https://journals.plos.org/plosone/s/file?id=wjVg/PLOSOne_formatting_sample_main_body.pdf and https://journals.plos.org/plosone/s/file?id=ba62/PLOSOne_formatting_sample_title_authors_affiliations.pdf

2. In the online submission form you indicate that your data is not available for proprietary reasons and have provided a contact point for accessing this data. Please note that your current contact point is a co-author on this manuscript. According to our Data Policy, the contact point must not be an author on the manuscript and must be an institutional contact, ideally not an individual. Please revise your data statement to a non-author institutional point of contact, such as a data access or ethics committee, and send this to us via return email. Please also include contact information for the third-party organization, and please include the full citation of where the data can be found.

3. Thank you for providing your underlying data as Supporting Information.

We note that the data set contains text or data that is not in English. Please note that PLOS is an English-language publisher, so we require data sets to be provided in English as well. Please upload an English-language version of your data set.

This will also allow us to determine if your data follows PLOS standards per our Data Availability policy here: https://journals.plos.org/plosone/s/data-availability

If the reviewer comments include a recommendation to cite specific previously published works, please review and evaluate these publications to determine whether they are relevant and should be cited. There is no requirement to cite these works unless the editor has indicated otherwise.

[Note: HTML markup is below. Please do not edit.]

Reviewers' comments:

Reviewer's Responses to Questions

**Comments to the Author**

1. Is the manuscript technically sound, and do the data support the conclusions?

The manuscript must describe a technically sound piece of scientific research with data that supports the conclusions. Experiments must have been conducted rigorously, with appropriate controls, replication, and sample sizes. The conclusions must be drawn appropriately based on the data presented.

Reviewer #1: Partly

Reviewer #2: Partly

**********

2. Has the statistical analysis been performed appropriately and rigorously?

Reviewer #1: I Don't Know

Reviewer #2: Yes

**********

3. Have the authors made all data underlying the findings in their manuscript fully available?

The PLOS Data policy requires authors to make all data underlying the findings described in their manuscript fully available without restriction, with rare exception (please refer to the Data Availability Statement in the manuscript PDF file). The data should be provided as part of the manuscript or its supporting information, or deposited to a public repository. For example, in addition to summary statistics, the data points behind means, medians and variance measures should be available. If there are restrictions on publicly sharing data—e.g. participant privacy or use of data from a third party—those must be specified.

Reviewer #1: No

Reviewer #2: No

**********

4. Is the manuscript presented in an intelligible fashion and written in standard English?

PLOS ONE does not copyedit accepted manuscripts, so the language in submitted articles must be clear, correct, and unambiguous. Any typographical or grammatical errors should be corrected at revision, so please note any specific errors here.

Reviewer #1: Yes

Reviewer #2: Yes

**********

5. Review Comments to the Author

Please use the space provided to explain your answers to the questions above. You may also include additional comments for the author, including concerns about dual publication, research ethics, or publication ethics. (Please upload your review as an attachment if it exceeds 20,000 characters)

Reviewer #1: This study addresses an important clinical problem. Postoperative delirium remains underdetected, and objective EEG markers could improve screening. The concept of using 1 Hz spectral resolution instead of traditional broad frequency bands is innovative and clinically relevant. The manuscript is generally well organized, and the EEG preprocessing and statistical methods are described with reasonable transparency.

However, there are important methodological and interpretative issues that limit scientific rigor in its current form. The main concerns relate to small sample size, risk of false positive findings due to multiple comparisons, lack of validation of diagnostic metrics, and overstatement of conclusions. The study has potential for publication if it is reframed clearly as an exploratory pilot investigation and if the claims are moderated accordingly.

Major concerns

Sample size and statistical power

The study includes 47 patients, of whom only 7 developed delirium. All delirium cases occurred at T2, and only 2 delirium patients contributed data at T3 and T4. This creates several issues:

Strong group imbalance

Very low event count

High instability in effect estimates

Very limited reliability of later timepoint analyses

Although FDR correction was applied, the combination of small n and many comparisons increases the risk of false discoveries. The manuscript must clearly state that this is an exploratory pilot study. T3 and T4 findings should be described as preliminary observations only. Any physiological interpretation based on two patients must be toned down or moved to supplementary material.

A brief discussion of statistical power would strengthen the paper. Even a simple post hoc estimation acknowledging limited power to detect small or moderate effects would improve transparency.

Multiple comparisons and frequency level interpretation

At each timepoint, 45 frequency bins were tested. The manuscript reports 26 significant frequencies at baseline and 27 at T2. Even with FDR correction, the large number of positive bins raises concern for type I error, especially with small sample size.

The authors should:

Explicitly state the total number of tests performed

Clarify how FDR correction was applied at each timepoint

Focus interpretation on consistent effect size patterns rather than number of significant bins

Avoid attributing physiological meaning to every significant frequency

It would improve rigor to highlight only frequencies with both statistical significance and medium or larger effect sizes, and to emphasize patterns rather than isolated peaks.

Diagnostic performance and risk of optimism bias

ROC curves and optimal thresholds were derived and tested in the same dataset. With only 7 delirium cases, AUROC values such as 0.74 may be optimistic.

The manuscript should clearly state:

No cross validation was performed

No external validation cohort was used

Thresholds were optimized on the same sample

Diagnostic metrics are hypothesis generating

It would be inappropriate to imply that 23 Hz is a validated classifier. The language should reflect exploratory signal identification rather than clinical readiness.

Interpretation of theta suppression

Most prior delirium EEG studies report increased theta and delta power. This study reports reduced theta in delirium patients. This is interesting but must be presented cautiously.

The authors should discuss:

Whether relative power normalization influenced findings

Possible impact of the mental counting task on theta activity

Whether absolute power analyses were performed

Alternative explanations beyond population specificity

Currently, the manuscript leans toward interpreting this as a spinal surgery specific phenotype. That conclusion should be softened unless stronger evidence is provided.

Confounding variables

The study does not adjust for perioperative variables such as:

Duration of surgery

Blood loss

Steroid exposure

Opioid dose

Type of procedure

Baseline cognitive reserve

Even a simple logistic regression adjusting for age and sex would strengthen credibility. Without adjustment, it is not possible to know whether spectral differences reflect delirium itself or perioperative factors.

Moderate concerns

Definition of delirium group

Delirium is defined as CAM positive at any postoperative timepoint. In practice, all cases occurred at T2. This should be clarified clearly in the Methods section, not only in Results.

Baseline predictive claims

The manuscript reports preoperative spectral differences and implies predictive potential. With only 7 future delirium cases and no predictive model, this should be framed as preliminary signal exploration rather than prediction.

Data availability

The data availability statement indicates restrictions. PLOS One requires strong justification for restricted data. The authors should clarify whether deidentified spectral data can be shared.

Single electrode limitation

Using Fp1 only limits spatial interpretation. The manuscript should explicitly state that connectivity and regional dynamics cannot be assessed and that spectral resolution does not replace spatial information.

Minor issues

Language and clarity

Some sections contain repeated phrases or long speculative paragraphs. The discussion would benefit from tightening and removal of redundant sentences.

Overstatement of conclusions

Phrases such as identifying discrete EEG biomarkers or outperforming conventional bands should be softened. Suggested wording:

Instead of stating that high resolution analysis identifies biomarkers, state that it identified candidate frequency specific features in this dataset.

Instead of stating that conventional bands mask physiologically meaningful signatures, state that conventional bands did not detect certain frequency specific differences observed in this sample.

Figure interpretation

The 1 Hz heatmap is visually dense. Consider adding a simplified summary figure highlighting key frequencies with medium or larger effects.

Strengths

Clear EEG acquisition and preprocessing description

Use of non parametric statistics

Reporting of effect sizes

Use of FDR correction

Transparent acknowledgment of small sample in limitations

Relevant comparison with prior EEG delirium literature

Focus on spinal surgery population

Recommended revisions before submission

Reframe the study explicitly as an exploratory pilot investigation.

Reduce emphasis on T3 and T4 findings or move them to supplementary material.

Moderate all predictive and diagnostic claims.

Add a paragraph discussing optimism bias in ROC analysis.

Consider adding a simple adjusted analysis controlling for age and sex.

Shorten speculative physiological explanations not directly supported by data.

Strengthen the limitations section to emphasize small sample and need for validation.

Publication potential

In its current form, the manuscript requires major revision. After reframing and moderating conclusions, it could meet the methodological transparency standards of PLOS One as an exploratory study. It would not yet support strong biomarker claims without external validation.

Reviewer #2:

Overall evaluation

This study used single-channel Fp1 electroencephalography and 1 Hz resolution spectral analysis to investigate the neurophysiological characteristics of delirium in patients undergoing spinal surgery. This topic is clinically relevant, particularly in attempting to identify low-cost and readily available screening methods, and in exploring frequency-specific biomarkers (such as 23 Hz) beyond traditional band analysis.

However, several important methodological and statistical limitations significantly weaken the strength of the conclusions. In particular, the extremely low number of delirium cases raises concerns about the robustness, stability, and generalizability of the reported results. The manuscript requires extensive revisions before publication.

Main problems

1. Sample size, number of events, and statistical stability

With a limited sample size (N = 47), only 7 cases (14.9%) were diagnosed with delirium. More importantly, only 2 cases of delirium remained in the later stages (T3 and T4). Under these circumstances, any diagnostic performance estimate would be highly unstable and prone to random variation.

Importantly, this limitation is not limited to T3/T4 analyses. Even major findings (such as AUROC in T2) can be affected by significant uncertainties due to the extremely small number of events.

Recommendation:

(1) The author should explicitly acknowledge that the statistical power of the effect estimates in the whole manuscript is limited and unstable.

(2) The findings of T3 and T4 should be clearly described as exploratory and should not be used to support confirmatory claims.

(3) The main conclusions should be strictly limited to the most reliable time point (T2), and even then, they should be interpreted with caution.

2. Confounding Factors and Limitations of Adjustment

The sex distribution was significantly unbalanced (71.4% male in the delirium group and 52.5% in the control group), and key variables such as age and baseline cognitive function (MMSE) were maturation predictors of delirium. These factors may have seriously confounded the reported association between ghost characteristics and delirium.

Recommendation:

The authors might consider adjusted analyses (such as logistic regression or ANCOVA) to explore whether the observed spectral features still have diagnostic value after accounting for potential confounding factors. However, given that there were only seven delirium events, any multivariate model may appear weak and unstable, especially with a limited ratio of events to variables.

Therefore:

Any adjusted analysis, if conducted, should only be performed as an exploratory step.

The lack of robust confounding adjustment should be more explicitly acknowledged as a limitation.

3. Interpretation of diagnostic manifestations

The manuscript emphasizes an AUROC of 0.74 for the 23 Hz feature. However, in clinical diagnosis, this level of discrimination is usually considered moderate rather than strong. Furthermore, given the small number of cases, the estimated AUROC and the optimal cutoff value derived from Youden's J exponent are highly susceptible to overfitting.

Recommendation:

(1) The author should exercise restraint in claiming the “superiority” of a specific frequency over the traditional strip method.

(2) The confidence interval of AUROC should be clearly reported and discussed.

(3) If feasible, internal validation (such as exclusion-exclusion cross-validation) should be performed to assess the robustness of the findings.

4. Data availability

The current statement regarding the inability to share data due to ethical restrictions does not comply with PLOS ONE's data availability standards.

Minor revisions

Figure 5: The ROC curves for T3 and T4 should be removed because the analysis based on N=2 is statistically meaningless.

Terminology: Replace assertive terms such as “proof” with more appropriate language (e.g., “recommend” or “related to”).

Limitations: The manuscript should discuss the limitations more prominently and clearly, especially the small sample size, overfitting, and the risk of false positives.

in conclusion

This study explores an interesting and clinically significant question and introduces a potentially useful analytical approach. However, the rarity of delirium cases greatly limits the validity and interpretability of the findings.

After significant revisions, the author may consider publication, provided that the author:

1. Appropriately limit their conclusions.

2. Explicitly acknowledge the limitations of statistics, and

3. Enhance the methodological rigor and transparency of analysis.

**********

6. PLOS authors have the option to publish the peer review history of their article (what does this mean?). If published, this will include your full peer review and any attached files.

If you choose “no”, your identity will remain anonymous but your review may still be made public.

**Do you want your identity to be public for this peer review?**  For information about this choice, including consent withdrawal, please see our Privacy Policy.

Reviewer #1: No

Reviewer #2: **Yes:**  向大

**********

[NOTE: If reviewer comments were submitted as an attachment file, they will be attached to this email and accessible via the submission site. Please log into your account, locate the manuscript record, and check for the action link "View Attachments". If this link does not appear, there are no attachment files.]

To ensure your figures meet our technical requirements, please review our figure guidelines: https://journals.plos.org/plosone/s/figures

You may also use PLOS’s free figure tool, NAAS, to help you prepare publication quality figures: https://journals.plos.org/plosone/s/figures#loc-tools-for-figure-preparation.

NAAS will assess whether your figures meet our technical requirements by comparing each figure against our figure specifications.

Attachment Submitted filename: PONE-D-25-68816 reviewer with revision notes.docx

Attachment Submitted filename: Reviewer Comments for PONE.docx

# Author comment: Author response to Decision Letter 1

**DOI:** 10.1371/journal.pone.0352607.r002

9 May 2026

Response to Reviewers

Manuscript ID: PONE-D-25-68816

We sincerely thank both reviewers for their constructive feedback. The comments led us to rework the framing, the statistical reporting, and the interpretation of our findings. The study is now presented as an exploratory pilot investigation, the diagnostic claims have been moderated, and the limitations are stated more directly. Reviewer comments are reproduced in italics below, followed by our response.

Reviewer #1

Major Concerns

1. Sample size and statistical power

Comment:

The study includes 47 patients, of whom only 7 developed delirium. All delirium cases occurred at T2, and only 2 delirium patients contributed data at T3 and T4. The manuscript must clearly state that this is an exploratory pilot study. T3 and T4 findings should be described as preliminary observations only. Any physiological interpretation based on two patients must be toned down or moved to supplementary material. A brief discussion of statistical power would strengthen the paper.

Response:

We agree. The Abstract, Introduction, and Conclusion now describe the work as a pilot study, and the small sample is treated as the primary constraint throughout.

T3 and T4 are now reported as descriptive observations from two patients. Physiological claims that depending on those two patients have been removed, and the diagnostic performance evaluation is restricted to T2 (Fig 5 and Table 6). The within-subject T1–T4 trajectory is retained because both patients with available recordings showed the same direction of change, but it is clearly labeled as such.

On power, a post-hoc power analysis has been added to the Limitations section. With the current sample, the study had approximately 80% power to detect only large between-group differences (correlation coefficient of about 0.52 or larger), and power to detect moderate effects in the range of |r| = 0.20 to 0.30 was below 40%. We also estimate that a future confirmatory study targeting a moderate effect of |r| = 0.30 at 80% power, with a similar group ratio, would require approximately 167 patients with at least 25 delirium events.

2. Multiple comparisons and frequency-level interpretation

Comment:

At each timepoint, 45 frequency bins were tested. Even with FDR correction, the large number of positive bins raises concern for type I error. The authors should explicitly state the total number of tests performed, clarify how FDR correction was applied at each timepoint, focus interpretation on consistent effect size patterns rather than number of significant bins, and avoid attributing physiological meaning to every significant frequency.

Response:

The Methods section now explicitly states the total test count: 180 tests for the 1-Hz analysis (45 frequencies × 4 timepoints) and 20 tests for the band analysis (5 bands × 4 timepoints). FDR correction was applied within each timepoint, separately for the band-level and the 1-Hz analyses, since each timepoint represents a different clinical state.

The Results and Discussion section focuses on three coherent patterns at T2: a 1 Hz elevation, a 3–7 Hz reduction, and a low-beta elevation around 14–16 Hz. Fig 4 shows only frequencies meeting both FDR significance and |r| ≥ 0.20, which makes the consistent patterns visible without the visual clutter of small-effect bins. The full 45-frequency heatmap is moved to Fig S6.

3. Diagnostic performance and risk of optimism bias

Comment:

ROC curves and optimal thresholds were derived and tested in the same dataset. With only 7 delirium cases, AUROC values such as 0.74 may be optimistic. The manuscript should clearly state: no cross validation was performed, no external validation cohort was used, thresholds were optimized on the same sample, diagnostic metrics are hypothesis generating.

Response:

All four points are now stated in the Limitations section. The AUROC is described as moderate rather than strong, the 95% CI is reported alongside the point estimate (AUROC = 0.74, 95% CI 0.71–0.77), and the threshold was identified and evaluated in the same dataset. The 23 Hz feature is described as a candidate for future validation, not as a classifier ready for clinical use.

We considered leave-one-out cross-validation but did not include it in the revised manuscript. With seven cases, leave-one-out at the patient level removes one of seven events at each fold, and the cross-validated estimate carries the same instability as the in-sample one. External validation in a larger cohort is identified as the appropriate next step.

4. Interpretation of theta suppression

Comment:

Most prior delirium EEG studies report increased theta and delta power. This study reports reduced theta in delirium patients. The authors should discuss: whether relative power normalization influenced findings, possible impact of the mental counting task on theta activity, whether absolute power analyses were performed, alternative explanations beyond population specificity.

Response:

This part of the Discussion has been rewritten. The reduction in theta is no longer presented as a clean spinal-surgery phenotype, and two alternative explanations are discussed alongside it.

First, the mental counting task. Frontal midline theta increases during mental arithmetic, so reduced engagement with the task in delirium patients could itself produce a theta reduction unrelated to resting-state activity.

Second, the relative versus absolute power question. We ran the absolute power analysis to check. Both relative and absolute power show theta reduction at T2 in the same direction (Table S4), so the finding is less likely to be an artifact generated from normalization. This is noted in the Discussion.

5. Confounding variables

Comment:

The study does not adjust for perioperative variables such as duration of surgery, blood loss, steroid exposure, opioid dose, type of procedure, baseline cognitive reserve. Even a simple logistic regression adjusting for age and sex would strengthen credibility.

Response:

A post-hoc ANCOVA with age and sex as covariates has been added, applied to all features that reached FDR significance in the primary analysis. The results are reported in the Results section.

The outcome was informative. At the band level, the T1 and T2 theta differences did not survive adjustment, while T2 gamma did. At the 1-Hz level, the picture was different. The 22–25 Hz cluster at T2 remained significant after adjustment, with partial η² = 0.26–0.31 across these four bins, and the 23 Hz feature held up at partial η² = 0.311. Reductions in the low-frequency bins (2–4 Hz) also persisted.

The adjustment was not extended to the perioperative variable list. With seven events, even the two-covariate model is at the edge of acceptable events-per-variable, and adding more covariates produces unstable estimates. This is stated in the Limitations, and broader adjustment is identified as a task for the validation cohort.

Moderate Concerns

6. Definition of delirium group

Comment:

Delirium is defined as CAM positive at any postoperative timepoint. In practice, all cases occurred at T2. This should be clarified clearly in the Methods section, not only in Results.

Response:

We appreciate this comment. The Methods section now clarifies this as: delirium was defined as CAM-positive at any postoperative assessment, but in this cohort all seven cases were CAM-positive at T2 only, with no new incident cases at T3 or T4.

7. Baseline predictive claims

Comment:

The manuscript reports preoperative spectral differences and implies predictive potential. With only 7 future delirium cases and no predictive model, this should be framed as preliminary signal exploration rather than prediction.

Response:

The predictive framing has been removed from the T1 results. The context now describes T1 differences as preliminary observations of baseline spectral patterns in patients who later did and did not develop delirium. We do not claim a predictive model for the T1 findings.

8. Data availability

Comment:

The data availability statement indicates restrictions. PLOS One requires strong justification for restricted data. The authors should clarify whether deidentified spectral data can be shared.

Response:

The Data Availability statement has been revised. Raw EEG recordings remain restricted under the original IRB-approved consent at Keio University Hospital, but readers can request access through the Keio School of Medicine Ethics Committee, subject to a data use agreement and local approval. Please contact the administrative officer Dr. Sumitaka Hase, via shase@adst.keio.ac.jp.

9. Single-electrode limitation

Comment:

Using Fp1 only limits spatial interpretation. The manuscript should explicitly state that connectivity and regional dynamics cannot be assessed and that spectral resolution does not replace spatial information.

Response:

This is now stated in the Limitations. Single-channel Fp1 recordings cannot support connectivity analyses, source localization, or any inference about posterior or temporal regions, and spectral resolution does not substitute for spatial information. Fp1 was selected because single-electrode recording is realistic for routine ward use, and the trade-off is acknowledged as such.

Minor Issues

10. Language and clarity

Comment:

Some sections contain repeated phrases or long speculative paragraphs. The discussion would benefit from tightening and removal of redundant sentences.

Response:

Thank you for your comment. We have revised the document accordingly.

11. Overstatement of conclusions

Comment:

Phrases such as identifying discrete EEG biomarkers or outperforming conventional bands should be softened. Suggested wording: candidate frequency-specific features in this dataset; conventional bands did not detect certain frequency-specific differences observed in this sample.

Response:

We appreciate the suggestion and we realize some of our wording might be misleading. The revision was made to those phrases.

12. Figure interpretation

Comment:

The 1 Hz heatmap is visually dense. Consider adding a simplified summary figure highlighting key frequencies with medium or larger effects.

Response:

We agreed. Fig 4 has been redrawn to show only the 20 frequencies meeting both FDR significance and |r| ≥ 0.20 across timepoints, grouped by conventional band. The full 45-frequency heatmap is retained as Fig S6. The simplified figure which highlights the principal patterns (3–7 Hz reduction, 14–16 Hz elevation, 23 Hz peak at T2) should be easier to read now.

Reviewer #2

Reviewer 2's concerns overlap closely with those raised by Reviewer 1. Where the underlying issue is the same, we cross-reference rather than repeat. We appreciate your time for reading our response.

Main Problems

1. Sample size, number of events, and statistical stability

Comment:

With a limited sample size (N = 47), only 7 cases (14.9%) were diagnosed with delirium. Only 2 cases of delirium remained in the later stages (T3 and T4). Recommendation: (1) explicitly acknowledge limited and unstable statistical power throughout. (2) describe T3 and T4 findings as exploratory only. (3) restrict main conclusions to T2.

Response:

Implemented as described in our response to Reviewer 1, Major Concern 1. The framing is now exploratory throughout, T3 and T4 are descriptive observations from two patients only, and the diagnostic performance evaluation is restricted to T2. A post-hoc power analysis has been added showing ~80% power only for large effects (|r| ≥ 0.52) and below 40% power for moderate effects (|r| = 0.20–0.30).

2. Confounding factors and limitations of adjustment

Comment:

The sex distribution was unbalanced (71.4% male in the delirium group and 52.5% in the control group). Age and baseline MMSE are also predictors of delirium. Recommendation: consider adjusted analyses, but treat them as exploratory given seven events. Acknowledge the lack of robust adjustment as a limitation.

Response:

Addressed as described in our response to Reviewer 1, Major Concern 5. A post-hoc ANCOVA with age and sex was added, applied only to features that reached FDR significance in the primary analysis. The 22–25 Hz cluster at T2, including the 23 Hz feature, survived adjustment with partial η² = 0.26–0.31; band-level theta did not. The Limitations section notes that broader perioperative adjustment was not feasible with seven events.

3. Interpretation of diagnostic performance

Comment:

AUROC of 0.74 is moderate rather than strong. With small N, the AUROC and the Youden cutoff are susceptible to overfitting. Recommendation: temper claims about superiority, report AUROC confidence intervals, and consider internal validation.

Response:

Comparative language has been softened, and 95% CIs are reported for AUROC, sensitivity, specificity, PPV, NPV, and likelihood ratios in Table 6. The in-sample, hypothesis-generating nature of the metrics is stated in the Limitations. Leave-one-out CV was considered but not included, as explained in our response to Reviewer 1, Major Concern 3.

4. Data availability

Comment:

The current statement does not comply with PLOS ONE's data availability standards.

Response:

Addressed as described in our response to Reviewer 1, Moderate Concern 8. Raw recordings and processed data remain restricted under the original consent but can be requested through the institutional ethics committee. Please contact the administrative officer Dr. Sumitaka Hase, via shase@adst.keio.ac.jp.

Minor Revisions

• Comment:

The ROC curves for T3 and T4 should be removed because the analysis based on N=2 is statistically meaningless.

Response:

Thank you for your suggestion. Fig 5 now shows only the T2 ROC curves, comparing the best 1-Hz feature (23 Hz) with the best traditional band (beta). The figure caption and the corresponding Results text have been updated.

• Comment:

Replace assertive terms such as "proof" with more appropriate language.

Response:

Thank you for your suggestion, and we described in our response to Reviewer 1, Minor Issue 11. Assertive terms have been replaced with hedged equivalents, and “biomarkers” is now “candidate features.”

• Comment:

The manuscript should discuss the limitations more prominently, especially small sample size, overfitting, and the risk of false positives.

Response:

The Limitations section has been fully rewritten. It now opens with the small number of events as the primary constraint, including the post-hoc power analysis described above, addresses overfitting from in-sample threshold selection using Youden's J, and discusses false-positive risk despite FDR correction. The low PPV at T2 is contextualized as a consequence of the 14.9% prevalence rather than poor classifier performance, and the practical role of these features is described as rule-out screening rather than diagnostic confirmation.

Attachment Submitted filename: Response to Reviewers.docx

# Peer review: Decision Letter 1

**DOI:** 10.1371/journal.pone.0352607.r003

**Authors:** Hiroki Annaka, Hiroki Annaka

21 May 2026

PONE-D-25-68816R1

EEG spectral biomarkers of postoperative delirium in spinal surgery: a high-resolution analysis

PLOS One

Dear Dr. Mitsukura,

Thank you for submitting your manuscript to PLOS ONE. After careful consideration, we feel that it has merit but does not fully meet PLOS ONE’s publication criteria as it currently stands. Therefore, we invite you to submit a revised version of the manuscript that addresses the points raised during the review process.

==============================

Please make the revisions based on Reviewer 1’s comments.

I understand that you are making changes related to “Data Availability,” but please include the details of those changes in the “Data Availability” on editorial manager.

==============================

Please submit your revised manuscript by Jul 05 2026 11:59PM. If you will need more time than this to complete your revisions, please reply to this message or contact the journal office at plosone@plos.org. When you're ready to submit your revision, log on to https://www.editorialmanager.com/pone/ and select the 'Submissions Needing Revision' folder to locate your manuscript file.

Please include the following items when submitting your revised manuscript:

- A letter that responds to each point raised by the academic editor and reviewer(s). You should upload this letter as a separate file labeled 'Response to Reviewers'.
- A marked-up copy of your manuscript that highlights changes made to the original version. You should upload this as a separate file labeled 'Revised Manuscript with Track Changes'.
- An unmarked version of your revised paper without tracked changes. You should upload this as a separate file labeled 'Manuscript'.

If you would like to make changes to your financial disclosure, please include your updated statement in your cover letter. Guidelines for resubmitting your figure files are available below the reviewer comments at the end of this letter.

If applicable, we recommend that you deposit your laboratory protocols in protocols.io to enhance the reproducibility of your results. Protocols.io assigns your protocol its own identifier (DOI) so that it can be cited independently in the future. For instructions see: https://journals.plos.org/plosone/s/submission-guidelines#loc-laboratory-protocols. Additionally, PLOS ONE offers an option for publishing peer-reviewed Lab Protocol articles, which describe protocols hosted on protocols.io. Read more information on sharing protocols at https://plos.org/protocols?utm_medium=editorial-email&utm_source=authorletters&utm_campaign=protocols.

As the corresponding author, your ORCID iD is verified in the submission system and will appear in the published article. PLOS supports the use of ORCID, and we encourage all coauthors to register for an ORCID iD and use it as well. Please encourage your coauthors to verify their ORCID iD within the submission system before final acceptance, as unverified ORCID iDs will not appear in the published article. *Only*  the individual author can complete the verification step; PLOS staff *cannot*  verify ORCID iDs on behalf of authors.

We look forward to receiving your revised manuscript.

Kind regards,

Hiroki Annaka

Academic Editor

PLOS One

**Journal Requirements:**

1. If the reviewer comments include a recommendation to cite specific previously published works, please review and evaluate these publications to determine whether they are relevant and should be cited. There is no requirement to cite these works unless the editor has indicated otherwise.

2. Please review your reference list to ensure that it is complete and correct. If you have cited papers that have been retracted, please include the rationale for doing so in the manuscript text, or remove these references and replace them with relevant current references. Any changes to the reference list should be mentioned in the rebuttal letter that accompanies your revised manuscript. If you need to cite a retracted article, indicate the article’s retracted status in the References list and also include a citation and full reference for the retraction notice.

[Note: HTML markup is below. Please do not edit.]

Reviewers' comments:

Reviewer's Responses to Questions

**Comments to the Author**

1. If the authors have adequately addressed your comments raised in a previous round of review and you feel that this manuscript is now acceptable for publication, you may indicate that here to bypass the “Comments to the Author” section, enter your conflict of interest statement in the “Confidential to Editor” section, and submit your "Accept" recommendation.

Reviewer #1: All comments have been addressed

Reviewer #2: All comments have been addressed

**********

2. Is the manuscript technically sound, and do the data support the conclusions?

The manuscript must describe a technically sound piece of scientific research with data that supports the conclusions. Experiments must have been conducted rigorously, with appropriate controls, replication, and sample sizes. The conclusions must be drawn appropriately based on the data presented.

Reviewer #1: Yes

Reviewer #2: Yes

**********

3. Has the statistical analysis been performed appropriately and rigorously?

Reviewer #1: Yes

Reviewer #2: Yes

**********

4. Have the authors made all data underlying the findings in their manuscript fully available?

The PLOS Data policy requires authors to make all data underlying the findings described in their manuscript fully available without restriction, with rare exception (please refer to the Data Availability Statement in the manuscript PDF file). The data should be provided as part of the manuscript or its supporting information, or deposited to a public repository. For example, in addition to summary statistics, the data points behind means, medians and variance measures should be available. If there are restrictions on publicly sharing data—e.g. participant privacy or use of data from a third party—those must be specified.

Reviewer #1: No

Reviewer #2: Yes

**********

5. Is the manuscript presented in an intelligible fashion and written in standard English?

PLOS ONE does not copyedit accepted manuscripts, so the language in submitted articles must be clear, correct, and unambiguous. Any typographical or grammatical errors should be corrected at revision, so please note any specific errors here.

Reviewer #1: Yes

Reviewer #2: Yes

**********

6. Review Comments to the Author

Please use the space provided to explain your answers to the questions above. You may also include additional comments for the author, including concerns about dual publication, research ethics, or publication ethics. (Please upload your review as an attachment if it exceeds 20,000 characters)

**Reviewer #1:**  The authors have substantially improved the manuscript and have addressed the major concerns raised during the previous round of review. The revised version now presents the study appropriately as an exploratory pilot investigation, with clearer acknowledgment of the limitations imposed by the small sample size and the limited number of delirium events. The conclusions are more balanced, and the discussion now better reflects the hypothesis-generating nature of the findings.

The statistical reporting has been strengthened considerably through the addition of effect sizes, confidence intervals, clarification of FDR correction procedures, and discussion of optimism bias related to in-sample threshold optimization. The added post-hoc power analysis and the cautious interpretation of AUROC values substantially improve the transparency and rigor of the work. I also appreciate the authors’ restraint in limiting covariate adjustment to exploratory analyses given the low number of events.

The revised manuscript is technically sound overall, and the data support the conclusions when interpreted within the stated exploratory framework. The discussion of theta suppression and the consideration of alternative explanations, including the mental counting task and relative versus absolute power normalization, are appropriate and strengthen the interpretation of the findings.

I have only a few remaining minor comments:

Some wording in the Abstract and Conclusions remains slightly stronger than warranted for a pilot exploratory study. Phrases such as “identifies discrete EEG biomarkers” and “outperform conventional frequency bands” could still be softened further (e.g., “identifies candidate frequency-specific EEG features in this dataset”).

Although the authors appropriately acknowledge the very limited sample at T3 and T4, parts of the interpretation of these later timepoints remain somewhat extensive considering only two delirium patients contributed data. I recommend continuing to emphasize that these observations are descriptive and preliminary.

Minor English editing and copyediting would further improve readability. A few grammatical issues and awkward phrasings remain throughout the manuscript.

The Data Availability statement has improved substantially; however, the authors should ensure that it fully complies with PLOS ONE policy requirements regarding accessibility of underlying data.

Overall, I believe the manuscript has improved significantly and now represents a valuable exploratory contribution to the literature on EEG-based assessment of postoperative delirium in spinal surgery patients.

**Reviewer #2:**  (No Response)

**********

7. PLOS authors have the option to publish the peer review history of their article (what does this mean?). If published, this will include your full peer review and any attached files.

If you choose “no”, your identity will remain anonymous but your review may still be made public.

**Do you want your identity to be public for this peer review?**  For information about this choice, including consent withdrawal, please see our Privacy Policy.

Reviewer #1: No

Reviewer #2: **Yes:**  Xiang Da

**********

[NOTE: If reviewer comments were submitted as an attachment file, they will be attached to this email and accessible via the submission site. Please log into your account, locate the manuscript record, and check for the action link "View Attachments". If this link does not appear, there are no attachment files.]

To ensure your figures meet our technical requirements, please review our figure guidelines: https://journals.plos.org/plosone/s/figures

You may also use PLOS’s free figure tool, NAAS, to help you prepare publication quality figures: https://journals.plos.org/plosone/s/figures#loc-tools-for-figure-preparation.

NAAS will assess whether your figures meet our technical requirements by comparing each figure against our figure specifications.

# Author comment: Author response to Decision Letter 2

**DOI:** 10.1371/journal.pone.0352607.r004

5 Jun 2026

Response to Reviewers

Manuscript ID: PONE-D-25-68816

We sincerely appreciate both the careful and constructive assessment and thank you for recognizing the improvement we have made since last revision. We have addressed each remaining minor point below.

Reviewer #1

1. Comment:

Wording in Abstract and Conclusions still slightly stronger than warranted (e.g., "identifies discrete EEG biomarkers," "outperform conventional frequency bands")

Response:

We appreciate this comment. We have further softened the wording in the Abstract and Conclusions as suggested.

2. Comment:

Although the authors appropriately acknowledge the very limited sample at T3 and T4, parts of the interpretation of these later timepoints remain somewhat extensive considering only two delirium patients contributed data. I recommend continuing to emphasize that these observations are descriptive and preliminary.

Response:

We have revised the relevant passages so that the T3 and T4 findings are presented as descriptive and preliminary observations from a very limited number of patients, and we have removed interpretive statements that extend beyond what the data support.

3. Comment:

Minor English editing and copyediting would further improve readability.

Response:

We have carried out a copyediting pass throughout the manuscript and hopefully it will improve the general readability.

4. Comment:

The Data Availability statement has improved substantially; however, the authors should ensure that it fully complies with PLOS ONE policy requirements regarding accessibility of underlying data.

Response:

Thank you for pointing this out. Yes, we have updated our data availability statement and corresponding contact information of the Ethics Committee. The data would be made available on reasonable request, following by screening and approval from the Ethics Committee.

Attachment Submitted filename: Response to Reviewers_rev2_0526.docx

# Peer review: Decision Letter 2

**DOI:** 10.1371/journal.pone.0352607.r005

**Authors:** Hiroki Annaka, Hiroki Annaka, Hiroki Annaka

12 Jun 2026

EEG spectral biomarkers of postoperative delirium in spinal surgery: a high-resolution analysis

PONE-D-25-68816R2

Dear Dr. Mitsukura,

We’re pleased to inform you that your manuscript has been judged scientifically suitable for publication and will be formally accepted for publication once it meets all outstanding technical requirements.

Within one week, you’ll receive an e-mail detailing the required amendments. When these have been addressed, you’ll receive a formal acceptance letter and your manuscript will be scheduled for publication.

An invoice will be generated when your article is formally accepted. Please note, if your institution has a publishing partnership with PLOS and your article meets the relevant criteria, all or part of your publication costs will be covered. Please make sure your user information is up-to-date by logging into Editorial Manager at Editorial Manager® and clicking the ‘Update My Information' link at the top of the page. For questions related to billing, please contact billing support.

If your institution or institutions have a press office, please notify them about your upcoming paper to help maximize its impact. If they’ll be preparing press materials, please inform our press team as soon as possible -- no later than 48 hours after receiving the formal acceptance. Your manuscript will remain under strict press embargo until 2 pm Eastern Time on the date of publication. For more information, please contact onepress@plos.org.

Kind regards,

Hiroki Annaka

Academic Editor

PLOS One

Additional Editor Comments (optional):

Reviewers' comments:

Reviewer's Responses to Questions

**Comments to the Author**

1. If the authors have adequately addressed your comments raised in a previous round of review and you feel that this manuscript is now acceptable for publication, you may indicate that here to bypass the “Comments to the Author” section, enter your conflict of interest statement in the “Confidential to Editor” section, and submit your "Accept" recommendation.

Reviewer #1: All comments have been addressed

**********

2. Is the manuscript technically sound, and do the data support the conclusions?

The manuscript must describe a technically sound piece of scientific research with data that supports the conclusions. Experiments must have been conducted rigorously, with appropriate controls, replication, and sample sizes. The conclusions must be drawn appropriately based on the data presented.

Reviewer #1: Yes

**********

3. Has the statistical analysis been performed appropriately and rigorously?

Reviewer #1: Yes

**********

4. Have the authors made all data underlying the findings in their manuscript fully available?

The PLOS Data policy requires authors to make all data underlying the findings described in their manuscript fully available without restriction, with rare exception (please refer to the Data Availability Statement in the manuscript PDF file). The data should be provided as part of the manuscript or its supporting information, or deposited to a public repository. For example, in addition to summary statistics, the data points behind means, medians and variance measures should be available. If there are restrictions on publicly sharing data—e.g. participant privacy or use of data from a third party—those must be specified.

Reviewer #1: Yes

**********

5. Is the manuscript presented in an intelligible fashion and written in standard English?

PLOS ONE does not copyedit accepted manuscripts, so the language in submitted articles must be clear, correct, and unambiguous. Any typographical or grammatical errors should be corrected at revision, so please note any specific errors here.

Reviewer #1: Yes

**********

6. Review Comments to the Author

Please use the space provided to explain your answers to the questions above. You may also include additional comments for the author, including concerns about dual publication, research ethics, or publication ethics. (Please upload your review as an attachment if it exceeds 20,000 characters)

Reviewer #1: The authors have satisfactorily addressed the concerns raised in the previous review. The revised manuscript presents a more balanced interpretation of the findings, with appropriately tempered conclusions and clearer acknowledgment of the exploratory nature of the study. In particular, the authors have revised the Abstract and Conclusions to avoid overstating the diagnostic utility of the reported EEG features and have appropriately emphasized the limitations related to the small sample size and the very limited number of delirium patients available at later postoperative timepoints.

The discussion of T3 and T4 findings has been revised to reflect their preliminary and descriptive nature, which is more consistent with the available data. The Data Availability Statement has also been improved and now provides a clear mechanism for data access while accounting for ethical restrictions associated with human participant data.

The study remains limited by the small number of delirium cases and the lack of external validation of the proposed diagnostic thresholds; however, these limitations are clearly acknowledged by the authors and do not detract from the value of this exploratory investigation. The manuscript provides a thoughtful analysis of high-resolution EEG spectral features and contributes useful preliminary evidence supporting further research in this area.

I have only minor editorial observations regarding language and readability, which can be addressed during the production process if necessary. Overall, I believe the manuscript is suitable for publication in its current form.

**********

7. PLOS authors have the option to publish the peer review history of their article (what does this mean?). If published, this will include your full peer review and any attached files.

If you choose “no”, your identity will remain anonymous but your review may still be made public.

**Do you want your identity to be public for this peer review?**  For information about this choice, including consent withdrawal, please see our Privacy Policy.

Reviewer #1: No

**********

# Editor report: Acceptance letter

**DOI:** 10.1371/journal.pone.0352607.r006

**Authors:** Hiroki Annaka, Hiroki Annaka, Hiroki Annaka

PONE-D-25-68816R2

PLOS One

Dear Dr. Mitsukura,

I'm pleased to inform you that your manuscript has been deemed suitable for publication in PLOS One. Congratulations! Your manuscript is now being handed over to our production team.

At this stage, our production department will prepare your paper for publication. This includes ensuring the following:

* All references, tables, and figures are properly cited

* All relevant supporting information is included in the manuscript submission,

* There are no issues that prevent the paper from being properly typeset

You will receive further instructions from the production team, including instructions on how to review your proof when it is ready. Please keep in mind that we are working through a large volume of accepted articles, so please give us a few days to review your paper and let you know the next and final steps.

Lastly, if your institution or institutions have a press office, please let them know about your upcoming paper now to help maximize its impact. If they'll be preparing press materials, please inform our press team within the next 48 hours. Your manuscript will remain under strict press embargo until 2 pm Eastern Time on the date of publication. For more information, please contact onepress@plos.org.

You will receive an invoice from PLOS for your publication fee after your manuscript has reached the completed accept phase. If you receive an email requesting payment before acceptance or for any other service, this may be a phishing scheme. Learn how to identify phishing emails and protect your accounts at https://explore.plos.org/phishing.

If we can help with anything else, please email us at customercare@plos.org.

Thank you for submitting your work to PLOS ONE and supporting open access.

Kind regards,

PLOS ONE Editorial Office Staff

on behalf of

Dr. Hiroki Annaka

Academic Editor

PLOS One
